# Protein identification using Cryo-EM and artificial intelligence guides improved sample purification

**DOI:** 10.1016/j.yjsbx.2025.100120

**Published:** 2025-01-21

**Authors:** Kenneth D. Carr, Dane Evan D. Zambrano, Connor Weidle, Alex Goodson, Helen E. Eisenach, Harley Pyles, Alexis Courbet, Neil P. King, Andrew J. Borst

**Affiliations:** a Department of Biochemistry, University of Washington, Seattle, WA 98195, USA; b Institute for Protein Design, University of Washington, Seattle, WA 98195, USA

**Keywords:** Protein Purification, Contamination, Cryo-Electron Microscopy, Cryo-EM, DLST, Dihydrolipoamide Succinyltransferase, Dihydrolipoyllysine-residue succinyltransferase, *E. coli*, Tricarboxylic Acid Cycle, TCA Cycle, ModelAngelo, Automated Model Building, Multiple Sequence Alignment, Protein BLAST, Hmmsearch, Protein Data Bank, PDB, AlphaFold 3, Structure Prediction, Western Blot

## Abstract

Protein purification is essential in protein biochemistry, structural biology, and protein design, enabling the determination of protein structures, the study of biological mechanisms, and the characterization of both natural and de novo designed proteins. However, standard purification strategies often encounter challenges, such as unintended co-purification of contaminants alongside the target protein. This issue is particularly problematic for self-assembling protein nanomaterials, where unexpected geometries may reflect novel assembly states, cross-contamination, or native proteins originating from the expression host. Here, we used an automated structure-to-sequence pipeline to first identify an unknown co-purifying protein found in several purified designed protein samples. By integrating cryo-electron microscopy (Cryo-EM), ModelAngelo’s sequence-agnostic model-building, and Protein BLAST, we identified the contaminant as dihydrolipoamide succinyltransferase (DLST). This identification was validated through comparisons with DLST structures in the Protein Data Bank, AlphaFold 3 predictions based on the DLST sequence from our E. coli expression vector, and traditional biochemical methods. The identification informed subsequent modifications to our purification protocol, which successfully excluded DLST from future preparations. To explore the potential broader utility of this approach, we benchmarked four computational methods for DLST identification across varying resolution ranges. This study demonstrates the successful application of a structure-to-sequence protein identification workflow, integrating Cryo-EM, ModelAngelo, Protein BLAST, and AlphaFold 3 predictions, to identify and ultimately help guide the removal of DLST from sample purification efforts. It highlights the potential of combining Cryo-EM with AI-driven tools for accurate protein identification and addressing purification challenges across diverse contexts in protein science.

## Highlights

• An unknown protein was consistently found in multiple de novo designed protein samples.
• The protein was identified as dihydrolipoamide succinyltransferase (DLST) using Cryo-EM, ModelAngelo, and Protein BLAST.
• DLST identification was further verified using AlphaFold 3, the Protein Data Bank, and Western Blot.
• Identification enabled rational modification of the purification protocol to exclude the contaminant.
• Benchmarking four computational methods for DLST identification revealed distinct strengths and limitations of each.

## Graphical abstract

**Figure:**
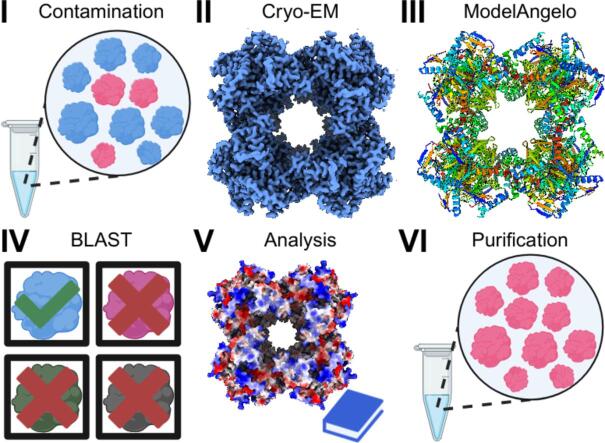

## Introduction

Understanding the details of a complex system is crucial when designing an experimental protocol, especially in the context of protein purification—a process fundamental to various scientific disciplines. The significance of protein purification lies in its ability to isolate recombinant proteins for downstream high-resolution structure determination, exploration of biochemical mechanisms, development of novel therapeutics, and the characterization of computationally designed proteins with specific functions. Despite their widespread use, protein purification protocols still face challenges, such as the co-purification of contaminant proteins from unknown origins (Bolanos-Garcia and Davies, 2006). This is particularly problematic in computational protein design, where it is often unclear whether these unknown co-purifying proteins represent off-target design states, cross-sample contamination, or naturally occurring proteins originating from the expression host.

In this study, we detail an experience encountered during the characterization of a novel computationally designed two component nanoparticle (King et al., 2014, Bale et al., 2016). During initial purification attempts, we observed an unidentified protein co-eluting with our target nanoparticle, which was also found in several unrelated designed protein samples. To identify the protein, we employed a combination of Cryo-EM Single Particle Analysis (SPA), ModelAngelo, and the Protein Basic Local Alignment Search Tool (Protein BLAST). SPA is a technique that involves imaging thousands of individual biomolecules, then aligning and averaging their 2D projections to reconstruct a high-resolution 3D structure. ModelAngelo, a machine-learning tool, automates atomic model building and aids in protein identification by analyzing sequence fragments from Cryo-EM 3D structure information (Altschul et al., 1990). ModelAngelo's ability to automatically build into Cryo-EM density maps in a sequence-agnostic manner makes it particularly well-suited for the identification of unknown proteins in complex samples where manual methods would be time-consuming or prone to errors. Protein BLAST is a publically available tool which allows users to reference protein sequences against a sequence database to find similar proteins (Altschul et al., 1990). By leveraging these tools, as well as orthogonal data from the Protein Data Bank (PDB), AlphaFold 3 (AF3), and a Western Blot, we successfully identified the contaminant protein in our sample as dihydrolipoamide succinyltransferase (DLST) (Altschul et al., 1990, Jamali et al., 2024). This unambiguous identification allowed us to rationally refine our purification protocol to eliminate the contaminant from future preparations.

Building on this success, we next assessed the broader applicability of our approach by benchmarking its effectiveness against alternative computational methods for identifying DLST in Cryo-EM maps across various resolutions, revealing distinct strengths and weaknesses in each method. Overall, the successful application of ModelAngelo and sequence-based approaches to protein identification underscores the potential of integrating structural, bioinformatic, and AI-driven tools to identify unknown proteins in complex samples. This approach offers a powerful solution for overcoming persistent biochemical challenges, particularly in protein sample preparation, and highlights the value of such workflows when conventional sequence and structural data are unavailable.

## Results

### Characterization of an unknown co-eluting protein contaminant using electron microscopy

**Fig. 1:**
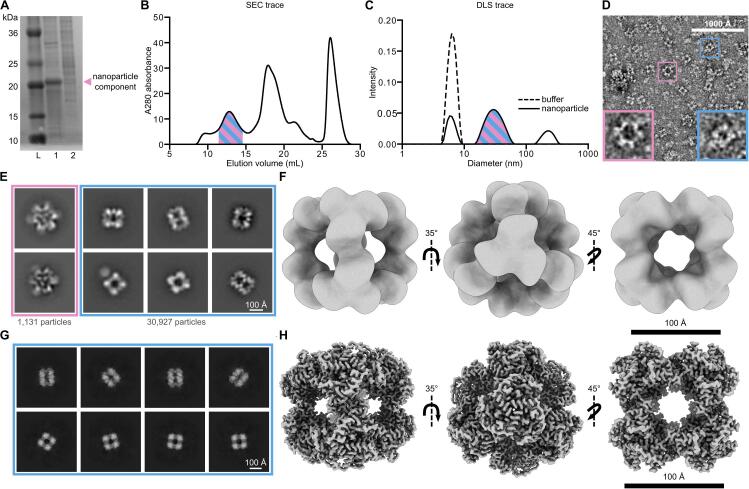
Characterization of an unknown co-eluting protein via electron microscopy. (A) SDS-PAGE of the IMAC eluate for the two designed protein components prior to mixing for assembly into the target designed octahedral nanoparticle. There is a clearly defined band in lane 1 for the one of the two components of the designed nanoparticle. The second component, shown in lane 2, is present in lower amounts and is less pure. (B) SEC trace of the assembled nanoparticle. The peak corresponding to the nanoparticle is highlighted in stripes to represent the co-elution of the contaminant with the on-target nanoparticle. (C) DLS trace of the SEC-purified sample highlighted in stripes to represent the contaminant and on-target nanoparticle diameters are not separated efficiently by DLS. The buffer (dotted line) was run as a control and contains detergent micelles found at 6.30 nm in diameter. (D) A portion of a ns-EM micrograph of the heterogeneous sample. The pink box represents the designed nanoparticle and the blue box represents the contaminant nanoparticle. (E) 2D ns-EM class averages of the on-target and contaminant nanoparticle species. A total of 1,131 on-target nanoparticles were processed and are represented here by two 2D class averages. 30,927 particles were processed for the contaminant species and are represented here by six 2D class averages. Corresponding particle numbers are reflective of all particles of each species in the dataset, not only those of the displayed classes. (F) 3D ns-EM map along the 2-, 3-, and 4-fold axes of symmetry of the contaminant nanoparticle generated using CryoSPARC v4.4. (G) 2D Cryo-EM class averages and (H) 3D Cryo-EM reconstruction viewed along the 2-, 3-, and 4-fold axes of symmetry of the contaminant nanoparticle using CryoSPARC v4.5. (Blue = contaminant protein; Pink = on-target two component nanoparticle). (For interpretation of the references to colour in this figure legend, the reader is referred to the web version of this article.)

During the assembly and purification of a novel two-component protein nanoparticle with designed octahedral symmetry (to be detailed in an upcoming publication), we discovered that a significant portion of the purified sample consisted of a smaller, unidentified protein complex, which also exhibited octahedral symmetry, as revealed by ns-EM **(**Fig. 1**)**. This smaller complex was also observed in various unrelated samples from the same laboratory, including samples of *de novo* designed fibrous proteins, cyclic oligomers, and two distinct icosahedral nanoparticles **(Sup.** Fig. S1**A-D)**. In each of these other instances, the unknown protein was low in concentration relative to the designed protein, and thus could be largely ignored during downstream characterization and analysis. For the designed octahedral two-component nanoparticle discussed here, the contaminant constituted the majority of the purified protein mass **(**Fig. 1**A-E, Sup.** Table S1**)**. This high level of contamination greatly hindered our ability to accurately assemble and characterize the designed nanoparticle in the quantity and purity necessary for effective downstream biochemical, biophysical, structural, and functional applications. To improve the purification of this two-component nanoparticle, identifying the contaminating protein and its source became essential.

Importantly, the intended designed two-component nanoparticle had a predicted mass of 1.18 MDa, a diameter of 29.2 nm, and required the presence of detergent for solubilization and purification. Our nanoparticle preparation protocol necessitated that the two protein components first be purified independently via immobilized metal-affinity chromatography (IMAC) (Bolanos-Garcia and Davies, 1760 (2006), Coskun, 2016, Bornhorst and Falke, 2000). The purified components were initially characterized via sodium dodecyl sulfate–polyacrylamide gel electrophoresis (SDS-PAGE) before being combined to form the desired two-component nanoparticle assembly. This assembly was then purified using size exclusion chromatography (SEC), resulting in a final estimated total protein yield of 97 μg **(Sup.** Table S1**)**.

The SEC trace of the putatively purified nanoparticle contained one relevant high molecular weight (MW) peak which eluted off the column between 10 mL and 15 mL, corresponding to a particle size greater than the 660 kDa MW standard for this column **(**Fig. 1**B)**. This peak fraction was analyzed using dynamic light scattering (DLS) to estimate the diameter of the purified nanoparticle (Stetefeld et al., 2016). To account for the presence of detergent micelles in the purification buffers, we performed DLS on the detergent-containing buffer used for SEC purification as a control, alongside the protein-containing peak isolated from SEC. The DLS results showed three peaks with average diameters of 6.30 nm, 30.9 nm, and 223 nm. The smallest (6.30 nm) peak was identified as containing detergent micelles, consistent with the DLS profile of the control. The central peak (30.9 nm) was identified as likely being the designed two-component nanoparticle, while the largest peak (223 nm) corresponded to protein and micellar aggregation **(**Fig. 1**C)**. Subsequent ns-EM analysis of the SEC-purified sample revealed the presence of two distinct nanoparticles with diameters (measured diagonally across the 4-fold axis) of approximately 18–––20 nm and 25–––30 nm **(**Fig. 1**D)**. The larger particle exhibited the predicted diameter and morphology for the designed two-component system, with spike-like proteins extending from an underlying octahedral nanoparticle scaffold **(**Fig. 1**E)**. However, the predominant species observed by ns-EM was a smaller, cube-like particle lacking the distinctive spiked features of our intended design **(**Fig. 1**E)**. As a result, the actual yield of the designed nanoparticle was estimated at approximately 3.79 µg from a total of 97 µg of protein purified via SEC **Sup.** Table S1**)**. Furthermore, subsequent ns-EM 3D refinements produced a map of this smaller nanoparticle that revealed an octahedral assembly profile which deviated significantly from our intended design **(**Fig. 1**F)**.

Given the prevalence of the smaller octahedral complex compared to our designed nanoparticle, and its unexpected presence across multiple unrelated samples, we turned to Cryo-EM SPA to investigate whether this complex might be another designed octahedral nanoparticle assembly, potentially resulting from cross-contamination due to the shared use of purification equipment. Following Cryo-EM data collection, initial 2D class averages of the smaller nanoparticle revealed clearly defined secondary structural elements **(**Fig. 1**G, Sup.** Fig. 2**A)**. Subsequent 3D refinement yielded a final map with a global resolution of 2.51 Å, with the local resolution ranging from 2.88 Å at the periphery to 2.44 Å in the protein core **(**Fig. 1**H, Sup.** Fig. 2**B-D, Sup.** Table S2**)** (Punjani et al., 2017). However, despite the high resolution of the map, the identity of the co-eluting nanoparticle remained unclear in the absence of known sequence or structural information.

**Fig. 2:**
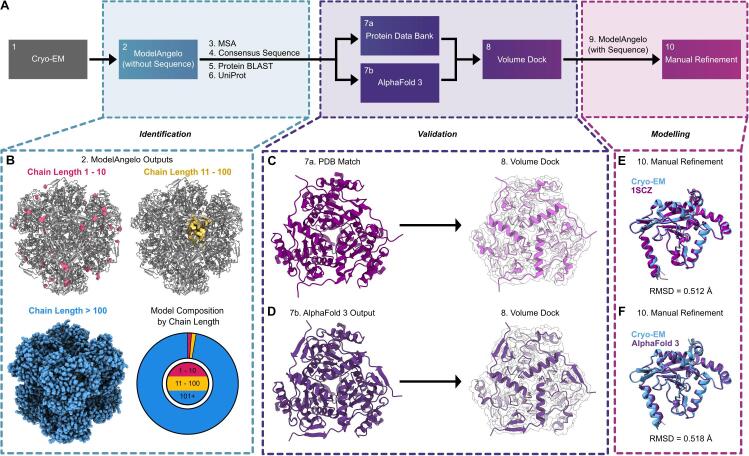
Structure-to-sequence workflow for the unambiguous identification of DLST. (A) An overview of the 10-step sequence-to-structure workflow used to identify DLST and build our atomic model used for structural analysis. (B) The sequence-agnostic ModelAngelo output using our 2.51 Å Cryo-EM map. Chains between 1 and 10 residues (pink), 11 to 100 residues (yellow), and longer than 100 residues (blue) are displayed in sphere view along the octahedral 3-fold axis of symmetry. An accompanying pie chart displays the percentage of all residues belonging to chains of those length ranges. (C-D) Published DLST crystal structure PDB:1SCZ (Schormann et al., 2004) (C) and AlphaFold 3 prediction model of DLST UniProt sequence A0A140NDX4 (Abramson et al., 2024) (D) each docked into the Cryo-EM density map of the unknown co-eluting protein. (E-F) A single subunit of our Cryo-EM model aligned to 1SCZ (RMSD 0.512 Å) (E) and the AlphaFold 3 model (RMSD 0.518 Å) (F). (For interpretation of the references to colour in this figure legend, the reader is referred to the web version of this article.)

### ModelAngelo structure-to-sequence identification of the contaminant protein

To identify the unknown nanoparticle in the absence of a corresponding amino acid sequence or prior structural information, we turned to the automated model-building software ModelAngelo **(**Fig. 2**A)** (Jamali et al., 2024). ModelAngelo’s primary mode uses deep learning to integrate available structural data and supplied sequence information to automatically build atomic models into Cryo-EM density maps (Jamali et al., 2024). It can alternatively operate in a sequence-agnostic manner, automatically generating and building polypeptide sequence fragments into well-resolved regions of Cryo-EM density maps when sequence information is unavailable. Running ModelAngelo in this manner on our 2.51 Å Cryo-EM map generated 92 all-atom sequence fragments of varying lengths, along with secondary structure predictions that were in strong agreement with the density of the unknown nanoparticle **(**Fig. 2**B)**. We aligned these output chain-fragments using ClustalOmega to generate a multiple sequence alignment which was then input into WebLogo, which produced a frequency plot displaying the relative abundance of amino acids at each position **(Sup.** Fig. S3**A)** (Sievers et al., 2011, Goldstein et al., 2015). From this frequency plot we constructed a consensus sequence using the most prevalent amino acid at each position **(Sup.** Fig. S3**B)**. This consensus sequence was input into Protein BLAST, which returned a list of potential matches for the unknown protein contaminant (Altschul et al., 1990). Remarkably, 98 of the top 100 Protein BLAST results identified dihydrolipoamide succinyltransferase (DLST) **(Sup.** Table S3**)**. DLST is an octahedral subunit of the E2 component of the ɑ-ketoglutarate dehydrogenase complex (KGDHC), which plays a pivotal role in the tricarboxylic acid (TCA) cycle, converting ɑ-ketoglutarate to succinyl-Coenzyme A and reducing nicotinamide adenine dinucleotide (Sheu and Blass, 1999, Knapp et al., 2000, Andi et al., 2019). The complex has three structured domains linked together by a chain of unstructured residues, with the C-terminal domain being the catalytic domain where multiple DLST subunits interact to form the full 24-subunit assembly (Andi et al., 2019). Notably, DLST has previously been observed as a contaminant protein in samples purified from *E. coli* expression systems (Bolanos-Garcia and Davies, 1760 (2006), Andi et al., 2019). Following the Protein BLAST result, a pairwise sequence alignment using EMBOSS Needle between the Uniprot sequence A0A140NDX4 of DLST from *E. coli* BL21 (DE3) and the ModelAngelo-derived consensus sequence was performed (Rice et al., 2000). This pairwise sequence alignment excluded the first 170 residues of DLST that were unresolved in our Cryo-EM map. The alignment revealed 60.3 % identity and 69.3 % similarity **(Sup.** Fig. S3**C)**, a strong indicator that the observed contaminant was DLST (UniProt Consortium, 2023, UniProt, (n.d.)).

**Fig. 3:**
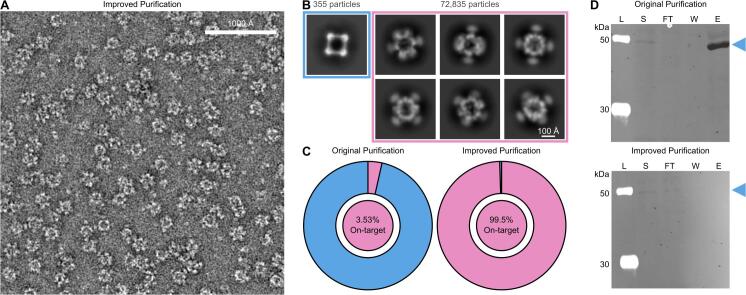
Modifications to the protein purification protocol result in an increased sample purity. (A) Representative ns-EM micrograph utilizing the optimized purification protocol. (B) Corresponding 2D ns-EM class averages of the on-target nanoparticle and DLST, with corresponding particle numbers listed for each. (C) Pie charts showing the relative abundance of the on-target nanoparticle and DLST as processed using the original purification protocol and the improved purification protocol. (Blue = contaminant protein, DLST; Pink = on-target two component nanoparticle). (D) Cropped anti-DLST Western Blot. IMAC soluble (S), flowthrough (FT), wash (W) and elution (E) fractions were run on SDS-PAGE followed by Western Blot. The LiCor Chameleon® 700 Pre-stained Protein Ladder (L) was used. Blue labels arrows indicate the molecular weight of a single subunit of DLST. A full annotated Western Blot as well as the accompanying SDS-PAGE gel can be referenced in Sup. Fig. 4 and Sup. Table S4. (For interpretation of the references to colour in this figure legend, the reader is referred to the web version of this article.)

To further validate the identity of the unknown protein as DLST, we next compared our Cryo-EM density map with previously published structures of the DLST catalytic domain, as well as AlphaFold 3 predicted structures of the UniProt DLST sequence **(**Fig. 2**C-D)** (Berman et al., 2000, Abramson et al., 2024). The highest resolution structure of DLST in the PDB (1SCZ; X-ray Crystallography; 2.20 Å) was selected for comparison and revealed significant agreement in secondary structure positions when docked into the Cryo-EM map **(**Fig. 2**C)** (Schormann et al., 2004). Similarly, AlphaFold 3 (AF3) predictions using the catalytic domain of the A0A140NDX4 UniProt sequence also showed high agreement with our Cryo-EM map **(**Fig. 2**D)** (UniProt, (n.d.), Abramson et al., 2024). A Western Blot was finally performed on all fractions collected during IMAC purification in an attempt to biochemically confirm that the contaminate was DLST **(Sup.** Fig. S4**, Sup.** Table S4**)**. Consistent with computational predictions, the anti-DLST Western blot revealed a prominent band near 50 kDa in the IMAC eluate, corresponding to the expected mass of a single DLST subunit **(Sup.** Fig. S4**)**. Together, the computational and experimental results unambiguously confirmed the identity of the co-eluting octahedral protein as DLST.

**Fig. 4:**
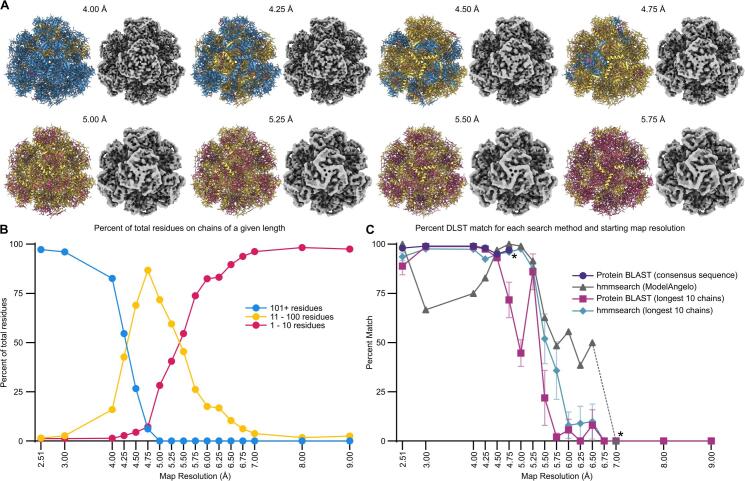
Impact of Cryo-EM resolution and search method on accurate DLST identification. The final Cryo-EM map was low-pass filtered to 3.00 Å, 8.00 Å, 9.00 Å, and each 0.25 Å interval between 4.00 Å and 7.00 Å. Each map was used as the input for the computational steps of our workflow, comparing the efficacy of three identification approaches: Protein Blast using a consensus sequence, Protein BLAST using the longest 10 chains, and hmmsearch using HMM profiles generated by ModelAngelo for the longest 10 chains. Chains with 1–10 residues are shown in pink, 11–100 residues in yellow, and chains longer than 100 residues in blue. (A) Low-pass filtered Cryo-EM maps and models between 4.00 Å and 6.75 Å. Additional maps and models spanning a broader resolution range can be found in Sup. Fig. S6. (B) Line graph illustrating the percentage of residues organized by chain length for each generated model. Raw data used for the line graph can be found in Sup. Table S5. (C) Line graph comparing the efficacy of the four identification methods across resolutions. Each data point represents the average percent identification accuracy for DLST using the specified method, with error bars representing the 95 % confidence interval for methods which had more than one data point per resolution. Asterisks (*) represent the lowest resolution data that returned any results for that search method. A dashed line marks the range between 6.50 Å and 7.00 Å on the hmmsearch (ModelAngelo) data to communicate that no results were returned for that method at 6.75 Å and consequently 6.50 Å is the lowest resolution that was able to achieve a non-zero score for that method. (For interpretation of the references to colour in this figure legend, the reader is referred to the web version of this article.)

To generate our final DLST catalytic domain model, the DLST sequence derived from *E. coli* BL21(DE3) was used as the input for another round of automated model building, this time utilizing the sequence-guided feature in ModelAngelo with the UniProt sequence for DLST **(**Fig. 2**A)** (UniProt, (n.d.)). The output from ModelAngelo was then further refined to produce the final model of the DLST catalytic domain **(Sup.** Fig. S3**D)**. Aligning this final model to both the 1SCZ monomer and AF3 outputs revealed RMSD values of 0.51 Å and 0.52 Å, respectively **(**Fig. 2**E-F)**.

### Revised purification methods increased purity of a de novo two component protein nanoparticle

Following the identification of the contaminant protein as DLST, we turned to the literature to explore potential strategies to exclude it from our future purifications. While previous studies noted DLST’s co-purification during IMAC of many proteins, none offered a rationale for its co-elution, nor did any outline an effective protocol to eliminate it from the purification pipeline (Bolanos-Garcia and Davies, 2006). In an effort to determine the underlying reason for DLST’s co-elution with multiple *de novo* protein samples, we first conducted a detailed analysis of its surface properties. Given the use of IMAC to purify our His-tagged designed two-component nanoparticle, we initially hypothesized that the surface histidine content of DLST might contribute to its binding during the purification process. Clusters of surface-exposed histidine residues could potentially interact with the nickel chelating resin in a manner similar to histidine tags on recombinant proteins, leading to its unintended co-purification. (Bolanos-Garcia and Davies, 1760 (2006), Coskun, 2016, Bornhorst and Falke, 2000). However, previous reports analyzing the surface histidine content of the DLST did not find any large histidine patches to support this as a potential mechanism for co-elution from IMAC columns (Bolanos-Garcia and Davies, 2006). Despite the known lack of histidine clusters, we considered that non-specific interactions between single histidines on the surface of DLST may have interacted with the IMAC column **(Sup.** Fig. S5**A)**. To test this, we applied an elution gradient ranging from 0 M to 0.5 M imidazole during IMAC purification, aiming to isolate the individual protein components and reduce DLST’s hypothesized weak binding to the resin. However, DLST was still detected in ns-EM samples after purification **(Sup.** Fig. S5**B-C).** Furthermore, in samples purified with an imidazole gradient, few to no on-target two-component nanoparticle assemblies were observed **(Sup.** Fig. S5**C, Sup.** Table S1**)**.

Given the numerous charged patches on the surface of DLST, we next tested the adjustment of sodium chloride (NaCl) concentrations in our buffers during cell lysis and IMAC purification to determine if increased ionic levels would selectively disrupt any non-specific ionic interactions occurring between DLST and the IMAC column **(Sup.** Fig. S5**D)** (Bornhorst and Falke, 2000). After separately purifying each protein component using IMAC with high salt buffers, followed by SEC purification of the nanoparticle, no DLST particles were detected in the sample by ns-EM **(Sup.** Fig. S5**E-F)**. However, while the higher NaCl concentrations eliminated DLST, it also significantly reduced the total yield of assembled on-target nanoparticles **(Sup.** Fig. S5**F, Sup.** Table S1**)**.

To exclude DLST while also preserving or improving the concentration of our on-target two-component nanoparticle, we next tested whether non-specific interactions between DLST and the on-target designed two-component nanoparticle were potentially occurring throughout purification. To do so, we opted to include excipients to all of our purification buffers—a common technique for purification of recombinant proteins to enhance yield and reduce protein aggregation (Ishihara and Hosono, 2015, Falconer et al., 2011, Jorgensen et al., 2009, Arakawa et al., 2007). In particular, amino acids such as glycine, threonine, arginine, glutamate, and histidine, have been successfully used as excipients to reduce non-specific protein–protein interactions, flocculation, and protein aggregation. They have also been shown to enhance protein stability in solution, particularly when working with computationally designed protein nanoparticles (Lutz et al., 2023, Khmelinskaia et al., 2023).

100 mM glycine and 100 mM arginine were added to all purification buffers to mitigate potential off-target interactions between our nanoparticle and DLST, which resulted in the near-complete removal of DLST in all ns-EM micrographs of these purified samples **(**Fig. 3**A)**. Strikingly, this approach significantly improved the final yield of our two-component nanoparticle assembly to an estimated 220 μg of total protein **(Sup.** Table S1**)**. ns-EM 2D class averages of over 70,000 particles revealed that the designed two-component nanoparticle now became the overwhelmingly dominant species, making up 99.5 % of extracted particles—a nearly 58-fold increase in on-target purified protein yield with excipients used throughout purification **(**Fig. 3**B-C, Sup.** Table S1**)**. In contrast, under standard purification conditions without excipients, the target nanoparticle accounted for only 3.53 % of extracted particles **(**Fig. 3**B-C, Sup.** Table S1**)**. Western Blot analyses comparing the purification of protein components with and without the use of excipients also indicated the near-complete removal from DLST in samples purified using 100 mM glycine and 100 mM arginine **(**Fig. 3**D, Sup.** Fig. S4**)**. To our knowledge, this is the first reported method for effectively removing DLST as a co-purifying protein contaminant during recombinant protein production from *E. coli*—a challenge that, if left unaddressed, could potentially hinder accurate downstream characterization of many natural and designed protein systems. This result underscores the crucial role excipients played in eliminating DLST from our purification process while significantly improving the overall yield of the on-target assembled material. Importantly, additional information pertaining to the design and characterization of our de novo designed two-component nanoparticle will be detailed in an upcoming manuscript.

### Effect of Cryo-EM resolution on accurate DLST identification and potential broader applicability

Following our successful exclusion of DLST, we aimed to explore the potential broader applicability of this ModelAngelo-to-BLAST identification approach, particularly under conditions where generating near-atomic resolution Cryo-EM data might not be feasible. Establishing the effectiveness of this workflow across a range of resolutions would increase its utility for various experimental setups, including those that often struggle to achieve high resolution. Specifically, we evaluated the ability of the pipeline to accurately identify DLST using Cryo-EM data spanning a broad resolution range by low-pass filtering the original 2.51 Å Cryo-EM map to resolutions between 3.00 Å and 9.00 Å, which were then used as inputs for the ModelAngelo-to-BLAST pipeline **(**Fig. 4**A, Sup.** Fig. S6**)**. The average length of the sequence fragments generated by ModelAngelo was inversely proportional to the map resolution, with lower resolution input maps yielding shorter length chain fragments **(**Fig. 4**B, Sup.** Table S6**)**. Following analysis of Protein BLAST results generated using the consensus sequences, the proportion of the top 100 results corresponding to DLST was plotted against their associated 3D map resolution values **(**Fig. 4**C,** Supplemental Sequences 1**, Sup.** Table S7–**12)** (Altschul et al., 1990). This analysis revealed that at resolution thresholds of 3.00 Å, 4.00 Å, 4.50 Å, and 4.75 Å, Protein BLAST accurately identified DLST as the most likely identity of the unknown protein in 99 %, 99 %, 98 %, 95 %, and 97 % of cases, respectively **(**Fig. 4**C)**. However, at 5.00 Å and at all lower resolutions, the consensus sequences did not yield any significant Protein BLAST results, indicating a lack of detectable sequence similarity among known proteins for sequence fragments generated by ModelAngelo at these resolutions **(**Fig. 4**C)**.

In addition to conducting Protein BLAST searches on consensus sequences, we also evaluated the ability of three other structure-agnostic approaches to identify DLST. These included the hmmsearch function built into ModelAngelo, external hmmsearch queries based on HMM profiles of the ten longest chains generated by ModelAngelo, and Protein BLAST searches using the same ten chains of each model (Jamali et al., 2024). For resolutions better than 4.50 Å, all three alternative methods demonstrated high identification rates. The latter two methods performed exceptionally well, achieving nearly 100 % success rates **(**Fig. 4**C)**. The hmmsearch function within ModelAngelo, however, exhibited a somewhat lower identification rate (∼70 %) within this range **(**Fig. 4**C)**. At lower resolutions, the performance of all methods declined, with external hmmsearch and Protein BLAST each using the ten longest chains correctly identifying DLST as the contaminant in fewer than 20 % of cases by 6.00 Å **(**Fig. 4**C)**. The hmmsearch function within ModelAngelo maintained a higher identification rate (∼50 %) at resolutions as low as 6.50 Å, outperforming the other methods in this range **(**Fig. 4**C)**. Ultimately, none of the tested methods were successful at identifying DLST at resolutions worse than 6.50 Å **(**Fig. 4**C)**. These findings suggest that combining methods can optimize the identification of unknown proteins in Cryo-EM samples, with the choice of method tailored to the resolution of the starting structure. The flexibility of these four methods across a wide resolution range underscores their broad applicability in addressing diverse biological and experimental challenges in protein identification, even when near-atomic resolution cryo-EM data is unavailable.

## Discussion

Addressing the challenge of co-eluting protein contaminants during protein purification is essential for the production and accurate experimental characterization of recombinant proteins. In the case of the designed octahedral two-component nanoparticle system discussed here, the presence of a co-purifying protein artificially inflated the estimated concentration of the designed components required for nanoparticle assembly, leading to inconsistent assembly conditions and inaccurate estimations of the final on-target nanoparticle concentration **(**Fig. 1**, Sup.** Table S1**)**. Thus, it became necessary to identify and exclude this protein from our purifications so that we could more effectively produce and characterize our on-target design. By using the ModelAngelo-to-BLAST pipeline and cross-referencing our Cryo-EM data with experimentally determined PDB structures, AlphaFold 3 predictions, and traditional biochemical analysis, we unambiguously identified the co-purifying protein as DLST and optimized our purification protocols to eliminate it.

Before obtaining secondary structure information from Cryo-EM and implementing the ModelAngelo-to-BLAST pipeline, we hypothesized that the co-eluting protein might be the result of cross-sample contamination from another computationally designed protein purification. During our investigation, we explored several alternative methods for determining the identity of the unknown protein. One study utilizing Cryo-EM had successfully identified a number of unknown proteins found in native cell extracts utilizing the software package Omokage (Suzuki et al., 2016). However, this approach requires existing structural information to confidently identify the unknown protein species (Suzuki et al., 2016, Skalidis et al., 2022). Furthermore, a retrospective analysis of our data revealed that Omokage misidentified the contaminant protein in most cases, proposing potential identifications that included both computationally designed and natural proteins, though DLST also appeared among these candidates. These misidentifications underscore the lower success rate of this approach compared to the more accurate sequence-based methods benchmarked in this study.

We also considered traditional experimental methods for our initial identification of the contaminant, such as liquid chromatography-tandem mass spectrometry (LC-MS/MS) and electrospray ionization mass spectrometry (ESI-MS). While these techniques are highly valuable, they posed significant anticipated challenges for our system, primarily due to our reliance on detergents to stabilize our designed two-component nanoparticle. Indeed, detergents are well-known to complicate mass spectrometry workflows and the interpretation of final data, adding significant complexity to an already complicated purification process (Loo et al., 2003). Unlike the Omokage and mass spectrometry methods, the ModelAngelo-to-BLAST pipeline did not rely on prior PDB structural information, and enabled us to accurately identify the contaminant using only our experimental Cryo-EM data and publically available sequence databases. Further validation using AlphaFold 3 models was as robust as PDB models during docking into the Cryo-EM density map, demonstrating that ModelAngelo-to-BLAST identifications can be verified even without the availability of pre-existing PDB information or manual building (Abramson et al., 2024, Skalidis et al., 2022). Additionally, while Western blotting was used to biochemically confirm the computational predictions, its success was heavily contingent upon the computational results correctly guiding the selection of the appropriate primary antibody. Thus, the Western blot served primarily as a validation tool, reinforcing the reliability of the computational pipeline used here.

An advantage of this computational approach to contaminant identification is its flexibility across a wide range of resolutions. While we initially relied on a near-atomic resolution Cryo-EM map for the ModelAngelo-to-BLAST pipeline, we found that significantly lower resolutions were still sufficient to accurately identify DLST in our sample, consistent with previous benchmarking results for ModelAngelo **(**Fig. 4**C, Sup.** Table S3**)** (Altschul et al., 1990). This robustness across resolutions highlights the adaptability of the pipeline, making it particularly valuable for experimental setups where achieving near-atomic resolution data is not feasible due to sample heterogeneity, limited imaging time, or other technical constraints.

Our analysis further revealed that a tailored combination of methods provided the highest level of confidence across varying resolutions. Notably, Protein BLAST using consensus sequences excelled at resolutions better than 5.00 Å for correctly identifying DLST. In contrast, the hmmsearch function within ModelAngelo was most reliable within the 5.00 Å to 6.50 Å resolution range, but somewhat unexpectedly, showed reduced reliability at higher resolutions. This diminished performance was due to the hmmsearch function frequently misidentifying the contaminant as dihydrolipoamide acetyltransferase (DLAT) at resolutions between 2.51 Å and 5.0 Å. This issue highlighted the importance of leveraging multiple complementary approaches, as each method demonstrated strengths and weaknesses for this sample depending on the resolution range. Notably, methods that analyzed the longest chains of each DLST model—either through Protein BLAST or hmmsearch—offered minimal to no additional practical benefit across any resolution range compared to the other two approaches, while also requiring significantly more time and effort to prepare.

The ability of these pipelines to function effectively across such a broad range of resolutions demonstrates their potential as practical tools for addressing contaminant identification challenges. This is particularly relevant for diagnosing sample purity issues in non-routine purifications or experimental scenarios where cryo-EM data collection is feasible or preferred for biochemical or experimental reasons, yet achieving near-atomic resolution may not be practical.

Regarding the high-resolution Cryo-EM structure of the DLST catalytic domain determined in this study, we note that minimal differences were observed between our structure and other previously reported crystallographic and Cryo-EM structures of the DLST catalytic domain **(Sup.** Fig. S7**)**. The resolution we achieved for DLST using octahedral refinement allowed for the clear construction of a detailed intramolecular water network, which was particularly well-resolved at the catalytic pocket **(Sup.** Fig. S7**A-D)**. Comparison of this water network with the highest resolution published structure revealed key differences in the assignment of H_2_O atomic coordinates **(Sup.** Fig. S7**E-F)** (Schormann et al., 2004). These differences may reflect differences in the protein crystallization conditions versus the native-like environment of Cryo-EM sample preparation, potentially offering valuable insights for future studies aimed at understanding this enzyme's mechanistic activity. Additionally, asymmetric data processing of DLST reduced both local and global resolution of the catalytic domain but uncovered an intriguing, previously unobserved segment of density along one of the 4-fold axes of DLST **(Sup.** Fig. S8**)**. This unidentified density, which globally resolves to between 5.50 Å and 8.50 Å, measures approximately 20 Å along its longest axis, and is positioned proximally to DLST residues E224, I229, R230, K299, V303, R306, and D307 **(Sup.** Fig. S8**)**. Notably, the portion of this density that resolves to better than 6.50 Å—the lowest resolution threshold at which we identified DLST (a significantly larger protein)—is extremely small. This limited resolution poses challenges to its identification, with further attempts to characterize this density falling beyond the scope of this study. Despite this, we believe these findings are important and provide a foundation for future studies aimed at elucidating the structure, dynamics, and function of this enzyme in greater detail.

Ultimately, by leveraging advanced tools such as Cryo-EM, ModelAngelo, Protein BLAST, the PDB, and AlphaFold 3, we transformed a challenging purification problem into an opportunity to refine our techniques and enhance the quality of our results. This integrated approach underscores the potential of combining Cryo-EM with emerging AI and sequence-based tools as robust alternatives for tackling long-standing biochemical challenges—particularly in purifying proteins from complex biological systems, even when near-atomic resolution data cannot be achieved.

## Materials and methods

### Protein purification of the two-component octahedral nanoparticle

The target two-component octahedral nanoparticle was designed *in silico* using a *de novo* designed tetramer and a soluble trimer based on the aldolase 2-keto-3-deoxy-6-phosphogluconate (KDPG). Specifics on the components for this designed system will be discussed further in a separate manuscript detailing this design. Thousands of octahedral nanoparticle designs were screened *in silico* through careful evaluation of the interface residues. Filters were applied to all designs during *in silico* screening until the top 10 nanoparticles were chosen to be evaluated *in vitro*. For the top designs, C-terminal histidine-tagged plasmids were ordered from GenScript and transformed into *E. coli* BL21 DE3 competent cells.

For the designed KDPG trimer, *E. coli* BL21(DE3) cells (NEB, Cat. C2527H) were transformed with the trimer plasmid. A colony was picked and cultures were grown in Terrific Broth media at 37.0 °C until an OD600 of 0.60–––0.80 before induction with a final concentration of 1 mM Isopropyl β- d-1-thiogalactopyranoside (IPTG) and grown for an additional 3 h at 37.0 °C. Cells were harvested at 5000 revolutions per minute (rpm) for 15 min and resuspended with the lysis buffer (25.0 mM Tris-HCl pH 8.00, 0.15 M NaCl, 100 mM arginine, 100 mM glycine, 1.00 mg/mL lysozyme and 1.00 mg/mL DNAse) then lysed using sonication. Lysed cell resuspensions were sonicated for a total of 5 min using 10.0 s sonication pulses and 10.0 s breaks. After cell lysis, the solution was pelleted at 10,000*g* for 30 min. The supernatant was collected and ran through a nickel-nitrilotriacetic acid (Ni-NTA) IMAC resin with a bed volume of 5.00 mL (Qiagen, Cat. 30250). The IMAC resin was equilibrated with 5.00 column volumes of loading buffer (25.0 mM Tris-HCl pH 8.00, 0.15 M NaCl, 100 mM arginine and 100 mM glycine) before the addition of cell lysate, then washed with 3 column volumes of the loading buffer. The bound protein was eluted with 1.50 column volumes of elution buffer (25.0 mM Tris-HCl pH 8.00, 0.15 mM KCl, 0.50 M imidazole, 0.15 % n-Dodecyl-B-D-maltoside (DDM), 100 mM arginine and 100 mM glycine).

For the tetrameric protein component, plasmids were transformed into BL21 Star (DE3)pLysS One Shot competent cells (Promega, Cat. L1195). Cells were grown for 3 h at 37.0 °C until an OD600 of 0.80–––1.00 before induction with a final concentration of 1 mM Isopropyl β- d-1-thiogalactopyranoside (IPTG) and grown for an additional 18 h at 18.0 °C. The cells were then lysed in the same fashion as described above. Following lysis by sonication, cells expressing the tetramer were spun at 10,000 *g* for 15 min and the supernatant was collected for ultracentrifugation at 130,000 *g* for 1.00 h at 4.0 °C. A resuspension buffer (25.0 mM Tris-HCl pH 8.00, 0.15 M KCl, 100 mM arginine, 100 mM glycine and 2.00 % DDM) was used to resuspend the pellet after ultracentrifugation. This resuspension was placed on a rocker at 4.0 °C overnight. The following day, the resuspended solution was spun for an additional 30 min at 14,000 *g*. The supernatant was collected and ran through an Ni-NTA IMAC resin with a bed volume of 5.00 mL. The IMAC resin was equilibrated with 5.00 column volumes of resuspension buffer before the addition of cell lysate, then washed with 3 column volumes of the resuspension buffer and eluted with 1.50 column volumes of elution buffer (25.0 mM Tris-HCl pH 8.00, 0.15 mM KCl, 0.50 M imidazole, 0.15 % n-Dodecyl-B-D-maltoside (DDM), 100 mM arginine and 100 mM glycine).

Purified proteins were mixed together at a 1:1 (c/c) by molar ratio for 1 h at 4.0 °C to facilitate nanoparticle assembly. Further purification of the nanoparticles was achieved using size exclusion chromatography (SEC) in an SEC buffer (25.0 mM Tris-HCl pH 8.00, 0.15 mM NaCl, 0.10 % DDM, 100 mM arginine and 100 mM glycine) on a Cytiva Superose® 6 Increase 10/300 GL column. The SEC column was calibrated using the Cytiva Gel Filtration HMW Calibration Kit (Cat. 28403842) with molecular masses ranging from 43,000 to 669,000 Daltons. The nanoparticle peak purified on the SEC was isolated and characterized by DLS, ns-EM and Cryo-EM.

All lysis, resuspension and purification buffers resulting in the co-purification of DLST with the on-target nanoparticle did not contain 100 mM glycine and 100 mM arginine. Otherwise, the purification protocols described here are the same for samples where co-purification of DLST with our two-component nanoparticle were observed.

### Western Blot of IMAC-purified components

After each step of Ni-NTA purification of the protein nanoparticle components with and without the use of excipients, 500 µL of sample was saved for SDS-PAGE gel on a Criterion™ TGX Stain-Free™ Precast Any kD gel (Cat. 5678125). Soluble protein lysate and NTA flowthrough fractions were diluted to prevent overloading the sample signal on the gel. The NTA Wash and Elution fractions of the protein components were run on the gel without dilution. 10 µL of each sample was mixed with 10 µL of 2x Laemmli Sample Buffer (Brio-Rad, Cat. 1610737) and boiled for 10 min at 95 °C. The gel was loaded with 10 µL Bio-Rad Precision Plus Protein Unstained Standards (Bio-Rad, Cat. 1610363) ladder for SDS-PAGE applications, 4 µL of LiCor Chameleon® 700 Pre-stained Protein Ladder (LiCor, Cat. 928–70000) for Western Blot applications, and 10 µL of each boiled sample.

A Western Blot was performed following stain-free imaging of the SDS-PAGE gel. The gel was transferred onto a 0.2 µm Nitrocellulose membrane (Bio-Rad, Cat. 1620112) and blocked for 1 h using a 1 % solution of Blocking Buffer (1 % w/v Blocking-Grade Blocker (Bio-Rad, Cat. 1706404) dissolved in 25 mM Tris-HCl pH 8.0, 150 mM NaCl and 0.05 % Tween-20 (TBS-T)). A polyclonal C-terminal DLST antibody (Antibodies Online, Cat. ABIN2788818) was used as the primary antibody to detect DLST in the samples. The primary antibody was incubated with the membrane at a 1:100 dilution in the blocking buffer for 1 h. The membrane was washed three times with TBS-T and 25 mL of new blocking buffer was added with a 1:1000 dilution of the secondary antibody Goat anti-Rabbit IgG FITC (Antibodies Online, Cat. ABIN101988) to visualize DLST. Western Blots were imaged on the LiCor Odyssey® M using the 700 nm wavelength channel to visualize the ladder and 488 nm channel to detect the FITC fluorescence signal.

### ns-EM sample preparation

After SEC, the purified peak was diluted from 0.12 mg/mL to 0.10 mg/mL using the SEC buffer. 3 µL of the diluted sample was applied to a freshly glow discharged 400 carbon square mesh grid (Electron Microscopy Sciences, EMS) and allowed to adsorb onto the grid for 30 s. Blotting paper (Wattman) was used to remove excess solution from the grid before the application of 3 µL 2 % uranyl formate (2UF) to stain the grid for ns-EM. Excess stain solution was blotted immediately followed by another application of 3 µL 2UF. This was repeated once more, resulting in a total of 3 applications of 2UF. Following the final application of 2UF, all remaining solution was blotted and the sample was allowed to dry completely. There was no waiting period in between each application of 2UF, and after each blot new 2UF was applied to the grid immediately.

### ns-EM data acquisition and processing

Negative stain electron microscopy (ns-EM) micrographs were collected on a Thermo Fisher Scientific Talos L120C 120 kV transmission electron microscope with a LaB_6_ filament and CETA camera with a pixel size of 2.49 Å at 57,000x magnification. 164 micrographs were recorded at 57,000x magnification at a total dose rate 40.8 e^-^/Å^2^ for the octahedral nanoparticle. Micrographs were imported into CryoSPARC v4.4 (Punjani et al., 2017) and Patch CTF Estimation was performed prior to blob picking, inspection, and extraction of 22,158 picks with a box size of 180 pixels (448.2 Å). With CTF correction off, picks were classified into 50 2D classes, four of which were selected to serve as templates for an additional round of particle picking. In this second round of particle picking, picks were inspected and extracted to 180 pixels (448.2 Å) before classification of 55,802 extracted particles into 150 2D classes with CTF correction off. 5 out of 150 of these 2D classes were selected as a second round of templates for another round of particle picking and extraction. In this last round, 49,262 particles were extracted to 180 pixels (448.2 Å) using the templates from the second round of extraction and classified into 50 2D classes with a batch size of 400 particles and CTF correction off. 30,927 particles were used to homogeneously refine a map into Octahedral symmetry with a maximum resolution threshold of 20 Å. Particles were aligned into three 3D octahedral ab-initio reconstruction classes. For subsequent ns-EM data processing for DLST, we followed a similar pipeline outlined here.

### Cryo-EM sample preparation

Prior to freezing, 3 µL of protein sample, at an estimated final concentration of 0.3 mg/mL, was applied to glow-discharged Quantifoil 2/2 holey carbon grids overlaid with a thin layer of additional carbon. Vitrification was performed using a Vitrobot MkIV at 22 °C and 100 % humidity, with a wait time of 7.5 s, a blot time of 0.5 s, and a blot force of 0, before being immediately plunge-frozen into liquid ethane. The sample grids were clipped following standard protocols before being loaded into a 300 kV Titan Krios for imaging.

### Cryo-EM data acquisition and processing

A total of 6,211 movies were collected using SerialEM (Mastronarde, 2003, Mastronarde, 2005) on a 300 kV FEI Titan Krios equipped with a Gatan K3 direct electron detector and a BioQuantum Gif energy filter. The movies were recorded at 105,000x magnification, with a pixel size of 0.843 Å/pixel. Each movie consisted of 99 frames recorded at a frame rate of 19.8 frames per second, with a dose rate of 9.4 e-/Å^2^/s and an exposure time of 5 s, resulting in a total exposure dose of 47.0 e-/Å^2^. All movies were imported into CryoSPARC v4.4 for data processing (Punjani et al., 2017). Initially, the movies were corrected for Patch Motion Correction. Defocus and astigmatism values were estimated using the Patch CTF with the default parameters. After two rounds of exposure curation, 4,264 exposures were selected for particle picking. Prospective particles were identified using blob picking with a diameter range of 200 to 400 Å, corresponding to the expected sizes of both our intended and co-purified proteins. This process identified 808,774 prospective particles, which were then inspected and curated based on power histogram values and normalized cross-correlation scores. Following this curation, 456,319 particles were extracted at a box size of 600 pixels. These particles were classified into 150 classes with a batch size of 400 per class. 4 classes were selected to serve as templates for another round of particle picking with particle diameter set to 300 Å. This round yielded 1,468,115 prospective particles, which were curated down to 765,699 particles. These were extracted at 600 pixels and classified into 150 classes again. All high-quality particle classes (47,980 particles) were re-extracted with recentering enabled, resulting in 47,480 particles, which were then split into 150 classes, yielding 39,222 high-quality particles. Of these, 19,033 of the unknown protein contaminant particles were reconstructed into 3 ab-initio 3D C1 reconstruction classes. All particles were homogeneously refined into the best Ab-initio volume with octahedral symmetry enabled, achieving a resolution of 2.62 Å. Local CTF refinement was performed, followed by another round of homogeneous refinement, improving the resolution to 2.59 Å. Reference Based Motion Correction was then performed which reduced the number of particles to 18,809. Subsequently, Global CTF refinement was run for two iterations with Fit Anisotropic Mag set to true. One final homogenous refinement was run in Octahedral symmetry with Minimize over per-particle scale and Optimize per-group CTF parameters set to true, yielding a map with a final estimated resolution of 2.51 Å. This map was then sharpened using deepEMhancer on the “highRes” model and filtered to 2.51 Å. All other parameters were left as default.

### Asymmetric refinement of the catalytic domain

To produce an asymmetric model of the catalytic domain, best ab-initio volume was also homogeneously refined using the same 19,033 particles used for octahedral refinement, but without a symmetry operator enforced. The 3.79 Å homogenous refinement was sharpened using DeepEMhancer to produce our final asymmetric map.

### Initial identification of co-purified protein contaminant from octahedral nanoparticle samples

ModelAngelo was run on the final Cryo-EM map, sharpened using deepEMhancer, in a sequence-agnostic manner to generate sequence segments fit to the density as a cif file (Eddy and hmmer, 2007). PyMol (n.d.) was used to generate a FASTA sequence (Goldstein et al., 2015) from the cif file which was aligned by the multiple sequence alignment program Clustal Omega (Sievers et al., 2011). The aligned sequences were then exported and used by sequenced logo program WebLogo (Crooks et al., 2004) to generate a frequency plot of the aligned sequences. The most frequent amino acid present at each residue was used to create a consensus sequence. This sequence was input into the National Institutes of Health (NIH)’s Protein BLAST (Altschul et al., 1990) and the result strongly indicated that the most likely candidate protein was dihydrolipoamide succinyltransferase **(Sup.** Table S3**)**. PDB assembly 1SCZ (Schormann et al., 2004) of the catalytic domain of dihydrolipoamide succinyltransferase was aligned to our Cryo-EM volume and used to visually confirm the identity of the contaminant.

### Generation of low-pass filtered maps for resolution based analysis

To generate maps of the contaminant with resolutions ranging from 3.00 to 9.00 Å, the 2.51 Å Cryo-EM map (prior to sharpening with deepEMhancer) was low-pass filtered using the Volume Tools job in CryoSPARC to 3.00 Å, 8.00 Å, 9.00 Å, and each 0.25 Å interval between 4.00 Å and 7.00 Å. The maps output by these jobs were then used as inputs for ModelAngelo without an input sequence to produce models containing chain fragments and corresponding HMM profiles, and from which consensus sequences could be generated using the protocol designed for the 2.51 Å map. Protein BLAST analysis of the consensus sequences for each model.

Each consensus sequence was individually used as the input for a Protein BLAST search on the non-redundant protein sequences (nr) database with default parameters and with no taxonomic restriction applied (Altschul et al., 1990). The descriptions table for each result was downloaded and trimmed to the 100 results with the lowest E value. The protein names (from the descriptions column) were then searched for one of the key terms identified for unambiguous DLST identification. All results were formatted in lowercase, then screened to identify the presence of any of the following terms: “dihydrolipoamide succinyltransferase”, “dihydrolipoyllysine-residue succinyltransferase”, “dihydrolipoyltranssuccinylase”, “dihydrolipoyltranssuccinate transferase”, or “dihydrolipoyltranssuccinase”. To prevent potential ambiguity or mis-identification in results, terms such as “2-oxo acid dehydrogenase subunit E2” were not considered matches unless they also contained one of the above terms. A percent DLST match score was assigned to each chain from each method representing the number of the top 100 results that could be unambiguously identified as DLST. For results which contained fewer than 100 entries, the percent DLST match was determined based on all of the entries available. For example, the Protein BLAST result for chain 2 of the 5.50 Å model was assigned a score of 93.3 as 14 of the 15 entries returned matched DLST **(Sup.** Table S3**)**. Because results with no entries would have an indivisible score (as the non-existent matching entries would have to be divided by zero total entries), those results are scored with a double dash (−-), and were excluded from Fig. 4**C (Sup.** Table S3**)**.

### Protein BLAST analysis for the longest 10 chains of each model

The FASTA sequence of each model output by ModelAngelo was sorted by chain length, and the sequences of the longest 10 chains were recorded for subsequent analysis. Each sequence was then used as the input for a novel Protein BLAST search and assigned a subsequent DLST match score using the same methods and parameters that were used for the consensus sequences **(Sup.** Table S3**)**.

### Hmmsearch analysis (in ModelAngelo) for each model

The hmmsearch function within ModelAngelo (Jamali et al., 2024) was executed on each model referencing *E. coli (BL21 DE3)* reference proteome UP000002032 (UniProt Consortium, 2023). The all_hits.csv results were − like with searches using Protein BLAST − trimmed to the 100 entries with the lowest E value, or all entries for results with less than 100 entries and DLST match scores were assigned using the same criteria as for Protein BLAST.

### Hmmsearch analysis (in HMMER 3.4) for the longest 10 chains of each model

Using the chain names of the longest 10 chains for each model identified during Protein BLAST analysis, the corresponding HMM profiles generated by ModelAngelo for the longest 10 chains of each model were identified for use with hmmsearch directly through HMMER 3.4 against sequence database UniProtKB/TrEMBL (UniProt Consortium, 2023, Eddy and hmmer, 2007). The output files were then processed in the same manner as the Protein BLAST and ModelAngelo hmmsearch outputs.

### Cryo-EM structural refinement

Following identification of the contaminant protein as DLST, the *dlst* gene sequence from BL21 DE3 *E. coli* was pulled from UniProt (entry A0A140NDX4) (UniProt Consortium, 2023, UniProt, (n.d.)) and used as the input sequence for automated model building using ModelAngelo along with the 2.51 Å deepEMhancer (Sanchez-Garcia et al., 2021) sharpened volume map. The resulting model was imported into PyMOL (PyMOL, n.d.) to extract its FASTA (Goldstein et al., 2015) sequence, the chains of which were then aligned with the UniProt gene sequence once more to verify that deviations to the sequence were not introduced. Then the model was relaxed using ISOLDE (Croll, 2018) in ChimeraX (Pettersen et al., 2021, Meng et al., 2023, Goddard et al., 2018) to ensure the model fit moderately well to the density. Subsequently, one chain of the model was isolated and retained while all other copies were deleted. Symmetry operators were applied to the remaining chain in ChimeraX (Pettersen et al., 2021, Meng et al., 2023, Goddard et al., 2018) to repopulate the density with the 23 additional subunits. The model was then iteratively processed using Coot (Emsley et al., 2010), Phenix (Liebschner et al., 2019, Davis et al., 2007), and ISOLDE (Croll, 2018) to accurately orient the backbone and sidechains. After each iteration of processing, the A chain was preserved and other chains were first deleted, and then repopulated using ChimeraX (Pettersen et al., 2021, Meng et al., 2023, Goddard et al., 2018) symmetry operators. Periodically, between iterations, the model was submitted to the wwPDB validation service (wwPDB consortium, 2019) to provide an additional metric of model quality. Once the iteratively produced model was deemed by the authors to be of sufficient quality, a mask was generated in ChimeraX (Pettersen et al., 2021, Meng et al., 2023, Goddard et al., 2018) of the 2.51 Å map sharpened (without using deepEMhancer (Sanchez-Garcia et al., 2021) preserving only regions within 6 Å of chain A. A network of waters with density present in this mask was built in Coot (Emsley et al., 2010) and then symmetrized over the whole model in ChimeraX (Pettersen et al., 2021, Meng et al., 2023, Goddard et al., 2018). The symmetrized model was examined in ChimeraX and Coot to identify regions where waters were duplicated (as waters were built along all interface betweens chain A and the adjacent chains), all but one copy of each of these waters were deleted such that following an additional round of symmetrization the final model possessed no duplicates. The full model was refined one last time in Phenix (Liebschner et al., 2019) using the 2.51 Å map sharpened (without using deepEMhancer) map. The distance between each water and nearby non-Hydrogen atoms was measured to verify that the waters were not placed too near or far from the model. The model was manually inspected in Chimera (Pettersen et al., 2004) as a final quality check and then the final model was once again submitted to the wwPDB validation service (wwPDB consortium, 2019) to provide a quantitative assessment of model quality prior to deposition. The final structure was deposited in the Protein Data Bank (PDB) and Electron Microscopy Data Bank (EMDB) under accession numbers 9DZ8 and EMD-47326 respectively.

### Funding

This work was supported by: The Audacious Project at the Institute for Protein Design (K.D.C., D.Z., A.G., H.P., N.P.K., A.J.B.), the Bill and Melinda Gates Foundation (INV-043758, INV-010680; K.D.C., C.W., A.G., N.P.K., A.J.B.), the Open Philanthropy Project Improving Protein Design Fund (K.D.C), Washington Research Foundation Innovation Fellows Program (A.C.), and the National Science Foundation under Grant TI-2229291 (N.P.K.).

### CRediT authorship contribution statement

**Kenneth D. Carr:** Writing – review & editing, Writing – original draft, Visualization, Validation, Methodology, Investigation, Formal analysis, Data curation, Conceptualization. **Dane Evan D. Zambrano:** Writing – review & editing, Writing – original draft, Visualization, Validation, Methodology, Investigation, Formal analysis, Data curation, Conceptualization. **Connor Weidle:** Writing – review & editing, Writing – original draft, Visualization, Validation, Investigation, Formal analysis. **Alex Goodson:** Writing – review & editing, Visualization, Data curation. **Helen E. Eisenach:** Writing – review & editing, Visualization, Data curation. **Harley Pyles:** Writing – review & editing, Visualization, Data curation. **Alexis Courbet:** Writing – review & editing, Visualization, Data curation. **Neil P. King:** Writing – review & editing, Supervision, Resources, Methodology, Funding acquisition. **Andrew J. Borst:** Writing – review & editing, Writing – original draft, Visualization, Validation, Supervision, Resources, Project administration, Methodology, Investigation, Data curation, Conceptualization.

## Declaration of competing interest

The authors declare that they have no known competing financial interests or personal relationships that could have appeared to influence the work reported in this paper.

## Data Availability

Data will be made available on request.
