# Supplementary material for: Protein identification using Cryo-EM and artificial intelligence guides improved sample purification
Source: J Struct Biol X. 2025 Jan 21;11:100120. doi: 10.1016/j.yjsbx.2025.100120 (PMC11830286; doi:10.1016/j.yjsbx.2025.100120)
Supplement: Supplementary Data 1 [file mmc1.docx]

Protein identification using Cryo-EM and artificial intelligence guides improved sample purification [Supplement]

Kenneth D. Carr*^1,2^, Dane Evan D. Zambrano*^1,2^, Connor Weidle^1,2^, Alex Goodson^1,2^, Helen E. Eisenach^1,2^, Harley Pyles^1,2^, Alexis Courbet^1,2^, Neil P. King^1,2^, Andrew J. Borst^†1,2^

* Contributed equally

† Corresponding author

1. Department of Biochemistry, University of Washington, Seattle, WA 98195, USA

2. Institute for Protein Design, University of Washington, Seattle, WA 98195, USA


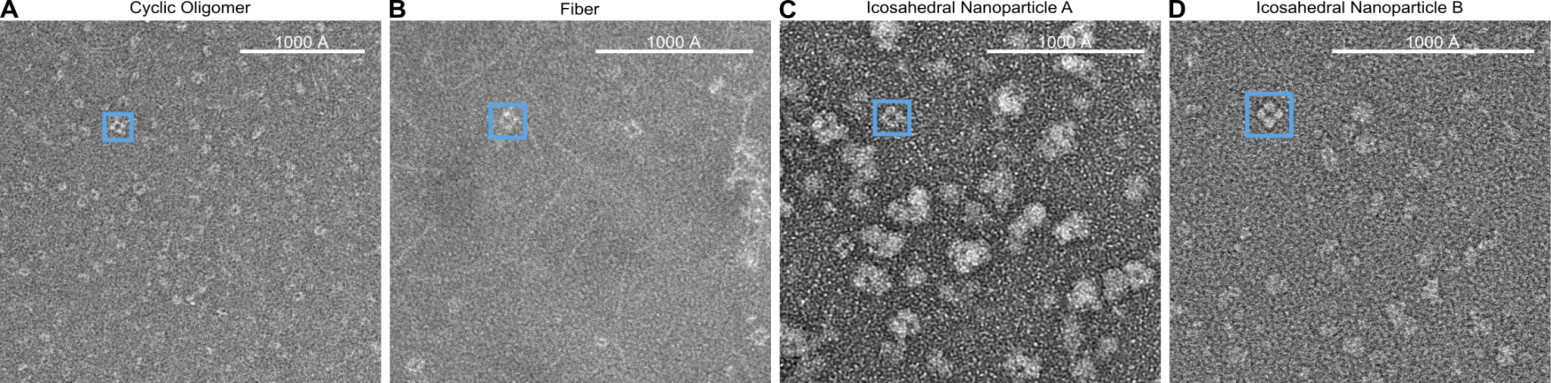


## Supplemental Figure 1. Octahedral contaminant protein observed in additional de novo protein samples by negative stain electron microscopy. Sections of representative ns-EM micrographs demonstrating observation of the octahedral contaminant protein in multiple samples of de novo designed proteins imaged at various magnifications, with 1000 Å scale bars sized proportionally. (A) A sample of a de novo cyclic-oligomeric protein imaged at 45,000x magnification. (B) A sample of a de novo protein fiber imaged at 57,000x magnification. (C) A sample of an icosahedral nanoparticle imaged at 57,000x magnification. (D) A sample of a different de novo icosahedral nanoparticle imaged at 73,000x magnification.

##
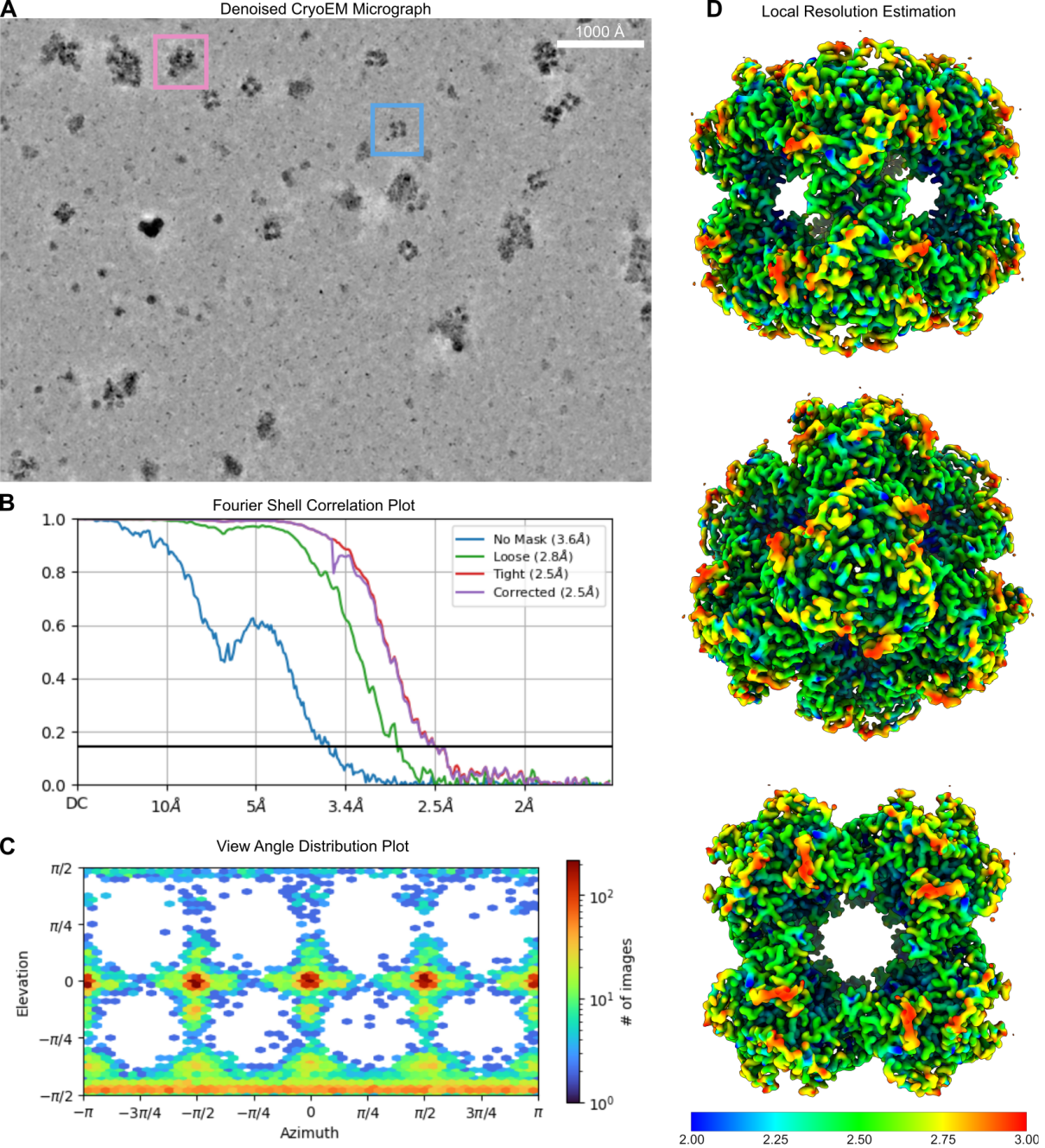


## Supplemental Figure 2. Cryo-EM Data processing metrics. (A) Denoised representative Cryo-EM micrograph at 105,000x magnification showing particles of both the designed two-component nanoparticle and DLST. (Blue = contaminant protein; Pink = on-target designed two-component nanoparticle). (B) Fourier shell correlation (FSC) plot of the Cryo-EM map. (C) View angle distribution plot of the Cryo-EM map. (D) Local resolution estimate of the Cryo-EM map with a 0.143 FSC cutoff. Colors range from blue at 2.00 Å to red at 3.00 Å.


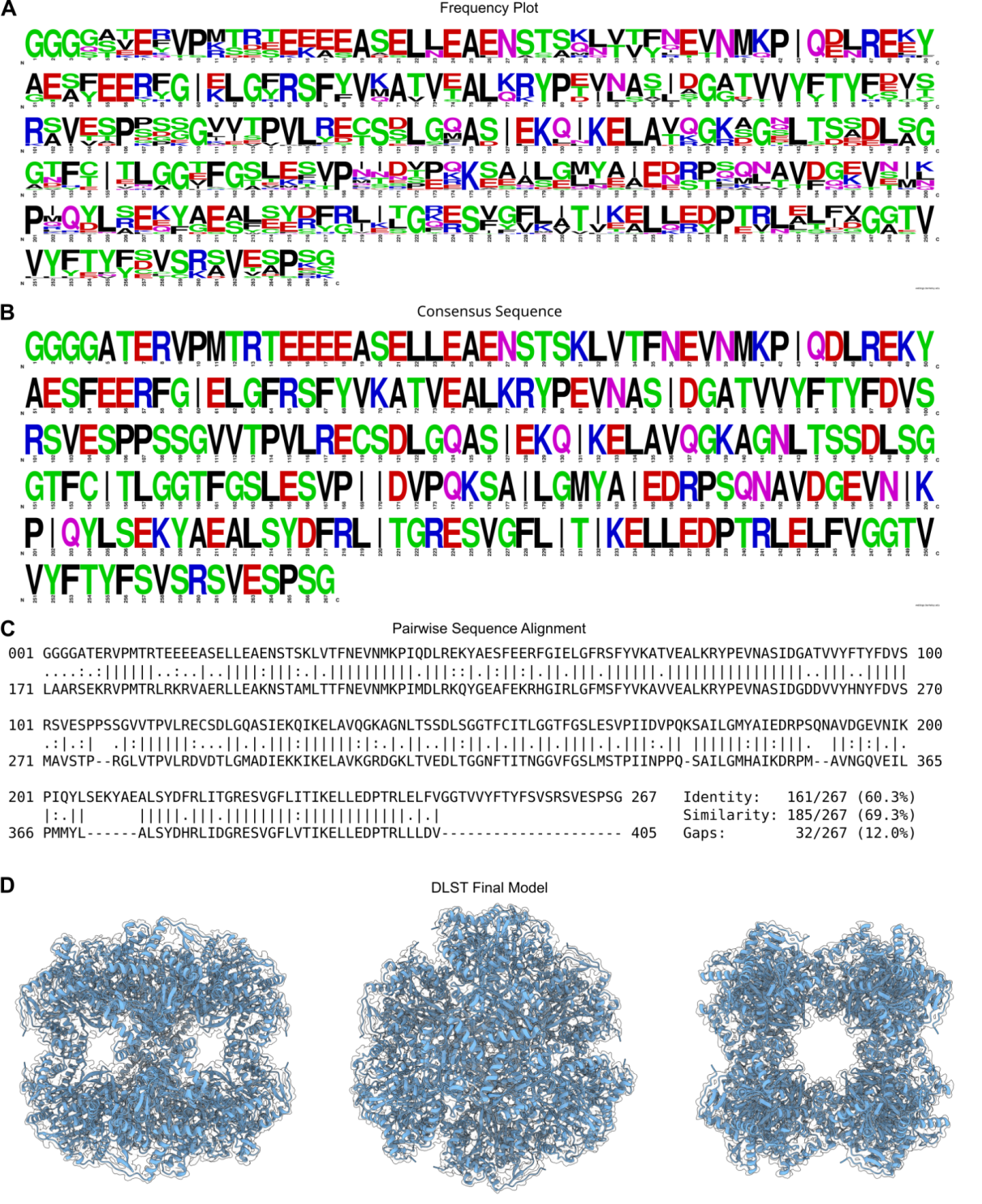


## Supplemental Figure 3. Sequences derived from ModelAngelo’s sequence-agnostic method used to identify and model DLST from the Cryo-EM volume map. (A) ModelAngelo sequence fragments were aligned in an MSA to generate a frequency plot displaying relative abundance of amino acids at each position. (B) A consensus sequence for one chain was generated using amino acid frequency data from (A) at each position. This consensus sequence was the input for Protein BLAST [[4]](https://paperpile.com/c/zLgsrK/wqRP). (C) After Protein BLAST identified DLST, UniProt was searched for the corresponding DLST sequence for the BL21 (DE3) expression vector. The UniProt sequence A0A140NDX4 was aligned with the consensus sequence [[17]](https://paperpile.com/c/zLgsrK/k3eZ). The first alignment revealed a gap spanning the first 170 residues of the UniProt sequence so an additional alignment was performed using only the UniProt residues at position 171 or later. (D) The final built model of the DLST catalytic domain docked into the 2.51 Å density map along the 2-, 3-, and 4-fold axes of symmetry.


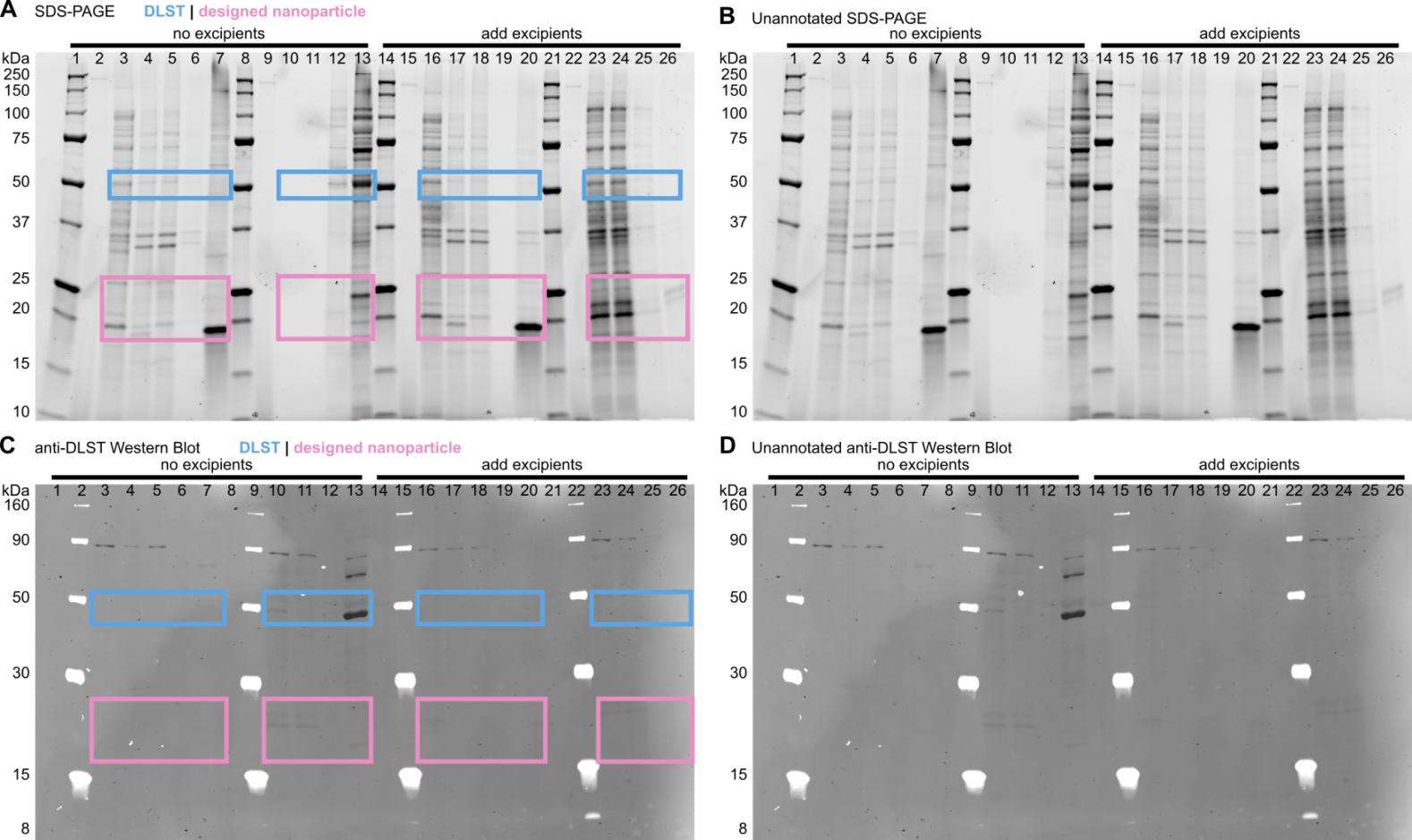


## Supplemental Figure 4. Full SDS-PAGE and Western Blot of improved purification of the designed two-component nanoparticle purified with and without excipients. Labels for individual lanes are located in Supplemental Table 4. (A-B) Annotated and Unannotated SDS-PAGE of IMAC purification of each component of the designed two-component nanoparticle. Both protein components of the designed nanoparticle fall between 25.7 kDa and 23.2 kDa and are generally represented by the pink boxes. The DLST catalytic subunit is labeled around 50 kDa by the blue boxes. (C-D) Annotated and unannotated anti-DLST Western Blot from the SDS-PAGE gel transfer.


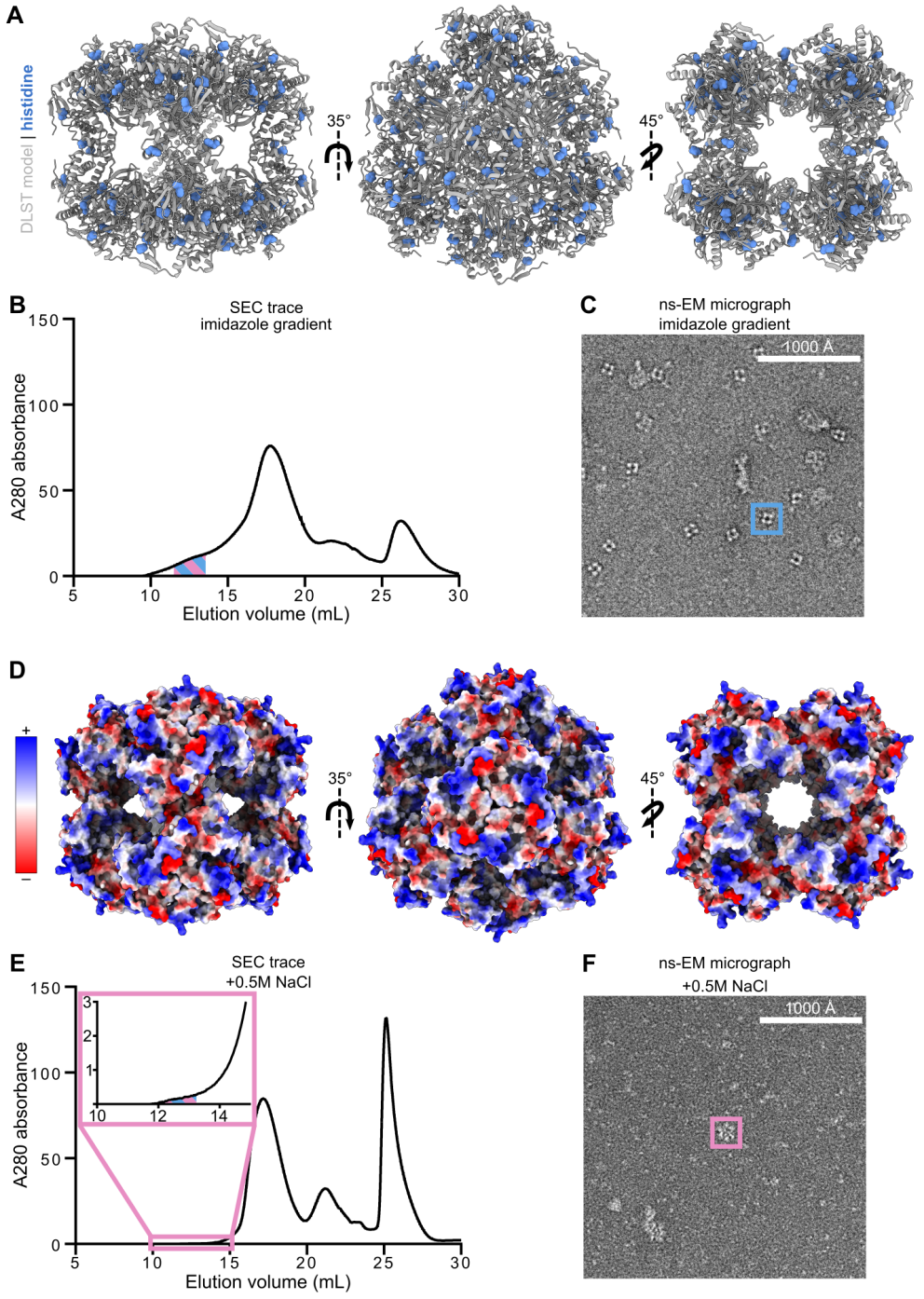


## Supplemental Figure 5. Improving purification of the designed nanoparticle through modification of purification protocols to remove DLST. Various purification attempts of the on-target nanoparticle were conducted, evaluating buffer conditions to eliminate the co-purification of DLST. (A) Location of histidines (blue) in DLST along the 2-, 3-, and 4-fold axis of symmetry. (B) SEC trace of the designed nanoparticle after protein components were purified with an elution gradient from 0.00 M - 0.50 M imidazole. (C) The highlighted fraction from SEC purification was taken for ns-EM. This is a cropped view of a ns-EM micrograph taken at 57,000x magnification. The majority of observed particles were of DLST. (D) Electrostatic potential of the catalytic domain of DLST with positively charged residues shown in blue and negatively charged residues in red along the 2-, 3-, and 4-fold axes of symmetry. (E) SEC trace of the octahedral nanoparticle after cells were lysed with 0.5 M NaCl. IMAC buffers to purify components also contained 0.5 M NaCl. Zoomed-in window highlighting the SEC fraction taken for nsEM in the next panel. (F) The highlighted fraction from SEC where the expected elution time of the nanoparticle was taken for ns-EM. This is a cropped view of a ns-EM micrograph taken at 57,000x magnification. Particle total yield and total concentration of assembled material was significantly diminished Supplemental Table 1). We did not observe the presence of DLST in these micrographs. (Blue = contaminant protein, DLST; Pink = on-target two component nanoparticle).


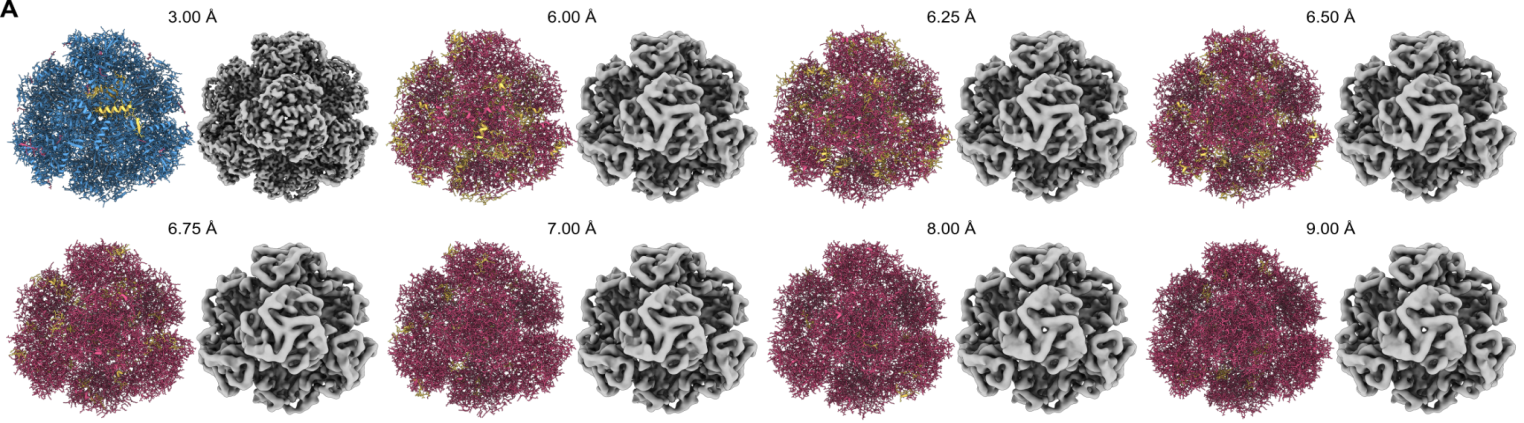


## Supplemental Figure 6. Additional maps and models. (A) Additional low-pass filtered maps and associated ModelAngelo output models between 3.00 Å and 9.00 Å not shown in Figure 4.


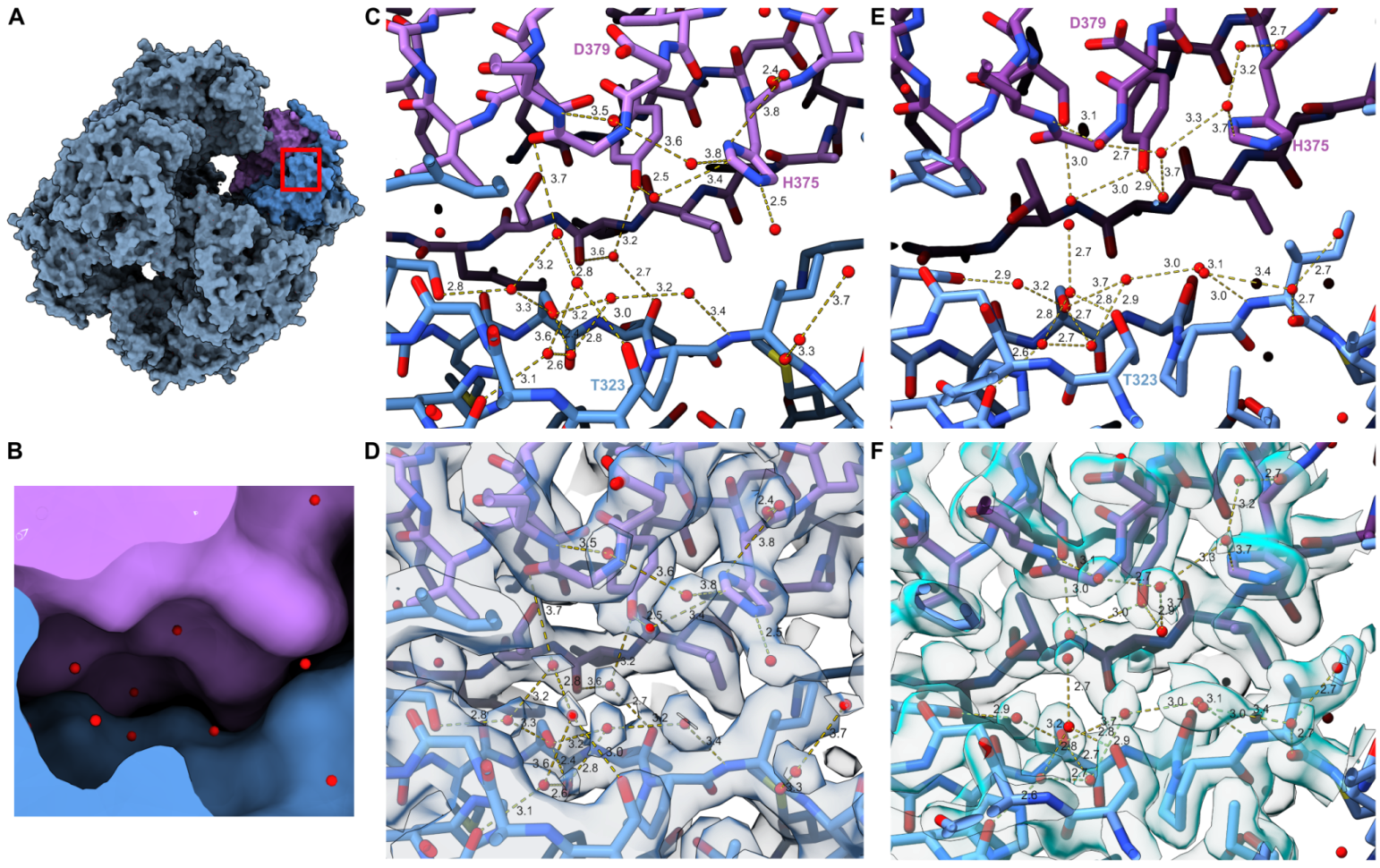


## Supplemental Figure 7. A comparison of the active site water network coordinates for DLST. Water network of the DLST active site comparing the 2.51 Å Cryo-EM structure discussed here with the 2.20 Å crystal structure (PDB: 1SCZ) [[20]](https://paperpile.com/c/zLgsrK/mTN1). The catalytic residues HIS375, ASP379, THR323 are labeled. (A) Our Cryo-EM structure surface view, with a red box showing the location of the active site. (B) A zoomed in view of the active site highlighted in (A) using a cutaway surface view. (C) The Cryo-EM structure of DLST built from the 2.51 Å Cryo-EM map. (D) The structure in (C) fit within the Cryo-EM map. (E) 1SCZ model taken from the deposited structure [[20]](https://paperpile.com/c/zLgsrK/mTN1). (F) 1SCZ fit within the 2Fo-Fc map [[20]](https://paperpile.com/c/zLgsrK/mTN1).


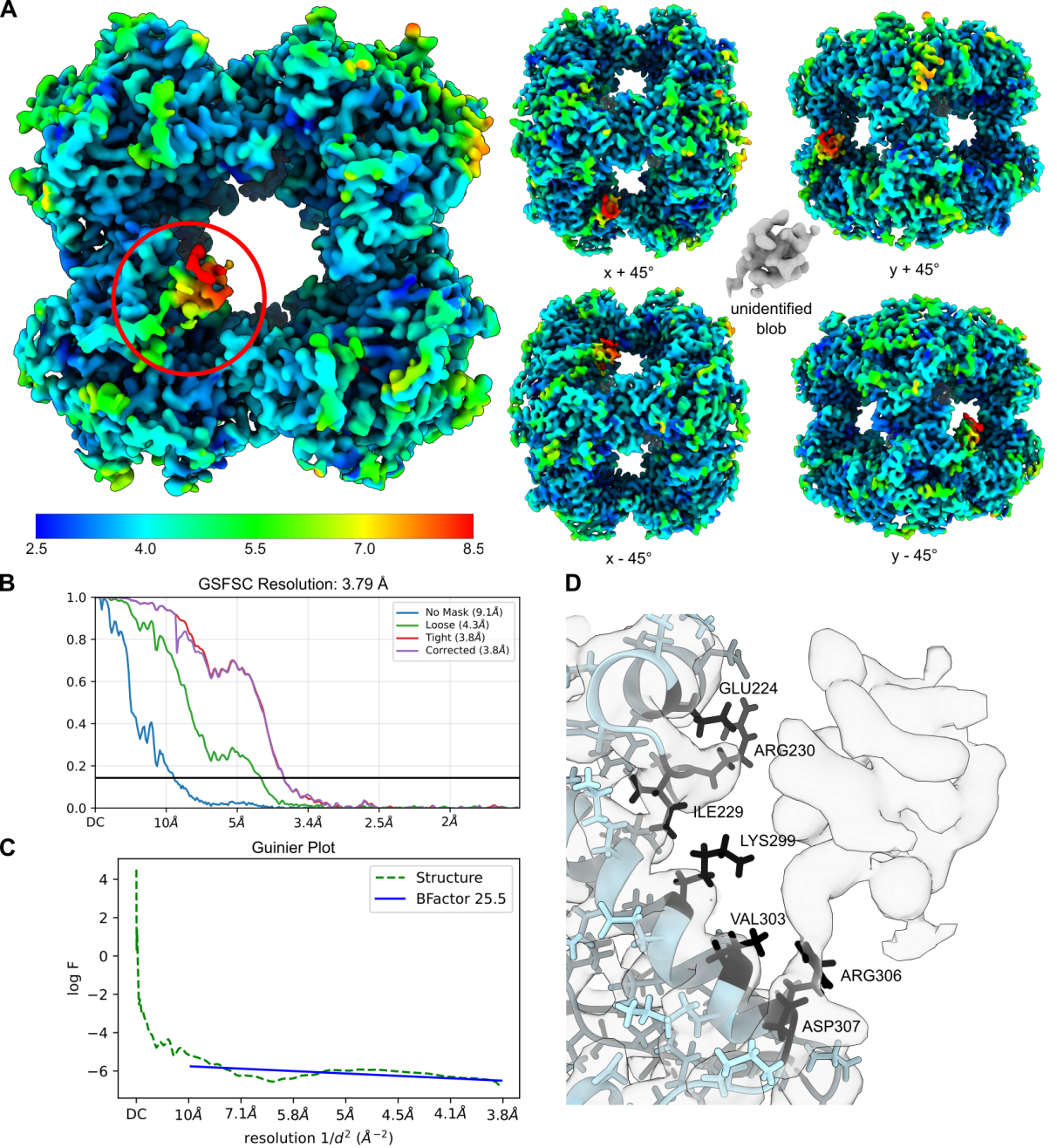


## Supplemental Figure 8. Asymmetric refinement of DLST catalytic domain. (A) Local resolution estimation of asymmetrical refined DLST catalytic domain (sharpened using DeepEMhancer) and the unidentified blob along the 4-fold axis, and the 2-fold axes nearest the face with the blob. Scale bar shows local resolution ranging from 2.50 Å (blue) to 8.50 Å (red). The unidentified blob is circled in red in the 4-fold axis image and as stand-alone density (grey) isolated from the rest of the model. (B) Gold Standard Fourier Shell Correlation (GSFSC) of the asymmetric map with a global resolution estimation of 3.79 Å at FSC 0.143. (C) Guinier Plot of the asymmetric map showing a BFactor of 25.5. (D) Cutaway-view showing the final DLST structure docked into the asymmetric density. The residues nearest to the blob were identified as GLU224, ILE 229, ARG230, LYS299, VAL303, ARG306, and ASP307.

# Supplemental Tables

## Supplemental Table 1. Estimated protein yields from different purification methods based on particles picked and sorted by ns-EM 2D classification

| **Purification Method** | **Total Yield (μg)** | **Designed Protein** | | | **Contaminant Protein** | | |
| --- | --- | --- | --- | --- | --- | --- | --- |
|  |  | **Particles Extracted (%)** | **Mass Extracted (%)** | **Estimated Protein  Yield (μg)** | **Particles Extracted (%)** | **Mass Extracted (%)** | **Estimated Protein Yield (μg)** |
| **Original Purification** | 97 | 3.53 | 3.91 | 3.79 | 96.5 | 96.1 | 93.2 |
| **+ 0 mM - 0.5 M Imidazole gradient** | 75 | -- | -- | -- | -- | -- | -- |
| **+ 0.5 mM NaCl** | Below detection for UVis | 1 particle observed | -- | -- | -- | -- | -- |
| **+ 100 mM Gly**  **+ 100 mM Arg** | 220 | 99.5 | 99.6 | 219 | 0.49 | 0.44 | 0.96 |
| ***Improvement w/ excipients (fold)*** | ***2.27*** | ***28.2*** | ***25.5*** | ***57.7*** | ***0.01*** | ***0.00*** | ***0.01*** |

## Supplemental Table 2. Cryo-EM Data Collection, Map, and Model Statistics.

***Sample Preparation***

Instrument Vitrobot Mk 4

Temperature (C) 22°

Humidity (%) 100

Blot Force (s) 0

Blot Time (s) 0.5

Wait Time (s) 7.5

Grid Type Quantifoil 2/2 Holey Carbon +2nm Carbon Film

***Data Collection***

Instrument FEI Titan Krios

Camera Gatan K3

Pixel Size (Å/pixel) 0.843

Energy Filter Bioquantum

Voltage (kV) 300

Dose Rate (e^-1^ pix^-1^ s^-1^) 8.4

Frame Rate (s) 0.05

Total Dose (e^-^/Å^2^) 47

Frames per Stack 75

Defocus Range (μm) 0.8 - 1.8

Movies Recorded 6,211

***Cryo-EM Map***

Particles in Final Map 18,809

Symmetry Octahedral

Global Resolution (Å) 2.51

Maximum Local Resolution (Å) 2.88

Minimum Local Resolution (Å) 2.44

***Atomic Model***

Number of Chains 24

Residues per Chain 233

Bond Lengths (Å) 0.004

Bond Angles 1.005

MolProbity Score 1.51

Clash Score 5.22

Ramachandran plot Outliers (%) 0.00

Ramachandran plot Allowed (%) 0.43

Ramachandran plot Favored (%) 99.57

Rotamer Outliers (%) 1.99

***Asymmetric Cryo-EM Map***

Particles in Final Map 19,033

Symmetry C1 / Asymmetric

Global Resolution (Å) 3.79

Maximum Local Resolution (Å) 2.50

Minimum Local Resolution (Å) 8.50

## Supplemental Table 3. Percent DLST identification using Protein BLAST (consensus sequence), hmmsearch (ModelAngelo), Protein BLAST (longest 10 chains), and hmmsearch (longest 10 chains).

|  | | **Protein BLAST (consensus sequence)** | **hmmsearch (ModelAngelo)** | **Protein BLAST (longest 10 Chains)** | | | | | | | | | | **hmmsearch (longest 10 chains)** | | | | | | | | | |
| --- | --- | --- | --- | --- | --- | --- | --- | --- | --- | --- | --- | --- | --- | --- | --- | --- | --- | --- | --- | --- | --- | --- | --- |
| Chain Number | | *consensus* | *all chains* | *1* | *2* | *3* | *4* | *5* | *6* | *7* | *8* | *9* | *10* | *1* | *2* | *3* | *4* | *5* | *6* | *7* | *8* | *9* | *10* |
| Map Resolution (Å) | 2.51 | 98.0 | 100 | 98.0 | 97.0 | 98.0 | 98.0 | 99.0 | 98.0 | 94.0 | 73.0 | 70.0 | 64.0 | 96.0 | 98.0 | 96.0 | 98.0 | 98.0 | 99.0 | 96.0 | 86.0 | 82.0 | 87.0 |
|  | 3.00 | 99.0 | 66.7 | 99.0 | 99.0 | 99.0 | 99.0 | 99.0 | 99.0 | 99.0 | 98.0 | 98.0 | 99.0 | 98.0 | 98.0 | 98.0 | 97.0 | 97.0 | 98.0 | 98.0 | 97.0 | 98.0 | 97.0 |
|  | 4.00 | 99.0 | 75.0 | 98.0 | 99.0 | 99.0 | 99.0 | 99.0 | 99.0 | 98.0 | 99.0 | 99.0 | 99.0 | 97.0 | 97.0 | 98.0 | 97.0 | 98.0 | 97.0 | 98.0 | 97.0 | 98.0 | 97.0 |
|  | 4.25 | 98.0 | 82.9 | 99.0 | 98.0 | 98.0 | 98.0 | 98.0 | 94.0 | 99.0 | 99.0 | 99.0 | 93.0 | 97.0 | 93.0 | 95.0 | 86.0 | 94.0 | 86.0 | 95.0 | 93.0 | 87.0 | 98.0 |
|  | 4.50 | 95.0 | 97.00 | 97.0 | 98.0 | 98.0 | 98.0 | 92.0 | 90.0 | 88.0 | 87.0 | 91.0 | 92.0 | 94.0 | 95.0 | 95.0 | 94.0 | 96.0 | 94.0 | 96.0 | 95.0 | 94.0 | 96.0 |
|  | 4.75 | 97.0 | 100 | 98.0 | 93.0 | 93.0 | 88.0 | 80.0 | 86.0 | 30.0 | 27.0 | 86.0 | 36.0 | 95.0 | 99.0 | 96.0 | 97.0 | 95.0 | 95.0 | 96.0 | 96.0 | 95.0 | 96.0 |
|  | 5.00 | -- | 99.0 | 50.0 | 48.0 | 33.0 | 27.0 | 79.0 | 26.0 | 26.0 | 46.0 | 28.0 | 84.0 | 98.0 | 98.0 | 98.0 | 96.0 | 98.0 | 98.0 | 98.0 | 97.0 | 98.0 | 97.0 |
|  | 5.25 | -- | 91.5 | 95.0 | 100 | 93.0 | 92.0 | -- | -- | -- | 50.0 | -- | -- | 96.0 | 94.0 | 97.0 | 94.0 | 94.0 | 80.0 | 69.0 | 97.0 | 68.0 | 87.0 |
|  | 5.50 | -- | 62.7 | 78.0 | 93.3 | -- | -- | 2.00 | 0.00 | 0.00 | 2.00 | 0.00 | 0.00 | 93.0 | 95.0 | 89.0 | 79.0 | 86.0 | 20.4 | 26.0 | 31.0 | 0.00 | 0.00 |
|  | 5.75 | -- | 48.4 | -- | 0.00 | 0.00 | 0.00 | 0.00 | 18.0 | 0.00 | 0.00 | 0.00 | 0.00 | 91.0 | 0.00 | 93.88 | 0.00 | 0.00 | 81.0 | 92.0 | 0.00 | 0.00 | 0.00 |
|  | 6.00 | -- | 55.6 | 0.00 | 0.00 | 0.00 | 0.00 | 1.00 | 0.00 | 55.0 | 0.00 | 0.00 | 0.00 | 1.37 | 0.00 | 0.00 | 0.00 | 11.6 | 0.00 | 67.0 | 0.00 | 0.00 | 0.00 |
|  | 6.25 | -- | 38.5 | 0.00 | 0.00 | 0.00 | 0.00 | 0.00 | 0.00 | 0.00 | 0.00 | 0.00 | 0.00 | 0.00 | 0.00 | 79.0 | 0.00 | -- | 0.00 | 0.00 | 1.67 | 0.00 | 0.00 |
|  | 6.50 | -- | 50.0 | 1.00 | 0.00 | 0.00 | 79.0 | 0.00 | 0.00 | 0.00 | 0.00 | 0.00 | 0.00 | 8.16 | 0.00 | 0.00 | 81.0 | -- | 0.00 | 0.00 | 0.00 | 0.00 | 0.00 |
|  | 6.75 | -- | -- | 0.00 | 0.00 | 0.00 | 0.00 | 0.00 | 0.00 | 0.00 | 0.00 | 0.00 | 0.00 | 0.00 | 0.00 | 0.00 | 0.00 | 0.00 | 0.00 | 0.00 | 0.00 | 0.00 | 0.00 |
|  | 7.00 | -- | 0.00 | 0.00 | 0.00 | 0.00 | 0.00 | 0.00 | 0.00 | 0.00 | 0.00 | 0.00 | 0.00 | 0.00 | 0.00 | 0.00 | 0.00 | 0.00 | -- | 0.00 | 0.00 | 0.00 | 0.00 |
|  | 8.00 | -- | -- | 0.00 | 0.00 | 0.00 | 0.00 | 0.00 | 0.00 | 0.00 | 0.00 | 0.00 | 0.00 | 0.00 | 0.00 | 0.00 | 0.00 | 0.00 | -- | -- | -- | -- | -- |
|  | 9.00 | -- | -- | 0.00 | 0.00 | 0.00 | 0.00 | 0.00 | 0.00 | 0.00 | 0.00 | 0.00 | 0.00 | 0.00 | 0.00 | 0.00 | 0.00 | 0.00 | 0.00 | -- | -- | -- | 0.00 |

## Supplemental Table 4. SDS-PAGE and anti-DLST Western Blot lane annotations.

| Lane | Annotation |
| --- | --- |
| 1 | Bio-Rad Precision Plus Protein Unstained Standards |
| 2 | LiCor Chameleon® 700 Pre-stained Protein Ladder |
| 3 | 1:20 dilution nanoparticle component 1 soluble lysate |
| 4 | 1:10 dilution nanoparticle component 1 fraction loaded on IMAC |
| 5 | 1:5 dilution nanoparticle component 1 IMAC flowthrough |
| 6 | Nanoparticle component 1 IMAC wash |
| 7 | Nanoparticle component 1 IMAC eluate |
| 8 | Bio-Rad Precision Plus Protein Unstained Standards |
| 9 | LiCor Chameleon® 700 Pre-stained Protein Ladder |
| 10 | 1:20 dilution nanoparticle component 2 fraction loaded on IMAC |
| 11 | 1:20 dilution nanoparticle component 2 IMAC flowthrough |
| 12 | Nanoparticle component 2 IMAC wash |
| 13 | Nanoparticle component 2 IMAC eluate |
| 14 | Bio-Rad Precision Plus Protein Unstained Standards |
| 15 | LiCor Chameleon® 700 Pre-stained Protein Ladder |
| 16 | 1:20 dilution nanoparticle component 1 soluble lysate + excipients |
| 17 | 1:10 dilution nanoparticle component 1 fraction loaded on IMAC + excipients |
| 18 | 1:5 dilution nanoparticle component 1 IMAC flowthrough + excipients |
| 19 | Nanoparticle component 1 IMAC wash + excipients |
| 20 | Nanoparticle component 1 IMAC eluate + excipients |
| 21 | Bio-Rad Precision Plus Protein Unstained Standards |
| 22 | LiCor Chameleon® 700 Pre-stained Protein Ladder |
| 23 | 1:20 dilution nanoparticle component 2 fraction loaded on IMAC + excipients |
| 24 | 1:20 dilution nanoparticle component 2 IMAC flowthrough + excipients |
| 25 | Nanoparticle component 2 IMAC wash + excipients |
| 26 | Nanoparticle component 2 IMAC eluate + excipients |

## Sequences 1. Consensus Sequences for all ModelAngelo outputs

| Map Resolution (Å) | Consensus Sequence |
| --- | --- |
| 2.51 | GGGGATERVPMTRTEEEEASELLEAENSTSKLVTFNEVNMKPIQDLREKY AESFEERFGIELGFRSFYVKATVEALKRYPEVNASIDGATVVYFTYFDVS RSVESPPSSGVVTPVLRECSDLGQASIEKQIKELAVQGKAGNLTSSDLSG GTFCITLGGTFGSLESVPIIDVPQKSAILGMYAIEDRPSQNAVDGEVNIK PIQYLSEKYAEALSYDFRLITGRESVGFLITIKELLEDPTRLELFVGGTV VYFTYFSVSRSVESPSG |
| 3.00 | SGSEKRVPRSKERKKEADRLNEAKNSTAMLTTFQEVNMKPIQDLRKKYGE AFEKRYGIRLGFRSFYVKAVVEALKRYPEVLASIEGDNVVYYNYFEVSRA VETPSKGGLVTPVLREVSTLGMADIEKKIKELATKARDGKLTTNELEGGN FTITNGGTFGSLMSVPIINPDPRAAILGLYAIKDRPQAVNGQVEILPMQY LSLSYDFRLIDGRESVGFLVTIKELLEDPTRLLLFV |
| 4.00 | GRSEERVPRRSKERKKVAERLNEALNSTVMIVVFNSVNMKPIQDLRKKYA EAFEKRYGVRLGFRSYYVKAVVEALKRYPDVNASLEGDTVVYYNYFEVTK AVETRKDKGLVTPCLREVSTLGMADIEKKIKELAVRARSGKLTEEELSGG NFTITNSGEFGGALMSVPIINPPQAAILGMHSIKDRPQAVNGKVEILPKM YLSLSYDYRIISAREATGFLTTIKELLEDPTRLLLFL |
| 4.25 | GKSTERVPPRSKERKKVAERLLEALNSTTMISVFNSVDMKPIQELRKKYA EEFEKRYGVRLGFRSFYVKAVVEALKRHPEVMASLEGGTVVYYNYFCVSK AVEDPSKGLVTVCIREVESLSMAEIEKKIKELAEKAREGNLTENLLSGGK SFTITNDGEFGTLMTVPIINPAPNAAVLGMHSIKERPKAINGKVEILPKQ YLSLSYDYRIITAREATGFMTTIKELLEDVTRLILFG |
| 4.50 | SGSTERVPLRSKERKKLAERLNEALNSTSMTTVFVEVNMKPIQELRKKYA EAFEKRRYGVRIGHRAFHIQAVVEALKRHPEIMASLEGDTVVYYTEFGVL MSKPVDNPNCAGLVTLCLRSIKDLPMAINGQVELLPQMYLALSYDYRIIT AREAVGFLLKIKKLAEKAREGDITREILSGAGDTPKK |
| 4.75 | NDGTERVPPRDKQRKKEAERINKALNSSSMTTLFVEVNKKPIQELKKKYA EAFKKRYGGVKIGNRAFYVKAVVEGMQRYPEILASIDGGTVVYNTRGGVL MSIPVPNPNCAAVVLSIRNVEDLSLAEIEKKIKALNGQVELLPEKWLALS YDRRIITAKRARTGFITEIKRRLLEDLERLLLGGG |
| 5.00 | IDIRAKGTESVPIQEKRKKEAEAFEKAYGIKILGLRAFRLSLPVPGPVNR YPELLGAYSPKTRPKANNGKVEVLPCSWLELEYSRRVVEARKLAEGFLEE VKRELAEVARSGNITEELLLGGGA |
| 5.25 | GIPVPNPNSGDGVSYYTRFSVVGVSRLVNGKIEVTLLDDGKAVTGFVPSP LKAREAELRYEYAEELEERLYGDRGIGGLRPEWLKAVAALALRRYPSILG KPEVTPVIYVLLTPRGLLLVI |
| 5.50 | LFLGPDLGGAGGGLGIFTPLGSPSGKLVEVTPRLRELRKEYGEAYAKKYG LVPELVVRGFYLRIPADGBRAREAKRSVEKLLSSLAEEARSSVYSNPLSV A |
| 5.75 | ALKRLLSDVSRAELVREKRLKALLLPLKLLKALAKKYGFAAGRPPYGLGS PGKLRASGGLLPLIVVPNGGVGLGDD |
| 6.00 | FFEDDAAVVKEVEKRSFPSEAASRYRKELAEEALVLALSGLLLLLLPSGL GVVLLGVGRGGGGNGEPGATVGSPGEAEWAALL |
| 6.25 | LLEVVLAAVAVVRSDLLLYLLSLPDGGGGGGGIGKPNSFGGDPPDAAAEA ALEKEELEEALEKEAAYRGALLLALSLLAAALLLRL |
| 6.50 | QKKELAKSEAEEALAEEAKEEYAEEELELEEGELLGGLAGGAALGGGLLL AAERVVSA |
| 6.75 | SNEAAAAEKEEAKALGLGEAGGAEKAALLALLEAELLLLLLLVRRPDY |
| 7.00 | ASLLALAALGGLKIVGGDGAEEEELLELLAGLLALLLLGSRT |
| 8.00 | NTLLLLGLLLLLGKLAAAEEEEELLEELEELLEEAAA |
| 9.00 | QARAGGSNLKELLEEEEEKEELEEELERLLLALGLLLADDLLDK |

##

## Supplemental Table 5. Number and percentage of total residues output by ModelAngelo belonging to chains of a given length

| Model Resolution (Å) | Total residues per chain length (CL) | | | | Percentage of residues per chain length (CL) | | |
| --- | --- | --- | --- | --- | --- | --- | --- |
|  | CL 1 - 10 | CL 11 - 100 | CL 101+ | Sum | CL 1 - 10 | CL 11 - 100 | CL 101+ |
| 2.51 | 70 | 84 | 5482 | 5636 | 1.242 | 1.490 | 97.268 |
| 3.00 | 70 | 151 | 5404 | 5625 | 1.244 | 2.684 | 96.071 |
| 4.00 | 80 | 892 | 4621 | 5593 | 1.430 | 15.949 | 82.621 |
| 4.25 | 155 | 2378 | 3044 | 5577 | 2.779 | 42.639 | 54.581 |
| 4.50 | 247 | 3867 | 1493 | 5607 | 4.405 | 68.967 | 26.627 |
| 4.75 | 398 | 4850 | 345 | 5593 | 7.116 | 86.716 | 6.168 |
| 5.00 | 1603 | 4081 | 0 | 5684 | 28.202 | 71.798 | 0.000 |
| 5.25 | 2332 | 3426 | 0 | 5758 | 40.500 | 59.500 | 0.000 |
| 5.50 | 3254 | 2706 | 0 | 5960 | 54.597 | 45.403 | 0.000 |
| 5.75 | 4679 | 1663 | 0 | 6342 | 73.778 | 26.222 | 0.000 |
| 6.00 | 5549 | 1180 | 0 | 6729 | 82.464 | 17.536 | 0.000 |
| 6.25 | 5758 | 1166 | 0 | 6924 | 83.160 | 16.840 | 0.000 |
| 6.50 | 6741 | 781 | 0 | 7522 | 89.617 | 10.383 | 0.000 |
| 6.75 | 7443 | 495 | 0 | 7938 | 93.764 | 6.236 | 0.000 |
| 7.00 | 7569 | 298 | 0 | 7867 | 96.212 | 3.788 | 0.000 |
| 8.00 | 7217 | 129 | 0 | 7346 | 98.244 | 1.756 | 0.000 |
| 9.00 | 7163 | 184 | 0 | 7347 | 97.496 | 2.504 | 0.000 |

##

## Supplemental Table 6. Number of chains of a given length output by ModelAngelo

| **Model Resolution (Å)** | **Number of chains** | | | **Total number of chains** |
| --- | --- | --- | --- | --- |
|  | **1 - 10 residues** | **11 - 100 residues** | **101+ residues** |  |
| *2.51* | 50 | 1 | 41 | 92 |
| *3.00* | 51 | 2 | 26 | 79 |
| *4.00* | 52 | 14 | 25 | 91 |
| *4.25* | 92 | 39 | 20 | 151 |
| *4.50* | 131 | 85 | 12 | 228 |
| *4.75* | 176 | 107 | 3 | 286 |
| *5.00* | 471 | 162 | 0 | 633 |
| *5.25* | 843 | 179 | 0 | 1022 |
| *5.50* | 1291 | 155 | 0 | 1446 |
| *5.75* | 1780 | 106 | 0 | 1886 |
| *6.00* | 2210 | 84 | 0 | 2294 |
| *6.25* | 2295 | 80 | 0 | 2375 |
| *6.50* | 2751 | 56 | 0 | 2807 |
| *6.75* | 3141 | 38 | 0 | 3179 |
| *7.00* | 3179 | 23 | 0 | 3202 |
| *8.00* | 3103 | 10 | 0 | 3113 |
| *9.00* | 3116 | 14 | 0 | 3130 |

## Supplemental Table 7. Protein BLAST results based on consensus sequence of automated model building on a 2.51 Å sharpened volume map

| **Description** | **Scientific Name** | **Max Score** | **Total Score** | **Query Cover** | **E value** | **Per. ident** | **Acc. Len** | **Accession** |
| --- | --- | --- | --- | --- | --- | --- | --- | --- |
| dihydrolipoyllysine-residue succinyltransferase [Salmonella enterica subsp. enterica serovar Virginia] | Salmonella enterica subsp. enterica serovar Virginia | 308 | 308 | 91% | 4.00E-102 | 66.53 | 237 | MEA7606579.1 |
| dihydrolipoamide succinyltransferase component (E2) [Salmonella enterica subsp. enterica serovar Heidelberg] | Salmonella enterica subsp. enterica serovar Heidelberg | 308 | 308 | 91% | 5.00E-102 | 66.53 | 258 | SQJ29857.1 |
| dihydrolipoyllysine-residue succinyltransferase [Salmonella enterica] | Salmonella enterica | 308 | 308 | 91% | 6.00E-102 | 66.53 | 241 | EEN7692697.1 |
| dihydrolipoyllysine-residue succinyltransferase [Salmonella enterica subsp. enterica serovar Wilhelmsburg] | Salmonella enterica subsp. enterica serovar Wilhelmsburg | 308 | 308 | 91% | 6.00E-102 | 66.53 | 257 | TGC90782.1 |
| dihydrolipoyllysine-residue succinyltransferase [Salmonella enterica subsp. enterica serovar Typhimurium] | Salmonella enterica subsp. enterica serovar Typhimurium | 308 | 308 | 91% | 7.00E-102 | 66.53 | 260 | MBZ5059444.1 |
| dihydrolipoyllysine-residue succinyltransferase [Escherichia coli O157:H7] | Escherichia coli O157:H7 | 307 | 307 | 89% | 7.00E-102 | 67.08 | 240 | EFC3643281.1 |
| dihydrolipoyllysine-residue succinyltransferase [Salmonella enterica subsp. enterica serovar Typhimurium] | Salmonella enterica subsp. enterica serovar Typhimurium | 307 | 307 | 89% | 7.00E-102 | 67.5 | 233 | ECY4747990.1 |
| dihydrolipoyllysine-residue succinyltransferase [Salmonella enterica] | Salmonella enterica | 308 | 308 | 91% | 7.00E-102 | 66.53 | 259 | WP_080206540.1 |
| dihydrolipoyllysine-residue succinyltransferase [Escherichia coli] | Escherichia coli | 307 | 307 | 89% | 7.00E-102 | 67.08 | 235 | WP_250378311.1 |
| dihydrolipoyllysine-residue succinyltransferase [Escherichia coli] | Escherichia coli | 307 | 307 | 89% | 7.00E-102 | 67.08 | 234 | WP_149465242.1 |
| TPA: dihydrolipoyllysine-residue succinyltransferase [Salmonella enterica] | Salmonella enterica | 308 | 308 | 91% | 8.00E-102 | 66.53 | 263 | HGE8117794.1 |
| dihydrolipoyllysine-residue succinyltransferase [Salmonella enterica subsp. enterica serovar Reading] | Salmonella enterica subsp. enterica serovar Reading | 307 | 307 | 91% | 8.00E-102 | 66.53 | 241 | EDV1620825.1 |
| dihydrolipoyllysine-residue succinyltransferase [Escherichia coli O177] | Escherichia coli O177 | 307 | 307 | 89% | 8.00E-102 | 67.08 | 229 | EFA8854754.1 |
| dihydrolipoyllysine-residue succinyltransferase [Escherichia] | Escherichia | 307 | 307 | 89% | 8.00E-102 | 67.08 | 236 | WP_000629458.1 |
| dihydrolipoyllysine-residue succinyltransferase [Salmonella enterica] | Salmonella enterica | 307 | 307 | 89% | 8.00E-102 | 67.5 | 231 | WP_128277741.1 |
| TPA: dihydrolipoyllysine-residue succinyltransferase [Escherichia coli] | Escherichia coli | 307 | 307 | 89% | 8.00E-102 | 67.08 | 240 | HDY2775515.1 |
| dihydrolipoyllysine-residue succinyltransferase [Salmonella enterica] | Salmonella enterica | 307 | 307 | 89% | 9.00E-102 | 67.5 | 232 | WP_080094185.1 |
| Dihydrolipoamide succinyltransferase [Salmonella enterica subsp. enterica serovar Wandsworth str. A4-580] | Salmonella enterica subsp. enterica serovar Wandsworth str. A4-580 | 308 | 308 | 91% | 9.00E-102 | 66.53 | 266 | EHD05307.1 |
| dihydrolipoyllysine-residue succinyltransferase [Escherichia coli] | Escherichia coli | 306 | 306 | 89% | 9.00E-102 | 67.08 | 230 | EGE7843444.1 |
| hypothetical protein SM286126_07181 [Salmonella enterica subsp. enterica serovar Typhi] | Salmonella enterica subsp. enterica serovar Typhi | 308 | 308 | 91% | 9.00E-102 | 66.53 | 262 | AYU11211.1 |
| dihydrolipoyllysine-residue succinyltransferase [Escherichia coli] | Escherichia coli | 307 | 307 | 89% | 1.00E-101 | 67.08 | 237 | WP_252389303.1 |
| dihydrolipoyllysine-residue succinyltransferase [Escherichia coli] | Escherichia coli | 307 | 307 | 89% | 1.00E-101 | 67.08 | 233 | WP_136823828.1 |
| dihydrolipoyllysine-residue succinyltransferase [Escherichia coli] | Escherichia coli | 307 | 307 | 89% | 1.00E-101 | 67.08 | 241 | EIH9592508.1 |
| dihydrolipoyllysine-residue succinyltransferase [Escherichia coli] | Escherichia coli | 307 | 307 | 89% | 1.00E-101 | 67.08 | 238 | EFO3983716.1 |
| dihydrolipoamide succinyltransferase [Salmonella enterica subsp. enterica serovar Agona str. 442692 2-4] | Salmonella enterica subsp. enterica serovar Agona str. 442692 2-4 | 308 | 308 | 91% | 1.00E-101 | 66.53 | 281 | ESB33469.1 |
| dihydrolipoyllysine-residue succinyltransferase [Escherichia coli] | Escherichia coli | 307 | 307 | 89% | 1.00E-101 | 67.08 | 239 | EEZ3479170.1 |
| dihydrolipoyllysine-residue succinyltransferase [Escherichia coli O157:H7] | Escherichia coli O157:H7 | 306 | 306 | 89% | 1.00E-101 | 67.08 | 231 | EEW1174705.1 |
| dihydrolipoyllysine-residue succinyltransferase [Escherichia coli] | Escherichia coli | 306 | 306 | 89% | 1.00E-101 | 67.08 | 232 | ENT2450297.1 |
| 2-oxoglutarate dehydrogenase complex dihydrolipoyllysine-residue succinyltransferase [Salmonella enterica] | Salmonella enterica | 308 | 308 | 91% | 1.00E-101 | 66.53 | 283 | WP_058818759.1 |
| dihydrolipoyllysine-residue succinyltransferase [Escherichia coli] | Escherichia coli | 306 | 306 | 89% | 1.00E-101 | 66.67 | 235 | WP_122792276.1 |
| dihydrolipoyllysine-residue succinyltransferase [Salmonella enterica subsp. enterica] | Salmonella enterica subsp. enterica | 308 | 308 | 91% | 1.00E-101 | 66.53 | 266 | ECE6616558.1 |
| dihydrolipoyllysine-residue succinyltransferase [Escherichia coli] | Escherichia coli | 307 | 307 | 89% | 1.00E-101 | 67.08 | 243 | TJB60735.1 |
| dihydrolipoyllysine-residue succinyltransferase [Escherichia coli] | Escherichia coli | 307 | 307 | 89% | 1.00E-101 | 67.08 | 248 | KAA0647049.1 |
| dihydrolipoyllysine-residue succinyltransferase [Escherichia coli] | Escherichia coli | 306 | 306 | 89% | 1.00E-101 | 67.36 | 228 | WP_229024141.1 |
| dihydrolipoyllysine-residue succinyltransferase [Escherichia coli] | Escherichia coli | 307 | 307 | 89% | 1.00E-101 | 67.08 | 257 | MCU7719730.1 |
| dihydrolipoyllysine-residue succinyltransferase [Escherichia coli] | Escherichia coli | 307 | 307 | 89% | 1.00E-101 | 67.08 | 259 | EIC1734575.1 |
| 2-oxoglutarate dehydrogenase complex dihydrolipoyllysine-residue succinyltransferase [Salmonella enterica] | Salmonella enterica | 308 | 308 | 91% | 1.00E-101 | 66.53 | 281 | WP_024144429.1 |
| TPA: dihydrolipoyllysine-residue succinyltransferase [Escherichia coli] | Escherichia coli | 307 | 307 | 89% | 1.00E-101 | 67.08 | 261 | HAM4331889.1 |
| TPA: 2-oxoglutarate dehydrogenase complex dihydrolipoyllysine-residue succinyltransferase [Salmonella enterica] | Salmonella enterica | 308 | 308 | 91% | 1.00E-101 | 66.53 | 273 | HGE8103874.1 |
| dihydrolipoyllysine-residue succinyltransferase [Escherichia coli] | Escherichia coli | 307 | 307 | 89% | 1.00E-101 | 67.08 | 255 | WP_042066964.1 |
| dihydrolipoyllysine-residue succinyltransferase [Salmonella enterica subsp. enterica] | Salmonella enterica subsp. enterica | 307 | 307 | 89% | 1.00E-101 | 67.08 | 249 | EHK8207480.1 |
| dihydrolipoyllysine-residue succinyltransferase [Escherichia coli] | Escherichia coli | 307 | 307 | 89% | 2.00E-101 | 67.08 | 250 | WP_323653019.1 |
| dihydrolipoyllysine-residue succinyltransferase [Salmonella enterica subsp. enterica serovar Enteritidis] | Salmonella enterica subsp. enterica serovar Enteritidis | 306 | 306 | 89% | 2.00E-101 | 67.08 | 233 | EIF4604241.1 |
| dihydrolipoyllysine-residue succinyltransferase [Escherichia coli] | Escherichia coli | 307 | 307 | 89% | 2.00E-101 | 67.08 | 256 | MCJ8642909.1 |
| dihydrolipoyllysine-residue succinyltransferase [Escherichia coli] | Escherichia coli | 306 | 306 | 89% | 2.00E-101 | 67.08 | 242 | WP_149518815.1 |
| TPA: dihydrolipoyllysine-residue succinyltransferase [Citrobacter freundii] | Citrobacter freundii | 306 | 306 | 91% | 2.00E-101 | 66.12 | 245 | HBI3683921.1 |
| 2-oxoglutarate dehydrogenase complex dihydrolipoyllysine-residue succinyltransferase [Salmonella enterica] | Salmonella enterica | 308 | 308 | 91% | 2.00E-101 | 66.53 | 277 | ENQ4548694.1 |
| dihydrolipoyllysine-residue succinyltransferase [Escherichia coli] | Escherichia coli | 306 | 306 | 89% | 2.00E-101 | 67.08 | 243 | EEX5691720.1 |
| TPA: dihydrolipoyllysine-residue succinyltransferase [Escherichia coli] | Escherichia coli | 307 | 307 | 89% | 2.00E-101 | 67.08 | 254 | HDC0239447.1 |
| dihydrolipoamide succinyltransferase component (E2) [Salmonella bongori] | Salmonella bongori | 306 | 306 | 89% | 2.00E-101 | 67.5 | 235 | VDZ80103.1 |
| 2-oxoglutarate dehydrogenase complex dihydrolipoyllysine-residue succinyltransferase [Salmonella enterica subsp. enterica serovar Virginia] | Salmonella enterica subsp. enterica serovar Virginia | 308 | 308 | 91% | 2.00E-101 | 66.53 | 290 | MEA5988320.1 |
| hypothetical protein ECZU26_29390 [Escherichia coli] | Escherichia coli | 307 | 307 | 89% | 2.00E-101 | 67.08 | 260 | GHL32114.1 |
| TPA: dihydrolipoyllysine-residue succinyltransferase [Escherichia coli] | Escherichia coli | 306 | 306 | 89% | 2.00E-101 | 67.08 | 244 | HBB1186217.1 |
| dihydrolipoyllysine-residue succinyltransferase [Salmonella enterica] | Salmonella enterica | 307 | 307 | 91% | 2.00E-101 | 66.53 | 267 | WP_052938831.1 |
| dihydrolipoyllysine-residue succinyltransferase [Salmonella enterica subsp. enterica serovar Typhimurium var. 5-] | Salmonella enterica subsp. enterica serovar Typhimurium var. 5- | 308 | 308 | 91% | 2.00E-101 | 66.53 | 272 | EDT4416474.1 |
| dihydrolipoyllysine-residue succinyltransferase [Escherichia coli] | Escherichia coli | 306 | 306 | 89% | 2.00E-101 | 67.08 | 245 | WP_136775487.1 |
| 2-oxoglutarate dehydrogenase complex dihydrolipoyllysine-residue succinyltransferase [Escherichia coli] | Escherichia coli | 308 | 308 | 89% | 2.00E-101 | 67.08 | 282 | MDY9040892.1 |
| 2-oxoglutarate dehydrogenase complex dihydrolipoyllysine-residue succinyltransferase [Salmonella enterica subsp. enterica serovar Typhimurium] | Salmonella enterica subsp. enterica serovar Typhimurium | 308 | 308 | 91% | 2.00E-101 | 66.53 | 301 | MBZ5045664.1 |
| 2-oxoglutarate dehydrogenase complex dihydrolipoyllysine-residue succinyltransferase [Salmonella enterica] | Salmonella enterica | 308 | 308 | 91% | 2.00E-101 | 66.53 | 294 | MEX8985164.1 |
| dihydrolipoyllysine-residue succinyltransferase [Escherichia coli O145:H28] | Escherichia coli O145:H28 | 306 | 306 | 89% | 2.00E-101 | 67.08 | 246 | EJH5192981.1 |
| dihydrolipoyllysine-residue succinyltransferase [Escherichia coli] | Escherichia coli | 307 | 307 | 89% | 2.00E-101 | 67.08 | 260 | WP_252391012.1 |
| dihydrolipoyllysine-residue succinyltransferase [Escherichia coli] | Escherichia coli | 306 | 306 | 89% | 2.00E-101 | 67.08 | 247 | WP_185168965.1 |
| 2-oxoglutarate dehydrogenase complex dihydrolipoyllysine-residue succinyltransferase [Salmonella enterica subsp. enterica serovar Typhimurium] | Salmonella enterica subsp. enterica serovar Typhimurium | 309 | 309 | 91% | 2.00E-101 | 66.53 | 311 | MBZ4776989.1 |
| dihydrolipoyllysine-residue succinyltransferase [Salmonella enterica] | Salmonella enterica | 307 | 307 | 91% | 2.00E-101 | 66.53 | 269 | EBF1873699.1 |
| dihydrolipoyllysine-residue succinyltransferase [Escherichia coli] | Escherichia coli | 307 | 307 | 89% | 2.00E-101 | 67.08 | 263 | WP_047654718.1 |
| dihydrolipoamide succinyltransferase [Escherichia coli] | Escherichia coli | 307 | 307 | 89% | 2.00E-101 | 67.08 | 263 | OWF26202.1 |
| TPA: dihydrolipoyllysine-residue succinyltransferase [Escherichia coli] | Escherichia coli | 307 | 307 | 89% | 2.00E-101 | 67.08 | 264 | HAN7662089.1 |
| dihydrolipoyllysine-residue succinyltransferase [Escherichia coli] | Escherichia coli | 306 | 306 | 89% | 2.00E-101 | 67.08 | 248 | TJB30469.1 |
| dihydrolipoyllysine-residue succinyltransferase [Escherichia coli] | Escherichia coli | 307 | 307 | 89% | 2.00E-101 | 67.08 | 259 | EGE6907964.1 |
| dihydrolipoyllysine-residue succinyltransferase [Escherichia coli] | Escherichia coli | 307 | 307 | 89% | 2.00E-101 | 67.08 | 259 | WP_119682542.1 |
| dihydrolipoyllysine-residue succinyltransferase [Escherichia coli O145:H28] | Escherichia coli O145:H28 | 307 | 307 | 89% | 2.00E-101 | 67.08 | 264 | EJH5270977.1 |
| 2-oxoglutarate dehydrogenase complex dihydrolipoyllysine-residue succinyltransferase [Salmonella enterica subsp. enterica serovar Heidelberg] | Salmonella enterica subsp. enterica serovar Heidelberg | 312 | 312 | 91% | 2.00E-101 | 67.21 | 401 | EBL6043066.1 |
| 2-oxoglutarate dehydrogenase complex dihydrolipoyllysine-residue succinyltransferase [Escherichia coli] | Escherichia coli | 308 | 308 | 89% | 2.00E-101 | 67.08 | 280 | WP_320759799.1 |
| dihydrolipoyllysine-residue succinyltransferase [Escherichia coli] | Escherichia coli | 306 | 306 | 89% | 2.00E-101 | 67.08 | 252 | MDY9032137.1 |
| TPA: dihydrolipoyllysine-residue succinyltransferase [Escherichia coli] | Escherichia coli | 306 | 306 | 89% | 2.00E-101 | 67.08 | 252 | HBD1696035.1 |
| dihydrolipoyllysine-residue succinyltransferase [Escherichia coli] | Escherichia coli | 306 | 306 | 89% | 2.00E-101 | 67.08 | 253 | TJT18798.1 |
| dihydrolipoyllysine-residue succinyltransferase [Escherichia coli] | Escherichia coli | 306 | 306 | 89% | 2.00E-101 | 67.08 | 253 | WP_136748022.1 |
| dihydrolipoyllysine-residue succinyltransferase [Escherichia coli] | Escherichia coli | 306 | 306 | 89% | 2.00E-101 | 67.08 | 253 | EFH6296858.1 |
| dihydrolipoamide succinyltransferase component (E2) [Escherichia coli] | Escherichia coli | 307 | 307 | 89% | 2.00E-101 | 67.08 | 269 | VFT70387.1 |
| 2-oxoglutarate dehydrogenase complex dihydrolipoyllysine-residue succinyltransferase [Escherichia coli] | Escherichia coli | 307 | 307 | 89% | 2.00E-101 | 67.08 | 274 | MDY8964183.1 |
| TPA: dihydrolipoyllysine-residue succinyltransferase [Escherichia coli] | Escherichia coli | 307 | 307 | 89% | 2.00E-101 | 67.08 | 265 | HAP0635211.1 |
| dihydrolipoamide succinyltransferase component (E2) [Salmonella enterica subsp. enterica serovar Bovismorbificans] | Salmonella enterica subsp. enterica serovar Bovismorbificans | 308 | 308 | 91% | 2.00E-101 | 66.53 | 288 | CNT74736.1 |
| dihydrolipoyllysine-residue succinyltransferase [Salmonella enterica subsp. enterica] | Salmonella enterica subsp. enterica | 309 | 309 | 91% | 2.00E-101 | 66.53 | 314 | ECE5907583.1 |
| Dihydrolipoamide succinyltransferase [Salmonella enterica subsp. enterica serovar Alachua str. R6-377] | Salmonella enterica subsp. enterica serovar Alachua str. R6-377 | 308 | 308 | 91% | 2.00E-101 | 66.53 | 296 | EHC43100.1 |
| 2-oxoglutarate dehydrogenase complex dihydrolipoyllysine-residue succinyltransferase [Salmonella enterica] | Salmonella enterica | 309 | 309 | 91% | 2.00E-101 | 66.53 | 321 | MDX8924439.1 |
| 2-oxoglutarate dehydrogenase complex dihydrolipoyllysine-residue succinyltransferase [Escherichia coli] | Escherichia coli | 307 | 307 | 89% | 2.00E-101 | 67.08 | 269 | WP_115969540.1 |
| 2-oxoglutarate dehydrogenase complex dihydrolipoyllysine-residue succinyltransferase [Escherichia coli] | Escherichia coli | 307 | 307 | 89% | 2.00E-101 | 67.08 | 269 | WP_249524430.1 |
| 2-oxoglutarate dehydrogenase complex dihydrolipoyllysine-residue succinyltransferase [Escherichia coli] | Escherichia coli | 308 | 308 | 89% | 2.00E-101 | 67.08 | 286 | WP_047081876.1 |
| 2-oxoglutarate dehydrogenase complex dihydrolipoyllysine-residue succinyltransferase [Salmonella enterica] | Salmonella enterica | 308 | 308 | 91% | 2.00E-101 | 66.53 | 296 | WP_080186295.1 |
| TPA: 2-oxoglutarate dehydrogenase complex dihydrolipoyllysine-residue succinyltransferase [Salmonella enterica subsp. enterica] | Salmonella enterica subsp. enterica | 308 | 308 | 91% | 3.00E-101 | 66.53 | 309 | HAT5958997.1 |
| 2-oxoglutarate dehydrogenase complex dihydrolipoyllysine-residue succinyltransferase [Salmonella enterica] | Salmonella enterica | 308 | 308 | 91% | 3.00E-101 | 66.53 | 298 | WP_079839436.1 |
| 2-oxoglutarate dehydrogenase complex dihydrolipoyllysine-residue succinyltransferase [Escherichia coli] | Escherichia coli | 307 | 307 | 89% | 3.00E-101 | 67.08 | 274 | RIC38964.1 |
| dihydrolipoyllysine-residue succinyltransferase [Escherichia coli] | Escherichia coli | 306 | 306 | 89% | 3.00E-101 | 67.08 | 253 | WP_136801371.1 |
| 2-oxoglutarate dehydrogenase complex dihydrolipoyllysine-residue succinyltransferase [Escherichia coli] | Escherichia coli | 307 | 307 | 89% | 3.00E-101 | 67.08 | 270 | WP_250382326.1 |
| dihydrolipoamide succinyltransferase [Salmonella enterica subsp. enterica serovar Cerro str. 5569] | Salmonella enterica subsp. enterica serovar Cerro str. 5569 | 308 | 308 | 91% | 3.00E-101 | 66.53 | 307 | ETC75003.1 |
| 2-oxoglutarate dehydrogenase complex dihydrolipoyllysine-residue succinyltransferase [Escherichia coli] | Escherichia coli | 308 | 308 | 89% | 3.00E-101 | 67.08 | 286 | WP_060643349.1 |
| dihydrolipoamide succinyltransferase [Enterobacter asburiae] | Enterobacter asburiae | 307 | 307 | 91% | 3.00E-101 | 65.71 | 270 | STD18558.1 |
| 2-oxoglutarate dehydrogenase complex dihydrolipoyllysine-residue succinyltransferase [Escherichia coli] | Escherichia coli | 308 | 308 | 89% | 3.00E-101 | 67.08 | 295 | NAG00763.1 |
| 2-oxoglutarate dehydrogenase complex dihydrolipoyllysine-residue succinyltransferase [Salmonella enterica subsp. enterica serovar Typhimurium] | Salmonella enterica subsp. enterica serovar Typhimurium | 311 | 311 | 91% | 3.00E-101 | 66.94 | 402 | ECA8639535.1 |
| 2-oxoglutarate dehydrogenase complex dihydrolipoyllysine-residue succinyltransferase [Salmonella enterica subsp. enterica serovar Ibadan] | Salmonella enterica subsp. enterica serovar Ibadan | 309 | 309 | 91% | 3.00E-101 | 66.53 | 320 | ECY3951163.1 |

##

## Supplemental Table 8. Protein BLAST results based on consensus sequence of automated model building on a 3.00 Å low-pass filtered volume map

| **Description** | **Scientific Name** | **Max Score** | **Total Score** | **Query Cover** | **E value** | **Per. ident** | **Acc. Len** | **Accession** |
| --- | --- | --- | --- | --- | --- | --- | --- | --- |
| dihydrolipoyllysine-residue succinyltransferase [Escherichia coli O157:H7] | Escherichia coli O157:H7 | 379 | 379 | 99% | 1.00E-130 | 81.62 | 231 | EEW1174705.1 |
| dihydrolipoyllysine-residue succinyltransferase [Escherichia coli] | Escherichia coli | 379 | 379 | 99% | 1.00E-130 | 81.62 | 239 | EEZ3479170.1 |
| dihydrolipoyllysine-residue succinyltransferase [Escherichia coli O157:H7] | Escherichia coli O157:H7 | 379 | 379 | 99% | 1.00E-130 | 81.62 | 240 | EFC3643281.1 |
| dihydrolipoyllysine-residue succinyltransferase [Escherichia coli] | Escherichia coli | 379 | 379 | 99% | 1.00E-130 | 81.62 | 235 | WP_250378311.1 |
| TPA: dihydrolipoyllysine-residue succinyltransferase [Escherichia coli] | Escherichia coli | 379 | 379 | 99% | 1.00E-130 | 81.62 | 240 | HDY2775515.1 |
| dihydrolipoyllysine-residue succinyltransferase [Escherichia] | Escherichia | 379 | 379 | 99% | 1.00E-130 | 81.62 | 236 | WP_000629458.1 |
| dihydrolipoyllysine-residue succinyltransferase [Escherichia coli] | Escherichia coli | 379 | 379 | 99% | 1.00E-130 | 81.62 | 235 | WP_122792276.1 |
| dihydrolipoyllysine-residue succinyltransferase [Escherichia coli] | Escherichia coli | 379 | 379 | 99% | 1.00E-130 | 81.62 | 233 | WP_136823828.1 |
| dihydrolipoyllysine-residue succinyltransferase [Escherichia coli] | Escherichia coli | 379 | 379 | 99% | 1.00E-130 | 81.62 | 234 | WP_149465242.1 |
| dihydrolipoyllysine-residue succinyltransferase [Escherichia coli] | Escherichia coli | 379 | 379 | 99% | 1.00E-130 | 81.62 | 241 | EIH9592508.1 |
| dihydrolipoyllysine-residue succinyltransferase [Escherichia coli] | Escherichia coli | 379 | 379 | 99% | 1.00E-130 | 81.62 | 243 | TJB60735.1 |
| dihydrolipoyllysine-residue succinyltransferase [Escherichia coli] | Escherichia coli | 379 | 379 | 99% | 1.00E-130 | 81.62 | 238 | EFO3983716.1 |
| dihydrolipoyllysine-residue succinyltransferase [Escherichia coli] | Escherichia coli | 379 | 379 | 99% | 1.00E-130 | 81.62 | 237 | WP_252389303.1 |
| dihydrolipoyllysine-residue succinyltransferase [Escherichia coli] | Escherichia coli | 379 | 379 | 99% | 2.00E-130 | 81.62 | 259 | EIC1734575.1 |
| dihydrolipoyllysine-residue succinyltransferase [Escherichia coli] | Escherichia coli | 379 | 379 | 99% | 2.00E-130 | 81.62 | 248 | KAA0647049.1 |
| dihydrolipoyllysine-residue succinyltransferase [Escherichia coli] | Escherichia coli | 378 | 378 | 99% | 2.00E-130 | 81.62 | 232 | ENT2450297.1 |
| dihydrolipoyllysine-residue succinyltransferase [Salmonella enterica subsp. enterica] | Salmonella enterica subsp. enterica | 379 | 379 | 99% | 2.00E-130 | 81.62 | 249 | EHK8207480.1 |
| dihydrolipoyllysine-residue succinyltransferase [Escherichia coli] | Escherichia coli | 379 | 379 | 99% | 2.00E-130 | 81.62 | 260 | WP_252391012.1 |
| 2-oxoglutarate dehydrogenase complex dihydrolipoyllysine-residue succinyltransferase [Escherichia coli] | Escherichia coli | 380 | 380 | 99% | 2.00E-130 | 81.62 | 282 | MDY9040892.1 |
| dihydrolipoyllysine-residue succinyltransferase [Escherichia coli] | Escherichia coli | 379 | 379 | 99% | 2.00E-130 | 81.62 | 259 | EGE6907964.1 |
| dihydrolipoyllysine-residue succinyltransferase [Escherichia coli] | Escherichia coli | 379 | 379 | 99% | 2.00E-130 | 81.62 | 259 | WP_119682542.1 |
| hypothetical protein ECZU26_29390 [Escherichia coli] | Escherichia coli | 379 | 379 | 99% | 2.00E-130 | 81.62 | 260 | GHL32114.1 |
| TPA: dihydrolipoyllysine-residue succinyltransferase [Escherichia coli] | Escherichia coli | 379 | 379 | 99% | 2.00E-130 | 81.62 | 261 | HAM4331889.1 |
| dihydrolipoyllysine-residue succinyltransferase [Escherichia coli] | Escherichia coli | 379 | 379 | 99% | 2.00E-130 | 81.62 | 250 | WP_323653019.1 |
| dihydrolipoyllysine-residue succinyltransferase [Escherichia coli] | Escherichia coli | 379 | 379 | 99% | 2.00E-130 | 81.62 | 257 | MCU7719730.1 |
| dihydrolipoyllysine-residue succinyltransferase [Escherichia coli] | Escherichia coli | 379 | 379 | 99% | 2.00E-130 | 81.62 | 263 | WP_047654718.1 |
| dihydrolipoamide succinyltransferase [Escherichia coli] | Escherichia coli | 379 | 379 | 99% | 2.00E-130 | 81.62 | 263 | OWF26202.1 |
| dihydrolipoyllysine-residue succinyltransferase [Escherichia coli O145:H28] | Escherichia coli O145:H28 | 379 | 379 | 99% | 2.00E-130 | 81.62 | 264 | EJH5270977.1 |
| dihydrolipoyllysine-residue succinyltransferase [Escherichia coli] | Escherichia coli | 378 | 378 | 99% | 3.00E-130 | 81.62 | 242 | WP_149518815.1 |
| TPA: dihydrolipoyllysine-residue succinyltransferase [Escherichia coli] | Escherichia coli | 379 | 379 | 99% | 3.00E-130 | 81.62 | 264 | HAN7662089.1 |
| dihydrolipoamide succinyltransferase component (E2) [Escherichia coli] | Escherichia coli | 379 | 379 | 99% | 3.00E-130 | 81.62 | 269 | VFT70387.1 |
| 2-oxoglutarate dehydrogenase complex dihydrolipoyllysine-residue succinyltransferase [Escherichia coli] | Escherichia coli | 384 | 384 | 99% | 3.00E-130 | 81.62 | 405 | UIU60256.1 |
| dihydrolipoyllysine-residue succinyltransferase [Escherichia coli] | Escherichia coli | 378 | 378 | 99% | 3.00E-130 | 81.62 | 243 | EEX5691720.1 |
| TPA: dihydrolipoyllysine-residue succinyltransferase [Escherichia coli] | Escherichia coli | 379 | 379 | 99% | 3.00E-130 | 81.62 | 254 | HDC0239447.1 |
| TPA: dihydrolipoyllysine-residue succinyltransferase [Escherichia coli] | Escherichia coli | 378 | 378 | 99% | 3.00E-130 | 81.62 | 244 | HBB1186217.1 |
| dihydrolipoyllysine-residue succinyltransferase [Escherichia coli] | Escherichia coli | 379 | 379 | 99% | 3.00E-130 | 81.62 | 255 | WP_042066964.1 |
| dihydrolipoyllysine-residue succinyltransferase [Escherichia coli] | Escherichia coli | 378 | 378 | 99% | 3.00E-130 | 81.62 | 245 | WP_136775487.1 |
| dihydrolipoyllysine-residue succinyltransferase [Escherichia coli O145:H28] | Escherichia coli O145:H28 | 378 | 378 | 99% | 3.00E-130 | 81.62 | 246 | EJH5192981.1 |
| dihydrolipoamide succinyltransferase component (E2) [Escherichia coli] | Escherichia coli | 379 | 379 | 99% | 3.00E-130 | 81.62 | 269 | STG69599.1 |
| dihydrolipoyllysine-residue succinyltransferase [Escherichia coli] | Escherichia coli | 378 | 378 | 99% | 3.00E-130 | 81.62 | 247 | WP_185168965.1 |
| TPA: dihydrolipoyllysine-residue succinyltransferase [Escherichia coli] | Escherichia coli | 379 | 379 | 99% | 3.00E-130 | 81.62 | 265 | HAP0635211.1 |
| dihydrolipoyllysine-residue succinyltransferase [Escherichia coli] | Escherichia coli | 379 | 379 | 99% | 3.00E-130 | 81.62 | 256 | MCJ8642909.1 |
| dihydrolipoyllysine-residue succinyltransferase [Escherichia coli] | Escherichia coli | 378 | 378 | 99% | 3.00E-130 | 81.62 | 248 | TJB30469.1 |
| dihydrolipoamide succinyltransferase [Shigella sonnei] | Shigella sonnei | 380 | 380 | 99% | 3.00E-130 | 81.62 | 291 | CSF30971.1 |
| 2-oxoglutarate dehydrogenase complex dihydrolipoyllysine-residue succinyltransferase [Escherichia coli] | Escherichia coli | 380 | 380 | 99% | 3.00E-130 | 81.62 | 286 | WP_060643349.1 |
| TPA: 2-oxoglutarate dehydrogenase complex dihydrolipoyllysine-residue succinyltransferase [Escherichia coli] | Escherichia coli | 384 | 384 | 99% | 3.00E-130 | 82.05 | 405 | HDV4762163.1 |
| 2-oxoglutarate dehydrogenase complex dihydrolipoyllysine-residue succinyltransferase [Escherichia coli] | Escherichia coli | 379 | 379 | 99% | 4.00E-130 | 81.62 | 286 | WP_047081876.1 |
| 2-oxoglutarate dehydrogenase complex dihydrolipoyllysine-residue succinyltransferase [Escherichia coli] | Escherichia coli | 384 | 384 | 99% | 4.00E-130 | 82.05 | 405 | WP_103682200.1 |
| dihydrolipoyllysine-residue succinyltransferase [Escherichia coli] | Escherichia coli | 378 | 378 | 99% | 4.00E-130 | 81.62 | 252 | MDY9032137.1 |
| TPA: dihydrolipoyllysine-residue succinyltransferase [Escherichia coli] | Escherichia coli | 378 | 378 | 99% | 4.00E-130 | 81.62 | 252 | HBD1696035.1 |
| 2-oxoglutarate dehydrogenase complex dihydrolipoyllysine-residue succinyltransferase [Escherichia coli] | Escherichia coli | 379 | 379 | 99% | 4.00E-130 | 81.62 | 287 | EEZ2060122.1 |
| dihydrolipoyllysine-residue succinyltransferase [Escherichia coli] | Escherichia coli | 378 | 378 | 99% | 4.00E-130 | 81.62 | 253 | TJT18798.1 |
| dihydrolipoyllysine-residue succinyltransferase [Escherichia coli] | Escherichia coli | 378 | 378 | 99% | 4.00E-130 | 81.62 | 253 | WP_136748022.1 |
| dihydrolipoyllysine-residue succinyltransferase [Escherichia coli] | Escherichia coli | 378 | 378 | 99% | 4.00E-130 | 81.62 | 253 | EFH6296858.1 |
| 2-oxoglutarate dehydrogenase complex dihydrolipoyllysine-residue succinyltransferase [Escherichia coli] | Escherichia coli | 381 | 381 | 99% | 4.00E-130 | 81.62 | 321 | EEY6693670.1 |
| 2-oxoglutarate dehydrogenase complex dihydrolipoyllysine-residue succinyltransferase [Escherichia coli] | Escherichia coli | 381 | 381 | 99% | 4.00E-130 | 81.62 | 324 | EER8331471.1 |
| 2-oxoglutarate dehydrogenase complex dihydrolipoyllysine-residue succinyltransferase [Escherichia coli] | Escherichia coli | 381 | 381 | 99% | 4.00E-130 | 81.62 | 324 | MDO4358214.1 |
| 2-oxoglutarate dehydrogenase complex dihydrolipoyllysine-residue succinyltransferase [Escherichia coli] | Escherichia coli | 379 | 379 | 99% | 4.00E-130 | 81.62 | 275 | WP_136750337.1 |
| 2-oxoglutarate dehydrogenase complex dihydrolipoyllysine-residue succinyltransferase [Escherichia coli] | Escherichia coli | 380 | 380 | 99% | 4.00E-130 | 81.62 | 303 | EEY6784672.1 |
| 2-oxoglutarate dehydrogenase complex dihydrolipoyllysine-residue succinyltransferase [Escherichia coli] | Escherichia coli | 384 | 384 | 99% | 4.00E-130 | 82.05 | 405 | EFD4584379.1 |
| 2-oxoglutarate dehydrogenase complex dihydrolipoyllysine-residue succinyltransferase [Escherichia coli] | Escherichia coli | 380 | 380 | 99% | 4.00E-130 | 81.62 | 295 | NAG00763.1 |
| 2-oxoglutarate dehydrogenase complex dihydrolipoyllysine-residue succinyltransferase [Escherichia coli] | Escherichia coli | 380 | 380 | 99% | 4.00E-130 | 81.62 | 322 | WP_194154148.1 |
| 2-oxoglutarate dehydrogenase complex dihydrolipoyllysine-residue succinyltransferase [Escherichia coli] | Escherichia coli | 380 | 380 | 99% | 4.00E-130 | 81.62 | 322 | MCU8601182.1 |
| TPA: 2-oxoglutarate dehydrogenase complex dihydrolipoyllysine-residue succinyltransferase [Escherichia coli] | Escherichia coli | 380 | 380 | 99% | 4.00E-130 | 81.62 | 312 | HCO0761733.1 |
| 2-oxoglutarate dehydrogenase complex dihydrolipoyllysine-residue succinyltransferase [Escherichia coli] | Escherichia coli | 380 | 380 | 99% | 4.00E-130 | 81.62 | 318 | MCX0506393.1 |
| dihydrolipoyllysine-residue succinyltransferase [Escherichia coli] | Escherichia coli | 378 | 378 | 99% | 4.00E-130 | 81.62 | 253 | WP_136801371.1 |
| 2-oxoglutarate dehydrogenase complex dihydrolipoyllysine-residue succinyltransferase [Escherichia coli] | Escherichia coli | 379 | 379 | 99% | 5.00E-130 | 81.62 | 270 | WP_250382326.1 |
| 2-oxoglutarate dehydrogenase complex dihydrolipoyllysine-residue succinyltransferase [Escherichia coli] | Escherichia coli | 379 | 379 | 99% | 5.00E-130 | 81.62 | 269 | WP_115969540.1 |
| 2-oxoglutarate dehydrogenase complex dihydrolipoyllysine-residue succinyltransferase [Escherichia coli] | Escherichia coli | 379 | 379 | 99% | 5.00E-130 | 81.62 | 269 | WP_249524430.1 |
| 2-oxoglutarate dehydrogenase complex dihydrolipoyllysine-residue succinyltransferase [Escherichia coli] | Escherichia coli | 379 | 379 | 99% | 5.00E-130 | 81.62 | 280 | WP_320759799.1 |
| 2-oxoglutarate dehydrogenase complex dihydrolipoyllysine-residue succinyltransferase [Escherichia coli] | Escherichia coli | 379 | 379 | 99% | 5.00E-130 | 81.62 | 274 | RIC38964.1 |
| dihydrolipoamide succinyltransferase component (E2) [Escherichia coli] | Escherichia coli | 381 | 381 | 99% | 5.00E-130 | 81.62 | 332 | STM59242.1 |
| 2-oxoglutarate dehydrogenase complex dihydrolipoyllysine-residue succinyltransferase [Escherichia coli] | Escherichia coli | 380 | 380 | 99% | 5.00E-130 | 81.62 | 300 | MBA1840094.1 |
| 2-oxoglutarate dehydrogenase complex dihydrolipoyllysine-residue succinyltransferase [Escherichia coli] | Escherichia coli | 381 | 381 | 99% | 5.00E-130 | 81.62 | 338 | MDY8712251.1 |
| 2-oxoglutarate dehydrogenase complex dihydrolipoyllysine-residue succinyltransferase [Escherichia coli] | Escherichia coli | 384 | 384 | 99% | 6.00E-130 | 82.05 | 405 | EGX8818027.1 |
| 2-oxoglutarate dehydrogenase complex dihydrolipoyllysine-residue succinyltransferase [Escherichia coli] | Escherichia coli | 384 | 384 | 99% | 6.00E-130 | 82.05 | 405 | ENF3085891.1 |
| 2-oxoglutarate dehydrogenase complex dihydrolipoyllysine-residue succinyltransferase [Escherichia coli] | Escherichia coli | 379 | 379 | 99% | 6.00E-130 | 81.62 | 274 | MDY8964183.1 |
| 2-oxoglutarate dehydrogenase complex dihydrolipoyllysine-residue succinyltransferase [Escherichia coli] | Escherichia coli | 384 | 384 | 99% | 6.00E-130 | 82.05 | 405 | ENL2646046.1 |
| 2-oxoglutarate dehydrogenase complex dihydrolipoyllysine-residue succinyltransferase [Escherichia coli] | Escherichia coli | 379 | 379 | 99% | 6.00E-130 | 81.62 | 292 | WP_194498297.1 |
| 2-oxoglutarate dehydrogenase complex dihydrolipoyllysine-residue succinyltransferase [Escherichia coli] | Escherichia coli | 381 | 381 | 99% | 6.00E-130 | 81.62 | 337 | WP_048224949.1 |
| dihydrolipoyllysine-residue succinyltransferase [Escherichia coli] | Escherichia coli | 377 | 377 | 98% | 6.00E-130 | 81.55 | 230 | EGE7843444.1 |
| 2-oxoglutarate dehydrogenase complex dihydrolipoyllysine-residue succinyltransferase [Escherichia coli] | Escherichia coli | 383 | 383 | 99% | 7.00E-130 | 81.62 | 405 | EKM5464437.1 |
| TPA: 2-oxoglutarate dehydrogenase complex dihydrolipoyllysine-residue succinyltransferase [Escherichia coli] | Escherichia coli | 383 | 383 | 99% | 7.00E-130 | 82.05 | 405 | HAN9520759.1 |
| 2-oxoglutarate dehydrogenase complex dihydrolipoyllysine-residue succinyltransferase [Escherichia coli] | Escherichia coli | 381 | 381 | 99% | 7.00E-130 | 81.62 | 354 | MBE9780112.1 |
| 2-oxoglutarate dehydrogenase complex dihydrolipoyllysine-residue succinyltransferase [Escherichia coli] | Escherichia coli | 380 | 380 | 99% | 8.00E-130 | 81.62 | 328 | WP_229002757.1 |
| TPA: 2-oxoglutarate dehydrogenase complex dihydrolipoyllysine-residue succinyltransferase [Escherichia coli] | Escherichia coli | 383 | 383 | 99% | 8.00E-130 | 81.62 | 400 | HDI9351051.1 |
| 2-oxoglutarate dehydrogenase complex dihydrolipoyllysine-residue succinyltransferase [Escherichia coli] | Escherichia coli | 383 | 383 | 99% | 8.00E-130 | 82.05 | 405 | WP_112916033.1 |
| 2-oxoglutarate dehydrogenase complex dihydrolipoyllysine-residue succinyltransferase [Escherichia coli] | Escherichia coli | 383 | 383 | 99% | 8.00E-130 | 82.05 | 405 | WP_024234840.1 |
| 2-oxoglutarate dehydrogenase complex dihydrolipoyllysine-residue succinyltransferase [Escherichia coli] | Escherichia coli | 383 | 383 | 99% | 8.00E-130 | 82.05 | 405 | WP_302422103.1 |
| 2-oxoglutarate dehydrogenase complex dihydrolipoyllysine-residue succinyltransferase [Escherichia coli] | Escherichia coli | 381 | 381 | 99% | 9.00E-130 | 81.62 | 358 | WP_368270211.1 |
| TPA: 2-oxoglutarate dehydrogenase complex dihydrolipoyllysine-residue succinyltransferase [Escherichia coli] | Escherichia coli | 380 | 380 | 99% | 9.00E-130 | 81.62 | 332 | HCN8464961.1 |
| TPA: 2-oxoglutarate dehydrogenase complex dihydrolipoyllysine-residue succinyltransferase [Escherichia coli] | Escherichia coli | 383 | 383 | 99% | 9.00E-130 | 82.05 | 405 | HDS6732478.1 |
| dihydrolipoyllysine-residue succinyltransferase [Escherichia coli] | Escherichia coli | 383 | 383 | 99% | 9.00E-130 | 81.62 | 405 | EFO2789461.1 |
| TPA: 2-oxoglutarate dehydrogenase complex dihydrolipoyllysine-residue succinyltransferase [Escherichia coli] | Escherichia coli | 383 | 383 | 99% | 1.00E-129 | 81.62 | 405 | HEI3235152.1 |
| 2-oxoglutarate dehydrogenase complex dihydrolipoyllysine-residue succinyltransferase [Escherichia coli] | Escherichia coli | 380 | 380 | 99% | 1.00E-129 | 81.62 | 331 | WP_042068848.1 |
| TPA: 2-oxoglutarate dehydrogenase complex dihydrolipoyllysine-residue succinyltransferase [Escherichia coli] | Escherichia coli | 383 | 383 | 99% | 1.00E-129 | 81.62 | 405 | HDK8920924.1 |
| TPA: 2-oxoglutarate dehydrogenase complex dihydrolipoyllysine-residue succinyltransferase [Escherichia coli] | Escherichia coli | 382 | 382 | 99% | 1.00E-129 | 81.62 | 375 | HBB3835421.1 |
| 2-oxoglutarate dehydrogenase complex dihydrolipoyllysine-residue succinyltransferase [Escherichia coli] | Escherichia coli | 381 | 381 | 99% | 1.00E-129 | 81.62 | 366 | MXS49548.1 |
| 2-oxoglutarate dehydrogenase complex dihydrolipoyllysine-residue succinyltransferase [Escherichia coli] | Escherichia coli | 383 | 383 | 99% | 1.00E-129 | 81.62 | 405 | WP_103572461.1 |
| 2-oxoglutarate dehydrogenase complex dihydrolipoyllysine-residue succinyltransferase [Escherichia coli] | Escherichia coli | 380 | 380 | 99% | 1.00E-129 | 81.62 | 345 | MBA1060735.1 |

##

## Supplemental Table 9. Protein BLAST results based on consensus sequence of automated model building on a 4.00 Å low-pass filtered volume map

| **Description** | **Scientific Name** | **Max Score** | **Total Score** | **Query Cover** | **E value** | **Per. ident** | **Acc. Len** | **Accession** |
| --- | --- | --- | --- | --- | --- | --- | --- | --- |
| dihydrolipoyllysine-residue succinyltransferase [Escherichia coli] | Escherichia coli | 352 | 352 | 99% | 7.00E-120 | 73.62 | 239 | EEZ3479170.1 |
| dihydrolipoyllysine-residue succinyltransferase [Escherichia coli] | Escherichia coli | 352 | 352 | 99% | 7.00E-120 | 73.62 | 235 | WP_250378311.1 |
| dihydrolipoyllysine-residue succinyltransferase [Escherichia coli] | Escherichia coli | 352 | 352 | 99% | 7.00E-120 | 73.62 | 238 | EFO3983716.1 |
| dihydrolipoyllysine-residue succinyltransferase [Escherichia] | Escherichia | 352 | 352 | 99% | 7.00E-120 | 73.62 | 236 | WP_000629458.1 |
| TPA: dihydrolipoyllysine-residue succinyltransferase [Escherichia coli] | Escherichia coli | 352 | 352 | 99% | 8.00E-120 | 73.62 | 240 | HDY2775515.1 |
| dihydrolipoyllysine-residue succinyltransferase [Escherichia coli O157:H7] | Escherichia coli O157:H7 | 352 | 352 | 99% | 8.00E-120 | 73.62 | 240 | EFC3643281.1 |
| dihydrolipoyllysine-residue succinyltransferase [Escherichia coli] | Escherichia coli | 351 | 351 | 99% | 9.00E-120 | 73.62 | 234 | WP_149465242.1 |
| dihydrolipoyllysine-residue succinyltransferase [Escherichia coli] | Escherichia coli | 351 | 351 | 99% | 9.00E-120 | 73.62 | 233 | WP_136823828.1 |
| dihydrolipoyllysine-residue succinyltransferase [Escherichia coli] | Escherichia coli | 351 | 351 | 99% | 1.00E-119 | 73.62 | 237 | WP_252389303.1 |
| dihydrolipoyllysine-residue succinyltransferase [Escherichia coli] | Escherichia coli | 351 | 351 | 99% | 1.00E-119 | 73.62 | 241 | EIH9592508.1 |
| dihydrolipoyllysine-residue succinyltransferase [Escherichia coli] | Escherichia coli | 352 | 352 | 99% | 1.00E-119 | 73.62 | 243 | TJB60735.1 |
| dihydrolipoyllysine-residue succinyltransferase [Escherichia coli] | Escherichia coli | 351 | 351 | 98% | 1.00E-119 | 73.93 | 232 | ENT2450297.1 |
| dihydrolipoyllysine-residue succinyltransferase [Escherichia coli] | Escherichia coli | 351 | 351 | 99% | 1.00E-119 | 73.19 | 235 | WP_122792276.1 |
| dihydrolipoyllysine-residue succinyltransferase [Escherichia coli] | Escherichia coli | 351 | 351 | 98% | 2.00E-119 | 73.93 | 242 | WP_149518815.1 |
| dihydrolipoyllysine-residue succinyltransferase [Escherichia coli] | Escherichia coli | 351 | 351 | 98% | 2.00E-119 | 73.93 | 243 | EEX5691720.1 |
| dihydrolipoyllysine-residue succinyltransferase [Escherichia coli] | Escherichia coli | 351 | 351 | 98% | 2.00E-119 | 73.93 | 248 | KAA0647049.1 |
| TPA: dihydrolipoyllysine-residue succinyltransferase [Escherichia coli] | Escherichia coli | 351 | 351 | 98% | 2.00E-119 | 73.93 | 244 | HBB1186217.1 |
| dihydrolipoyllysine-residue succinyltransferase [Escherichia coli] | Escherichia coli | 352 | 352 | 98% | 2.00E-119 | 73.93 | 257 | MCU7719730.1 |
| dihydrolipoyllysine-residue succinyltransferase [Escherichia coli] | Escherichia coli | 351 | 351 | 98% | 2.00E-119 | 73.93 | 245 | WP_136775487.1 |
| dihydrolipoyllysine-residue succinyltransferase [Salmonella enterica subsp. enterica] | Salmonella enterica subsp. enterica | 351 | 351 | 98% | 2.00E-119 | 73.93 | 249 | EHK8207480.1 |
| dihydrolipoyllysine-residue succinyltransferase [Escherichia coli O145:H28] | Escherichia coli O145:H28 | 351 | 351 | 98% | 2.00E-119 | 73.93 | 246 | EJH5192981.1 |
| dihydrolipoyllysine-residue succinyltransferase [Escherichia coli] | Escherichia coli | 351 | 351 | 98% | 2.00E-119 | 73.93 | 247 | WP_185168965.1 |
| dihydrolipoyllysine-residue succinyltransferase [Escherichia coli] | Escherichia coli | 351 | 351 | 98% | 2.00E-119 | 73.93 | 259 | EGE6907964.1 |
| dihydrolipoyllysine-residue succinyltransferase [Escherichia coli] | Escherichia coli | 351 | 351 | 98% | 2.00E-119 | 73.93 | 259 | WP_119682542.1 |
| dihydrolipoyllysine-residue succinyltransferase [Escherichia coli] | Escherichia coli | 351 | 351 | 98% | 2.00E-119 | 73.93 | 248 | TJB30469.1 |
| dihydrolipoyllysine-residue succinyltransferase [Escherichia coli] | Escherichia coli | 351 | 351 | 98% | 2.00E-119 | 73.93 | 259 | EIC1734575.1 |
| dihydrolipoyllysine-residue succinyltransferase [Escherichia coli] | Escherichia coli | 351 | 351 | 98% | 2.00E-119 | 73.93 | 260 | WP_252391012.1 |
| TPA: dihydrolipoyllysine-residue succinyltransferase [Escherichia coli] | Escherichia coli | 351 | 351 | 98% | 2.00E-119 | 73.93 | 254 | HDC0239447.1 |
| dihydrolipoyllysine-residue succinyltransferase [Escherichia coli] | Escherichia coli | 351 | 351 | 98% | 2.00E-119 | 73.93 | 255 | WP_042066964.1 |
| dihydrolipoyllysine-residue succinyltransferase [Escherichia coli O145:H28] | Escherichia coli O145:H28 | 352 | 352 | 98% | 2.00E-119 | 73.93 | 264 | EJH5270977.1 |
| hypothetical protein ECZU26_29390 [Escherichia coli] | Escherichia coli | 351 | 351 | 98% | 2.00E-119 | 73.93 | 260 | GHL32114.1 |
| dihydrolipoyllysine-residue succinyltransferase [Escherichia coli] | Escherichia coli | 351 | 351 | 98% | 2.00E-119 | 73.93 | 250 | WP_323653019.1 |
| dihydrolipoamide succinyltransferase component (E2) [Escherichia coli] | Escherichia coli | 352 | 352 | 98% | 2.00E-119 | 73.93 | 269 | VFT70387.1 |
| dihydrolipoyllysine-residue succinyltransferase [Escherichia coli] | Escherichia coli | 351 | 351 | 98% | 2.00E-119 | 73.93 | 252 | MDY9032137.1 |
| TPA: dihydrolipoyllysine-residue succinyltransferase [Escherichia coli] | Escherichia coli | 351 | 351 | 98% | 2.00E-119 | 73.93 | 252 | HBD1696035.1 |
| dihydrolipoamide succinyltransferase component (E2) [Escherichia coli] | Escherichia coli | 352 | 352 | 98% | 2.00E-119 | 73.93 | 269 | STG69599.1 |
| dihydrolipoyllysine-residue succinyltransferase [Escherichia coli] | Escherichia coli | 351 | 351 | 98% | 2.00E-119 | 73.93 | 253 | TJT18798.1 |
| dihydrolipoyllysine-residue succinyltransferase [Escherichia coli] | Escherichia coli | 351 | 351 | 98% | 2.00E-119 | 73.93 | 253 | WP_136748022.1 |
| dihydrolipoyllysine-residue succinyltransferase [Escherichia coli] | Escherichia coli | 351 | 351 | 98% | 2.00E-119 | 73.93 | 253 | EFH6296858.1 |
| TPA: dihydrolipoyllysine-residue succinyltransferase [Escherichia coli] | Escherichia coli | 351 | 351 | 98% | 2.00E-119 | 73.93 | 261 | HAM4331889.1 |
| TPA: dihydrolipoyllysine-residue succinyltransferase [Escherichia coli] | Escherichia coli | 351 | 351 | 98% | 2.00E-119 | 73.93 | 265 | HAP0635211.1 |
| dihydrolipoyllysine-residue succinyltransferase [Escherichia coli] | Escherichia coli | 351 | 351 | 98% | 2.00E-119 | 73.93 | 256 | MCJ8642909.1 |
| 2-oxoglutarate dehydrogenase complex dihydrolipoyllysine-residue succinyltransferase [Escherichia coli] | Escherichia coli | 352 | 352 | 98% | 2.00E-119 | 73.93 | 269 | WP_115969540.1 |
| 2-oxoglutarate dehydrogenase complex dihydrolipoyllysine-residue succinyltransferase [Escherichia coli] | Escherichia coli | 352 | 352 | 98% | 2.00E-119 | 73.93 | 269 | WP_249524430.1 |
| 2-oxoglutarate dehydrogenase complex dihydrolipoyllysine-residue succinyltransferase [Escherichia coli] | Escherichia coli | 352 | 352 | 98% | 3.00E-119 | 73.93 | 282 | MDY9040892.1 |
| 2-oxoglutarate dehydrogenase complex dihydrolipoyllysine-residue succinyltransferase [Escherichia coli] | Escherichia coli | 351 | 351 | 98% | 3.00E-119 | 73.93 | 270 | WP_250382326.1 |
| 2-oxoglutarate dehydrogenase complex dihydrolipoyllysine-residue succinyltransferase [Escherichia coli] | Escherichia coli | 352 | 352 | 98% | 3.00E-119 | 73.93 | 280 | WP_320759799.1 |
| dihydrolipoyllysine-residue succinyltransferase [Escherichia coli] | Escherichia coli | 351 | 351 | 98% | 3.00E-119 | 73.93 | 263 | WP_047654718.1 |
| dihydrolipoamide succinyltransferase [Escherichia coli] | Escherichia coli | 351 | 351 | 98% | 3.00E-119 | 73.93 | 263 | OWF26202.1 |
| TPA: dihydrolipoyllysine-residue succinyltransferase [Escherichia coli] | Escherichia coli | 351 | 351 | 98% | 3.00E-119 | 73.93 | 264 | HAN7662089.1 |
| dihydrolipoamide succinyltransferase [Shigella sonnei] | Shigella sonnei | 352 | 352 | 98% | 3.00E-119 | 73.93 | 291 | CSF30971.1 |
| 2-oxoglutarate dehydrogenase complex dihydrolipoyllysine-residue succinyltransferase [Escherichia coli] | Escherichia coli | 351 | 351 | 98% | 3.00E-119 | 73.93 | 274 | MDY8964183.1 |
| 2-oxoglutarate dehydrogenase complex dihydrolipoyllysine-residue succinyltransferase [Escherichia coli] | Escherichia coli | 352 | 352 | 98% | 3.00E-119 | 73.93 | 287 | EEZ2060122.1 |
| 2-oxoglutarate dehydrogenase complex dihydrolipoyllysine-residue succinyltransferase [Escherichia coli] | Escherichia coli | 356 | 356 | 99% | 3.00E-119 | 74.04 | 405 | WP_136803128.1 |
| 2-oxoglutarate dehydrogenase complex dihydrolipoyllysine-residue succinyltransferase [Escherichia coli] | Escherichia coli | 356 | 356 | 99% | 3.00E-119 | 74.04 | 405 | WP_096845185.1 |
| dihydrolipoyllysine-residue succinyltransferase [Escherichia coli] | Escherichia coli | 350 | 350 | 98% | 3.00E-119 | 73.93 | 253 | WP_136801371.1 |
| 2-oxoglutarate dehydrogenase complex dihydrolipoyllysine-residue succinyltransferase [Escherichia coli] | Escherichia coli | 352 | 352 | 98% | 4.00E-119 | 73.93 | 286 | WP_060643349.1 |
| 2-oxoglutarate dehydrogenase complex dihydrolipoyllysine-residue succinyltransferase [Escherichia coli] | Escherichia coli | 351 | 351 | 98% | 4.00E-119 | 73.93 | 274 | RIC38964.1 |
| 2-oxoglutarate dehydrogenase complex dihydrolipoyllysine-residue succinyltransferase [Escherichia coli] | Escherichia coli | 351 | 351 | 98% | 4.00E-119 | 73.93 | 275 | WP_136750337.1 |
| TPA: 2-oxoglutarate dehydrogenase complex dihydrolipoyllysine-residue succinyltransferase [Escherichia coli] | Escherichia coli | 352 | 352 | 98% | 4.00E-119 | 73.93 | 312 | HCO0761733.1 |
| 2-oxoglutarate dehydrogenase complex dihydrolipoyllysine-residue succinyltransferase [Escherichia coli] | Escherichia coli | 352 | 352 | 98% | 4.00E-119 | 73.93 | 295 | NAG00763.1 |
| 2-oxoglutarate dehydrogenase complex dihydrolipoyllysine-residue succinyltransferase [Escherichia coli] | Escherichia coli | 356 | 356 | 99% | 5.00E-119 | 74.04 | 405 | MDY9786167.1 |
| 2-oxoglutarate dehydrogenase complex dihydrolipoyllysine-residue succinyltransferase [Escherichia coli] | Escherichia coli | 352 | 352 | 98% | 5.00E-119 | 73.93 | 292 | WP_194498297.1 |
| 2-oxoglutarate dehydrogenase complex dihydrolipoyllysine-residue succinyltransferase [Escherichia coli] | Escherichia coli | 352 | 352 | 98% | 5.00E-119 | 73.93 | 303 | EEY6784672.1 |
| 2-oxoglutarate dehydrogenase complex dihydrolipoyllysine-residue succinyltransferase [Escherichia coli] | Escherichia coli | 351 | 351 | 98% | 5.00E-119 | 73.93 | 286 | WP_047081876.1 |
| 2-oxoglutarate dehydrogenase complex dihydrolipoyllysine-residue succinyltransferase [Escherichia coli] | Escherichia coli | 353 | 353 | 98% | 5.00E-119 | 73.93 | 321 | EEY6693670.1 |
| 2-oxoglutarate dehydrogenase complex dihydrolipoyllysine-residue succinyltransferase [Escherichia coli] | Escherichia coli | 356 | 356 | 98% | 5.00E-119 | 73.93 | 405 | WP_192459758.1 |
| 2-oxoglutarate dehydrogenase complex dihydrolipoyllysine-residue succinyltransferase [Escherichia coli] | Escherichia coli | 353 | 353 | 98% | 5.00E-119 | 73.93 | 324 | EER8331471.1 |
| 2-oxoglutarate dehydrogenase complex dihydrolipoyllysine-residue succinyltransferase [Escherichia coli] | Escherichia coli | 353 | 353 | 98% | 5.00E-119 | 73.93 | 324 | MDO4358214.1 |
| TPA: dihydrolipoyllysine-residue succinyltransferase [Escherichia coli] | Escherichia coli | 350 | 350 | 99% | 5.00E-119 | 73.62 | 250 | HBI3672065.1 |
| 2-oxoglutarate dehydrogenase complex dihydrolipoyllysine-residue succinyltransferase [Escherichia coli] | Escherichia coli | 352 | 352 | 98% | 5.00E-119 | 73.93 | 300 | MBA1840094.1 |
| 2-oxoglutarate dehydrogenase complex dihydrolipoyllysine-residue succinyltransferase [Escherichia coli] | Escherichia coli | 353 | 353 | 98% | 5.00E-119 | 73.93 | 338 | MDY8712251.1 |
| dihydrolipoyllysine-residue succinyltransferase [Escherichia coli O157:H7] | Escherichia coli O157:H7 | 349 | 349 | 98% | 6.00E-119 | 73.82 | 231 | EEW1174705.1 |
| 2-oxoglutarate dehydrogenase complex dihydrolipoyllysine-residue succinyltransferase [Escherichia coli] | Escherichia coli | 352 | 352 | 98% | 6.00E-119 | 73.93 | 318 | MCX0506393.1 |
| dihydrolipoamide succinyltransferase component (E2) [Escherichia coli] | Escherichia coli | 353 | 353 | 98% | 6.00E-119 | 73.93 | 332 | STM59242.1 |
| 2-oxoglutarate dehydrogenase complex dihydrolipoyllysine-residue succinyltransferase [Escherichia coli] | Escherichia coli | 352 | 352 | 98% | 6.00E-119 | 73.93 | 322 | WP_194154148.1 |
| 2-oxoglutarate dehydrogenase complex dihydrolipoyllysine-residue succinyltransferase [Escherichia coli] | Escherichia coli | 352 | 352 | 98% | 6.00E-119 | 73.93 | 322 | MCU8601182.1 |
| 2-oxoglutarate dehydrogenase complex dihydrolipoyllysine-residue succinyltransferase [Escherichia coli] | Escherichia coli | 353 | 353 | 98% | 7.00E-119 | 73.93 | 328 | WP_229002757.1 |
| 2-oxoglutarate dehydrogenase complex dihydrolipoyllysine-residue succinyltransferase [Escherichia coli] | Escherichia coli | 355 | 355 | 98% | 7.00E-119 | 74.36 | 405 | MDF1405070.1 |
| 2-oxoglutarate dehydrogenase complex dihydrolipoyllysine-residue succinyltransferase [Escherichia coli] | Escherichia coli | 355 | 355 | 98% | 7.00E-119 | 74.36 | 405 | EEZ9068234.1 |
| TPA: 2-oxoglutarate dehydrogenase complex dihydrolipoyllysine-residue succinyltransferase [Escherichia coli] | Escherichia coli | 353 | 353 | 98% | 8.00E-119 | 73.93 | 332 | HCN8464961.1 |
| 2-oxoglutarate dehydrogenase complex dihydrolipoyllysine-residue succinyltransferase [Escherichia coli] | Escherichia coli | 353 | 353 | 98% | 8.00E-119 | 73.93 | 337 | WP_048224949.1 |
| 2-oxoglutarate dehydrogenase complex dihydrolipoyllysine-residue succinyltransferase [Escherichia coli] | Escherichia coli | 352 | 352 | 98% | 8.00E-119 | 73.93 | 331 | WP_042068848.1 |
| 2-oxoglutarate dehydrogenase complex dihydrolipoyllysine-residue succinyltransferase [Escherichia coli] | Escherichia coli | 353 | 353 | 98% | 8.00E-119 | 73.93 | 345 | MBA1060735.1 |
| 2-oxoglutarate dehydrogenase complex dihydrolipoyllysine-residue succinyltransferase [Escherichia coli] | Escherichia coli | 353 | 353 | 98% | 9.00E-119 | 73.93 | 354 | MBE9780112.1 |
| 2-oxoglutarate dehydrogenase complex dihydrolipoyllysine-residue succinyltransferase [Escherichia coli] | Escherichia coli | 355 | 355 | 98% | 9.00E-119 | 73.93 | 405 | EKM5464437.1 |
| 2-oxoglutarate dehydrogenase complex dihydrolipoyllysine-residue succinyltransferase [Escherichia coli] | Escherichia coli | 355 | 355 | 98% | 1.00E-118 | 74.36 | 405 | WP_032210407.1 |
| TPA: 2-oxoglutarate dehydrogenase complex dihydrolipoyllysine-residue succinyltransferase [Escherichia coli] | Escherichia coli | 355 | 355 | 98% | 1.00E-118 | 74.36 | 405 | HAH8524627.1 |
| dihydrolipoyllysine-residue succinyltransferase [Escherichia coli] | Escherichia coli | 349 | 349 | 98% | 1.00E-118 | 73.93 | 253 | WP_347079825.1 |
| 2-oxoglutarate dehydrogenase complex dihydrolipoyllysine-residue succinyltransferase [Escherichia coli] | Escherichia coli | 355 | 355 | 98% | 1.00E-118 | 74.36 | 405 | EIR6544570.1 |
| TPA: 2-oxoglutarate dehydrogenase complex dihydrolipoyllysine-residue succinyltransferase [Escherichia coli] | Escherichia coli | 355 | 355 | 98% | 1.00E-118 | 73.93 | 405 | HCP8029924.1 |
| 2-oxoglutarate dehydrogenase complex dihydrolipoyllysine-residue succinyltransferase [Escherichia] | Escherichia | 355 | 355 | 98% | 1.00E-118 | 74.36 | 405 | WP_000099817.1 |
| 2-oxoglutarate dehydrogenase complex dihydrolipoyllysine-residue succinyltransferase [Escherichia coli] | Escherichia coli | 350 | 350 | 98% | 1.00E-118 | 73.93 | 279 | WP_247166683.1 |
| 2-oxoglutarate dehydrogenase complex dihydrolipoyllysine-residue succinyltransferase [Escherichia coli] | Escherichia coli | 355 | 355 | 99% | 1.00E-118 | 73.31 | 405 | UIU60256.1 |
| TPA: 2-oxoglutarate dehydrogenase complex dihydrolipoyllysine-residue succinyltransferase [Escherichia coli] | Escherichia coli | 355 | 355 | 98% | 1.00E-118 | 74.36 | 405 | HCT2454644.1 |
| 2-oxoglutarate dehydrogenase complex dihydrolipoyllysine-residue succinyltransferase [Escherichia coli] | Escherichia coli | 353 | 353 | 98% | 1.00E-118 | 73.93 | 366 | MXS49548.1 |
| 2-oxoglutarate dehydrogenase complex dihydrolipoyllysine-residue succinyltransferase [Escherichia albertii] | Escherichia albertii | 355 | 355 | 99% | 1.00E-118 | 73.62 | 400 | WP_131109862.1 |
| 2-oxoglutarate dehydrogenase complex dihydrolipoyllysine-residue succinyltransferase [Escherichia coli] | Escherichia coli | 355 | 355 | 98% | 1.00E-118 | 74.36 | 405 | ENL2646046.1 |
| 2-oxoglutarate dehydrogenase complex dihydrolipoyllysine-residue succinyltransferase [Escherichia coli] | Escherichia coli | 355 | 355 | 98% | 1.00E-118 | 74.36 | 405 | WP_112916033.1 |
| TPA: 2-oxoglutarate dehydrogenase complex dihydrolipoyllysine-residue succinyltransferase [Escherichia coli] | Escherichia coli | 355 | 355 | 98% | 1.00E-118 | 74.36 | 405 | HAN9520759.1 |

##

## Supplemental Table 10. Protein BLAST results based on consensus sequence of automated model building on a 4.25 Å low-pass filtered volume map

| **Description** | **Scientific Name** | **Max Score** | **Total Score** | **Query Cover** | **E value** | **Per. ident** | **Acc. Len** | **Accession** |
| --- | --- | --- | --- | --- | --- | --- | --- | --- |
| dihydrolipoyllysine-residue succinyltransferase [Escherichia coli] | Escherichia coli | 305 | 305 | 95% | 1.00E-101 | 66.08 | 226 | WP_074164196.1 |
| dihydrolipoyllysine-residue succinyltransferase [Escherichia coli] | Escherichia coli | 304 | 304 | 99% | 4.00E-101 | 64.68 | 239 | EEZ3479170.1 |
| dihydrolipoyllysine-residue succinyltransferase [Escherichia] | Escherichia | 304 | 304 | 99% | 4.00E-101 | 64.68 | 236 | WP_000629458.1 |
| TPA: dihydrolipoyllysine-residue succinyltransferase [Escherichia coli] | Escherichia coli | 304 | 304 | 99% | 5.00E-101 | 64.68 | 240 | HDY2775515.1 |
| dihydrolipoyllysine-residue succinyltransferase [Escherichia coli O157:H7] | Escherichia coli O157:H7 | 304 | 304 | 99% | 5.00E-101 | 64.68 | 240 | EFC3643281.1 |
| dihydrolipoyllysine-residue succinyltransferase [Escherichia coli] | Escherichia coli | 304 | 304 | 99% | 5.00E-101 | 64.68 | 238 | EFO3983716.1 |
| dihydrolipoyllysine-residue succinyltransferase [Escherichia coli] | Escherichia coli | 304 | 304 | 99% | 5.00E-101 | 64.68 | 243 | TJB60735.1 |
| dihydrolipoyllysine-residue succinyltransferase [Escherichia coli] | Escherichia coli | 304 | 304 | 99% | 6.00E-101 | 64.68 | 237 | WP_252389303.1 |
| dihydrolipoyllysine-residue succinyltransferase [Escherichia coli] | Escherichia coli | 304 | 304 | 99% | 6.00E-101 | 64.68 | 241 | EIH9592508.1 |
| dihydrolipoyllysine-residue succinyltransferase [Escherichia coli] | Escherichia coli | 303 | 303 | 99% | 7.00E-101 | 64.68 | 235 | WP_250378311.1 |
| dihydrolipoyllysine-residue succinyltransferase [Escherichia coli] | Escherichia coli | 303 | 303 | 98% | 7.00E-101 | 64.96 | 232 | ENT2450297.1 |
| dihydrolipoyllysine-residue succinyltransferase [Escherichia coli] | Escherichia coli | 303 | 303 | 99% | 7.00E-101 | 64.68 | 234 | WP_149465242.1 |
| dihydrolipoyllysine-residue succinyltransferase [Escherichia coli] | Escherichia coli | 303 | 303 | 99% | 8.00E-101 | 64.68 | 233 | WP_136823828.1 |
| dihydrolipoyllysine-residue succinyltransferase [Escherichia coli O157:H7] | Escherichia coli O157:H7 | 303 | 303 | 97% | 9.00E-101 | 64.66 | 231 | EEW1174705.1 |
| dihydrolipoyllysine-residue succinyltransferase [Escherichia coli] | Escherichia coli | 303 | 303 | 99% | 9.00E-101 | 64.68 | 235 | WP_122792276.1 |
| dihydrolipoyllysine-residue succinyltransferase [Escherichia coli] | Escherichia coli | 303 | 303 | 98% | 1.00E-100 | 64.96 | 242 | WP_149518815.1 |
| dihydrolipoyllysine-residue succinyltransferase [Escherichia coli] | Escherichia coli | 303 | 303 | 98% | 1.00E-100 | 64.96 | 243 | EEX5691720.1 |
| TPA: dihydrolipoyllysine-residue succinyltransferase [Escherichia coli] | Escherichia coli | 303 | 303 | 98% | 1.00E-100 | 64.96 | 244 | HBB1186217.1 |
| dihydrolipoyllysine-residue succinyltransferase [Escherichia coli] | Escherichia coli | 303 | 303 | 98% | 1.00E-100 | 64.96 | 245 | WP_136775487.1 |
| dihydrolipoyllysine-residue succinyltransferase [Escherichia coli O145:H28] | Escherichia coli O145:H28 | 303 | 303 | 98% | 1.00E-100 | 64.96 | 246 | EJH5192981.1 |
| hypothetical protein ECZU22_19390 [Escherichia coli] | Escherichia coli | 303 | 303 | 95% | 1.00E-100 | 65.64 | 227 | GHL08040.1 |
| dihydrolipoyllysine-residue succinyltransferase [Escherichia coli] | Escherichia coli | 304 | 304 | 98% | 1.00E-100 | 64.96 | 257 | MCU7719730.1 |
| dihydrolipoyllysine-residue succinyltransferase [Escherichia coli] | Escherichia coli | 303 | 303 | 98% | 1.00E-100 | 64.96 | 247 | WP_185168965.1 |
| dihydrolipoyllysine-residue succinyltransferase [Escherichia coli] | Escherichia coli | 303 | 303 | 95% | 1.00E-100 | 65.64 | 226 | EER2248380.1 |
| dihydrolipoyllysine-residue succinyltransferase [Escherichia coli] | Escherichia coli | 303 | 303 | 98% | 1.00E-100 | 64.96 | 256 | MCJ8642909.1 |
| dihydrolipoyllysine-residue succinyltransferase [Escherichia coli] | Escherichia coli | 303 | 303 | 98% | 1.00E-100 | 64.96 | 248 | TJB30469.1 |
| dihydrolipoyllysine-residue succinyltransferase [Escherichia coli] | Escherichia coli | 303 | 303 | 98% | 1.00E-100 | 64.96 | 250 | WP_323653019.1 |
| dihydrolipoyllysine-residue succinyltransferase [Escherichia coli] | Escherichia coli | 303 | 303 | 97% | 1.00E-100 | 64.94 | 230 | EGE7843444.1 |
| dihydrolipoyllysine-residue succinyltransferase [Escherichia coli] | Escherichia coli | 302 | 302 | 95% | 1.00E-100 | 65.64 | 227 | EIH2436553.1 |
| dihydrolipoyllysine-residue succinyltransferase [Escherichia coli] | Escherichia coli | 303 | 303 | 98% | 1.00E-100 | 64.96 | 248 | KAA0647049.1 |
| dihydrolipoyllysine-residue succinyltransferase [Escherichia coli] | Escherichia coli | 302 | 302 | 95% | 1.00E-100 | 65.64 | 228 | WP_229024141.1 |
| dihydrolipoyllysine-residue succinyltransferase [Escherichia coli] | Escherichia coli | 303 | 303 | 98% | 1.00E-100 | 64.96 | 259 | EIC1734575.1 |
| dihydrolipoyllysine-residue succinyltransferase [Escherichia coli] | Escherichia coli | 303 | 303 | 98% | 1.00E-100 | 64.96 | 252 | MDY9032137.1 |
| TPA: dihydrolipoyllysine-residue succinyltransferase [Escherichia coli] | Escherichia coli | 303 | 303 | 98% | 1.00E-100 | 64.96 | 252 | HBD1696035.1 |
| dihydrolipoyllysine-residue succinyltransferase [Salmonella enterica subsp. enterica] | Salmonella enterica subsp. enterica | 303 | 303 | 98% | 1.00E-100 | 64.96 | 249 | EHK8207480.1 |
| dihydrolipoyllysine-residue succinyltransferase [Escherichia coli] | Escherichia coli | 304 | 304 | 98% | 1.00E-100 | 64.96 | 263 | WP_047654718.1 |
| dihydrolipoamide succinyltransferase [Escherichia coli] | Escherichia coli | 304 | 304 | 98% | 1.00E-100 | 64.96 | 263 | OWF26202.1 |
| dihydrolipoyllysine-residue succinyltransferase [Escherichia coli] | Escherichia coli | 303 | 303 | 98% | 1.00E-100 | 64.96 | 253 | TJT18798.1 |
| dihydrolipoyllysine-residue succinyltransferase [Escherichia coli] | Escherichia coli | 303 | 303 | 98% | 1.00E-100 | 64.96 | 253 | WP_136748022.1 |
| dihydrolipoyllysine-residue succinyltransferase [Escherichia coli] | Escherichia coli | 303 | 303 | 98% | 1.00E-100 | 64.96 | 253 | EFH6296858.1 |
| dihydrolipoyllysine-residue succinyltransferase [Escherichia coli O177] | Escherichia coli O177 | 302 | 302 | 95% | 1.00E-100 | 65.64 | 229 | EFA8854754.1 |
| dihydrolipoyllysine-residue succinyltransferase [Klebsiella pneumoniae] | Klebsiella pneumoniae | 303 | 303 | 99% | 1.00E-100 | 63.83 | 236 | MDN7188851.1 |
| dihydrolipoyllysine-residue succinyltransferase [Escherichia coli] | Escherichia coli | 303 | 303 | 98% | 2.00E-100 | 64.96 | 253 | WP_136801371.1 |
| dihydrolipoyllysine-residue succinyltransferase [Escherichia coli] | Escherichia coli | 303 | 303 | 98% | 2.00E-100 | 64.96 | 255 | WP_042066964.1 |
| TPA: dihydrolipoyllysine-residue succinyltransferase [Escherichia coli] | Escherichia coli | 303 | 303 | 98% | 2.00E-100 | 64.96 | 264 | HAN7662089.1 |
| dihydrolipoyllysine-residue succinyltransferase [Salmonella enterica subsp. enterica serovar Virginia] | Salmonella enterica subsp. enterica serovar Virginia | 303 | 303 | 99% | 2.00E-100 | 64.26 | 237 | MEA7606579.1 |
| hypothetical protein ECZU26_29390 [Escherichia coli] | Escherichia coli | 303 | 303 | 98% | 2.00E-100 | 64.96 | 260 | GHL32114.1 |
| dihydrolipoyllysine-residue succinyltransferase [Escherichia coli] | Escherichia coli | 303 | 303 | 98% | 2.00E-100 | 64.96 | 260 | WP_252391012.1 |
| TPA: dihydrolipoyllysine-residue succinyltransferase [Escherichia coli] | Escherichia coli | 303 | 303 | 98% | 2.00E-100 | 64.96 | 254 | HDC0239447.1 |
| dihydrolipoyllysine-residue succinyltransferase [Klebsiella pneumoniae] | Klebsiella pneumoniae | 303 | 303 | 99% | 2.00E-100 | 63.83 | 242 | WP_226956088.1 |
| TPA: dihydrolipoyllysine-residue succinyltransferase [Escherichia coli] | Escherichia coli | 303 | 303 | 98% | 2.00E-100 | 64.96 | 265 | HAP0635211.1 |
| dihydrolipoyllysine-residue succinyltransferase [Escherichia coli] | Escherichia coli | 303 | 303 | 98% | 2.00E-100 | 64.96 | 259 | EGE6907964.1 |
| dihydrolipoyllysine-residue succinyltransferase [Escherichia coli] | Escherichia coli | 303 | 303 | 98% | 2.00E-100 | 64.96 | 259 | WP_119682542.1 |
| dihydrolipoyllysine-residue succinyltransferase [Escherichia coli O145:H28] | Escherichia coli O145:H28 | 303 | 303 | 98% | 2.00E-100 | 64.96 | 264 | EJH5270977.1 |
| dihydrolipoyllysine-residue succinyltransferase [Klebsiella pneumoniae] | Klebsiella pneumoniae | 303 | 303 | 99% | 2.00E-100 | 63.83 | 241 | WP_002895034.1 |
| dihydrolipoyllysine-residue succinyltransferase [Klebsiella pneumoniae] | Klebsiella pneumoniae | 303 | 303 | 99% | 2.00E-100 | 63.83 | 246 | MBD7460289.1 |
| dihydrolipoamide succinyltransferase [Klebsiella pneumoniae] | Klebsiella pneumoniae | 302 | 302 | 99% | 2.00E-100 | 63.83 | 238 | KTG74015.1 |
| dihydrolipoyllysine-residue succinyltransferase [Escherichia coli] | Escherichia coli | 302 | 302 | 95% | 2.00E-100 | 65.64 | 227 | ENU3117923.1 |
| dihydrolipoyllysine-residue succinyltransferase [Klebsiella pneumoniae] | Klebsiella pneumoniae | 303 | 303 | 99% | 2.00E-100 | 63.83 | 244 | WP_017879867.1 |
| dihydrolipoyllysine-residue succinyltransferase [Klebsiella pneumoniae] | Klebsiella pneumoniae | 303 | 303 | 99% | 2.00E-100 | 63.83 | 245 | WP_077253825.1 |
| dihydrolipoyllysine-residue succinyltransferase [Klebsiella pneumoniae] | Klebsiella pneumoniae | 303 | 303 | 99% | 2.00E-100 | 63.83 | 243 | EIW9172619.1 |
| dihydrolipoyllysine-residue succinyltransferase [Klebsiella pneumoniae] | Klebsiella pneumoniae | 303 | 303 | 99% | 2.00E-100 | 63.83 | 246 | WP_096660644.1 |
| dihydrolipoyllysine-residue succinyltransferase [Klebsiella pneumoniae] | Klebsiella pneumoniae | 302 | 302 | 99% | 2.00E-100 | 63.83 | 237 | WP_151370611.1 |
| dihydrolipoyllysine-residue succinyltransferase [Klebsiella pneumoniae] | Klebsiella pneumoniae | 303 | 303 | 99% | 2.00E-100 | 63.83 | 247 | WP_151368547.1 |
| 2-oxoglutarate dehydrogenase complex dihydrolipoyllysine-residue succinyltransferase [Escherichia coli] | Escherichia coli | 304 | 304 | 98% | 2.00E-100 | 64.96 | 282 | MDY9040892.1 |
| dihydrolipoyllysine-residue succinyltransferase [Klebsiella pneumoniae] | Klebsiella pneumoniae | 303 | 303 | 99% | 2.00E-100 | 63.83 | 244 | PTB11879.1 |
| TPA: dihydrolipoyllysine-residue succinyltransferase [Klebsiella pneumoniae] | Klebsiella pneumoniae | 303 | 303 | 99% | 2.00E-100 | 63.83 | 248 | HDG8033015.1 |
| TPA: dihydrolipoyllysine-residue succinyltransferase [Klebsiella pneumoniae] | Klebsiella pneumoniae | 303 | 303 | 99% | 2.00E-100 | 63.83 | 248 | HBT5880924.1 |
| TPA: dihydrolipoyllysine-residue succinyltransferase [Klebsiella quasipneumoniae subsp. similipneumoniae] | Klebsiella quasipneumoniae subsp. similipneumoniae | 303 | 303 | 99% | 2.00E-100 | 63.83 | 245 | HBT4794623.1 |
| dihydrolipoyllysine-residue succinyltransferase [Klebsiella pneumoniae] | Klebsiella pneumoniae | 303 | 303 | 99% | 2.00E-100 | 63.83 | 249 | PCR33351.1 |
| dihydrolipoyllysine-residue succinyltransferase [Klebsiella pneumoniae] | Klebsiella pneumoniae | 302 | 302 | 99% | 2.00E-100 | 63.83 | 235 | WP_134933365.1 |
| dihydrolipoyllysine-residue succinyltransferase [Salmonella enterica subsp. enterica serovar Reading] | Salmonella enterica subsp. enterica serovar Reading | 302 | 302 | 99% | 2.00E-100 | 64.26 | 241 | EDV1620825.1 |
| 2-oxoglutarate dehydrogenase complex dihydrolipoyllysine-residue succinyltransferase [Escherichia coli] | Escherichia coli | 304 | 304 | 98% | 2.00E-100 | 64.96 | 280 | WP_320759799.1 |
| dihydrolipoamide succinyltransferase component (E2) [Escherichia coli] | Escherichia coli | 303 | 303 | 98% | 2.00E-100 | 64.96 | 269 | STG69599.1 |
| dihydrolipoyllysine-residue succinyltransferase [Klebsiella pneumoniae] | Klebsiella pneumoniae | 303 | 303 | 99% | 2.00E-100 | 63.83 | 250 | WP_097405167.1 |
| TPA: 2-oxoglutarate dehydrogenase complex dihydrolipoyllysine-residue succinyltransferase [Escherichia coli] | Escherichia coli | 308 | 308 | 98% | 2.00E-100 | 65.38 | 405 | HFG3098669.1 |
| TPA: dihydrolipoyllysine-residue succinyltransferase [Escherichia coli] | Escherichia coli | 303 | 303 | 98% | 2.00E-100 | 64.96 | 261 | HAM4331889.1 |
| 2-oxoglutarate dehydrogenase complex dihydrolipoyllysine-residue succinyltransferase [Escherichia coli] | Escherichia coli | 303 | 303 | 98% | 2.00E-100 | 64.96 | 274 | RIC38964.1 |
| 2-oxoglutarate dehydrogenase complex dihydrolipoyllysine-residue succinyltransferase [Escherichia coli] | Escherichia coli | 303 | 303 | 98% | 2.00E-100 | 64.96 | 275 | WP_136750337.1 |
| dihydrolipoamide succinyltransferase component (E2) [Escherichia coli] | Escherichia coli | 303 | 303 | 98% | 3.00E-100 | 64.96 | 269 | VFT70387.1 |
| TPA: dihydrolipoyllysine-residue succinyltransferase [Klebsiella pneumoniae] | Klebsiella pneumoniae | 303 | 303 | 99% | 3.00E-100 | 63.83 | 252 | HCD8671197.1 |
| 2-oxoglutarate dehydrogenase complex dihydrolipoyllysine-residue succinyltransferase [Escherichia coli] | Escherichia coli | 304 | 304 | 98% | 3.00E-100 | 64.96 | 286 | WP_060643349.1 |
| dihydrolipoyllysine-residue succinyltransferase [Klebsiella pneumoniae] | Klebsiella pneumoniae | 302 | 302 | 99% | 3.00E-100 | 63.83 | 244 | WP_129312831.1 |
| dihydrolipoyllysine-residue succinyltransferase [Klebsiella pneumoniae] | Klebsiella pneumoniae | 302 | 302 | 99% | 3.00E-100 | 63.83 | 239 | RCA06870.1 |
| dihydrolipoyllysine-residue succinyltransferase [Klebsiella pneumoniae] | Klebsiella pneumoniae | 303 | 303 | 99% | 3.00E-100 | 63.83 | 249 | WP_134921021.1 |
| 2-oxoglutarate dehydrogenase complex dihydrolipoyllysine-residue succinyltransferase [Escherichia coli] | Escherichia coli | 304 | 304 | 98% | 3.00E-100 | 64.96 | 286 | WP_047081876.1 |
| dihydrolipoyllysine-residue succinyltransferase [Klebsiella pneumoniae] | Klebsiella pneumoniae | 303 | 303 | 99% | 3.00E-100 | 63.83 | 253 | WP_265509381.1 |
| dihydrolipoyllysine-residue succinyltransferase [Klebsiella pneumoniae] | Klebsiella pneumoniae | 302 | 302 | 99% | 3.00E-100 | 63.83 | 249 | NEZ34953.1 |
| dihydrolipoyllysine-residue succinyltransferase [Klebsiella pneumoniae] | Klebsiella pneumoniae | 303 | 303 | 99% | 3.00E-100 | 63.83 | 251 | RYI69693.1 |
| dihydrolipoyllysine-residue succinyltransferase [Klebsiella pneumoniae] | Klebsiella pneumoniae | 303 | 303 | 99% | 3.00E-100 | 63.83 | 252 | RYI67680.1 |
| dihydrolipoyllysine-residue succinyltransferase [Klebsiella pneumoniae] | Klebsiella pneumoniae | 302 | 302 | 99% | 3.00E-100 | 63.83 | 240 | RCA07230.1 |
| 2-oxoglutarate dehydrogenase complex dihydrolipoyllysine-residue succinyltransferase [Escherichia coli] | Escherichia coli | 303 | 303 | 98% | 3.00E-100 | 64.96 | 270 | WP_250382326.1 |
| dihydrolipoyllysine-residue succinyltransferase [Klebsiella pneumoniae] | Klebsiella pneumoniae | 302 | 302 | 99% | 3.00E-100 | 63.83 | 243 | RYI67076.1 |
| dihydrolipoyllysine-residue succinyltransferase [Klebsiella pneumoniae] | Klebsiella pneumoniae | 303 | 303 | 99% | 3.00E-100 | 63.83 | 254 | WP_228986048.1 |
| dihydrolipoyllysine-residue succinyltransferase [Klebsiella pneumoniae] | Klebsiella pneumoniae | 302 | 302 | 99% | 3.00E-100 | 63.83 | 234 | WP_197324887.1 |
| TPA: dihydrolipoyllysine-residue succinyltransferase [Klebsiella pneumoniae] | Klebsiella pneumoniae | 303 | 303 | 99% | 3.00E-100 | 63.83 | 255 | HBY0075428.1 |
| dihydrolipoyllysine-residue succinyltransferase [Salmonella enterica] | Salmonella enterica | 302 | 302 | 99% | 3.00E-100 | 64.26 | 241 | EEN7692697.1 |
| 2-oxoglutarate dehydrogenase complex dihydrolipoyllysine-residue succinyltransferase [Escherichia coli] | Escherichia coli | 304 | 304 | 98% | 3.00E-100 | 64.96 | 287 | EEZ2060122.1 |
| TPA: 2-oxoglutarate dehydrogenase complex dihydrolipoyllysine-residue succinyltransferase [Escherichia coli] | Escherichia coli | 305 | 305 | 98% | 3.00E-100 | 64.96 | 312 | HCO0761733.1 |
| 2-oxoglutarate dehydrogenase complex dihydrolipoyllysine-residue succinyltransferase [Escherichia coli] | Escherichia coli | 304 | 304 | 98% | 3.00E-100 | 64.96 | 292 | WP_194498297.1 |

##

## Supplemental Table 11. Protein BLAST results based on consensus sequence of automated model building on a 4.50 Å low-pass filtered volume map

| **Description** | **Scientific Name** | **Max Score** | **Total Score** | **Query Cover** | **E value** | **Per. ident** | **Acc. Len** | **Accession** |
| --- | --- | --- | --- | --- | --- | --- | --- | --- |
| dihydrolipoamide acyltransferase SucB [Shewanella livingstonensis] | Shewanella livingstonensis | 162 | 162 | 87% | 5.00E-45 | 52.73 | 333 | BAL45917.1 |
| dihydrolipoyllysine-residue succinyltransferase [Enterobacter hormaechei] | Enterobacter hormaechei | 133 | 218 | 97% | 2.00E-34 | 46.15 | 256 | KAB2506993.1 |
| dihydrolipoyllysine-residue succinyltransferase [Escherichia coli] | Escherichia coli | 132 | 132 | 97% | 2.00E-34 | 46.15 | 218 | MCJ8727037.1 |
| dihydrolipoyllysine-residue succinyltransferase [Escherichia coli] | Escherichia coli | 132 | 132 | 97% | 4.00E-34 | 46.15 | 234 | WP_149465242.1 |
| dihydrolipoyllysine-residue succinyltransferase [Escherichia coli] | Escherichia coli | 131 | 131 | 97% | 4.00E-34 | 46.15 | 233 | WP_136823828.1 |
| dihydrolipoyllysine-residue succinyltransferase [Escherichia coli] | Escherichia coli | 131 | 131 | 97% | 4.00E-34 | 46.15 | 235 | WP_250378311.1 |
| dihydrolipoyllysine-residue succinyltransferase [Escherichia] | Escherichia | 131 | 131 | 97% | 4.00E-34 | 46.15 | 236 | WP_000629458.1 |
| dihydrolipoyllysine-residue succinyltransferase [Escherichia coli O157:H7] | Escherichia coli O157:H7 | 131 | 131 | 97% | 5.00E-34 | 46.15 | 231 | EEW1174705.1 |
| dihydrolipoyllysine-residue succinyltransferase [Escherichia coli] | Escherichia coli | 131 | 131 | 97% | 5.00E-34 | 46.15 | 232 | ENT2450297.1 |
| dihydrolipoyllysine-residue succinyltransferase [Escherichia coli] | Escherichia coli | 131 | 131 | 97% | 5.00E-34 | 46.15 | 237 | WP_252389303.1 |
| TPA: dihydrolipoyllysine-residue succinyltransferase [Escherichia coli] | Escherichia coli | 131 | 131 | 97% | 5.00E-34 | 46.15 | 240 | HDY2775515.1 |
| dihydrolipoyllysine-residue succinyltransferase [Escherichia coli O157:H7] | Escherichia coli O157:H7 | 131 | 131 | 97% | 5.00E-34 | 46.15 | 240 | EFC3643281.1 |
| dihydrolipoyllysine-residue succinyltransferase [Escherichia coli] | Escherichia coli | 131 | 131 | 97% | 5.00E-34 | 46.15 | 241 | EIH9592508.1 |
| dihydrolipoyllysine-residue succinyltransferase [Escherichia coli] | Escherichia coli | 131 | 131 | 97% | 5.00E-34 | 46.15 | 238 | EFO3983716.1 |
| dihydrolipoyllysine-residue succinyltransferase [Escherichia coli] | Escherichia coli | 131 | 131 | 97% | 5.00E-34 | 46.15 | 239 | EEZ3479170.1 |
| dihydrolipoyllysine-residue succinyltransferase [Escherichia coli] | Escherichia coli | 131 | 131 | 97% | 6.00E-34 | 46.15 | 235 | WP_122792276.1 |
| dihydrolipoyllysine-residue succinyltransferase [Escherichia coli] | Escherichia coli | 132 | 132 | 97% | 6.00E-34 | 46.15 | 248 | KAA0647049.1 |
| dihydrolipoyllysine-residue succinyltransferase [Escherichia coli] | Escherichia coli | 131 | 131 | 97% | 6.00E-34 | 46.15 | 243 | TJB60735.1 |
| dihydrolipoyllysine-residue succinyltransferase [Escherichia coli] | Escherichia coli | 131 | 131 | 97% | 6.00E-34 | 46.15 | 242 | WP_149518815.1 |
| dihydrolipoyllysine-residue succinyltransferase [Escherichia coli] | Escherichia coli | 131 | 131 | 97% | 6.00E-34 | 46.15 | 243 | EEX5691720.1 |
| TPA: dihydrolipoyllysine-residue succinyltransferase [Escherichia coli] | Escherichia coli | 131 | 131 | 97% | 6.00E-34 | 46.15 | 244 | HBB1186217.1 |
| dihydrolipoyllysine-residue succinyltransferase [Escherichia coli] | Escherichia coli | 131 | 131 | 97% | 6.00E-34 | 46.15 | 245 | WP_136775487.1 |
| dihydrolipoyllysine-residue succinyltransferase [Escherichia coli O145:H28] | Escherichia coli O145:H28 | 131 | 131 | 97% | 7.00E-34 | 46.15 | 246 | EJH5192981.1 |
| dihydrolipoyllysine-residue succinyltransferase [Escherichia coli] | Escherichia coli | 131 | 131 | 97% | 7.00E-34 | 46.15 | 247 | WP_185168965.1 |
| dihydrolipoyllysine-residue succinyltransferase [Escherichia coli] | Escherichia coli | 131 | 131 | 97% | 7.00E-34 | 46.15 | 248 | TJB30469.1 |
| dihydrolipoyllysine-residue succinyltransferase [Escherichia coli] | Escherichia coli | 132 | 132 | 97% | 7.00E-34 | 46.15 | 256 | MCJ8642909.1 |
| dihydrolipoyllysine-residue succinyltransferase [Salmonella enterica subsp. enterica] | Salmonella enterica subsp. enterica | 131 | 131 | 97% | 7.00E-34 | 46.15 | 249 | EHK8207480.1 |
| dihydrolipoyllysine-residue succinyltransferase [Escherichia coli] | Escherichia coli | 131 | 131 | 97% | 7.00E-34 | 46.15 | 250 | WP_323653019.1 |
| dihydrolipoyllysine-residue succinyltransferase [Escherichia coli] | Escherichia coli | 131 | 131 | 97% | 8.00E-34 | 46.15 | 255 | WP_042066964.1 |
| dihydrolipoyllysine-residue succinyltransferase [Escherichia coli] | Escherichia coli | 131 | 131 | 97% | 8.00E-34 | 46.15 | 252 | MDY9032137.1 |
| TPA: dihydrolipoyllysine-residue succinyltransferase [Escherichia coli] | Escherichia coli | 131 | 131 | 97% | 8.00E-34 | 46.15 | 252 | HBD1696035.1 |
| TPA: dihydrolipoyllysine-residue succinyltransferase [Escherichia coli] | Escherichia coli | 131 | 131 | 97% | 8.00E-34 | 46.15 | 250 | HBI3672065.1 |
| dihydrolipoyllysine-residue succinyltransferase [Escherichia coli] | Escherichia coli | 131 | 131 | 97% | 8.00E-34 | 46.15 | 253 | WP_347079825.1 |
| dihydrolipoyllysine-residue succinyltransferase [Escherichia coli] | Escherichia coli | 131 | 131 | 97% | 8.00E-34 | 46.15 | 253 | TJT18798.1 |
| dihydrolipoyllysine-residue succinyltransferase [Escherichia coli] | Escherichia coli | 131 | 131 | 97% | 8.00E-34 | 46.15 | 253 | WP_136748022.1 |
| dihydrolipoyllysine-residue succinyltransferase [Escherichia coli] | Escherichia coli | 131 | 131 | 97% | 8.00E-34 | 46.15 | 253 | EFH6296858.1 |
| TPA: dihydrolipoyllysine-residue succinyltransferase [Escherichia coli] | Escherichia coli | 131 | 131 | 97% | 8.00E-34 | 46.15 | 254 | HDC0239447.1 |
| dihydrolipoamide succinyltransferase [Escherichia coli] | Escherichia coli | 131 | 131 | 97% | 8.00E-34 | 46.15 | 251 | NYZ48214.1 |
| dihydrolipoyllysine-residue succinyltransferase [Escherichia coli] | Escherichia coli | 131 | 131 | 97% | 9.00E-34 | 46.15 | 253 | WP_136801371.1 |
| 2-oxo acid dehydrogenase subunit E2 [Acinetobacter baumannii] | Acinetobacter baumannii | 128 | 128 | 66% | 9.00E-34 | 56 | 146 | MDC4397105.1 |
| dihydrolipoyllysine-residue succinyltransferase [Escherichia coli] | Escherichia coli | 131 | 131 | 97% | 9.00E-34 | 46.15 | 257 | MCU7719730.1 |
| dihydrolipoyllysine-residue succinyltransferase [Escherichia coli] | Escherichia coli | 131 | 131 | 97% | 1.00E-33 | 46.15 | 259 | EIC1734575.1 |
| TPA: dihydrolipoyllysine-residue succinyltransferase [Escherichia coli] | Escherichia coli | 131 | 131 | 97% | 1.00E-33 | 46.15 | 261 | HAM4331889.1 |
| dihydrolipoyllysine-residue succinyltransferase [Escherichia coli O177] | Escherichia coli O177 | 130 | 130 | 96% | 1.00E-33 | 46.11 | 229 | EFA8854754.1 |
| dihydrolipoyllysine-residue succinyltransferase [Escherichia coli] | Escherichia coli | 131 | 220 | 97% | 1.00E-33 | 46.15 | 260 | WP_252391012.1 |
| dihydrolipoyllysine-residue succinyltransferase [Escherichia coli] | Escherichia coli | 131 | 220 | 97% | 1.00E-33 | 46.15 | 259 | EGE6907964.1 |
| dihydrolipoyllysine-residue succinyltransferase [Escherichia coli] | Escherichia coli | 131 | 220 | 97% | 1.00E-33 | 46.15 | 259 | WP_119682542.1 |
| dihydrolipoyllysine-residue succinyltransferase [Escherichia coli] | Escherichia coli | 131 | 221 | 97% | 1.00E-33 | 46.15 | 263 | WP_047654718.1 |
| dihydrolipoamide succinyltransferase [Escherichia coli] | Escherichia coli | 131 | 221 | 97% | 1.00E-33 | 46.15 | 263 | OWF26202.1 |
| dihydrolipoamide succinyltransferase component (E2) [Escherichia coli] | Escherichia coli | 131 | 131 | 97% | 1.00E-33 | 46.15 | 269 | VFT70387.1 |
| hypothetical protein ECZU26_29390 [Escherichia coli] | Escherichia coli | 131 | 220 | 97% | 1.00E-33 | 46.15 | 260 | GHL32114.1 |
| dihydrolipoyllysine-residue succinyltransferase [Escherichia coli] | Escherichia coli | 130 | 130 | 96% | 1.00E-33 | 46.11 | 230 | EGE7843444.1 |
| dihydrolipoyllysine-residue succinyltransferase [Escherichia coli O145:H28] | Escherichia coli O145:H28 | 131 | 220 | 97% | 1.00E-33 | 46.15 | 264 | EJH5270977.1 |
| TPA: dihydrolipoyllysine-residue succinyltransferase [Escherichia coli] | Escherichia coli | 131 | 220 | 97% | 1.00E-33 | 46.15 | 264 | HAN7662089.1 |
| TPA: dihydrolipoyllysine-residue succinyltransferase [Escherichia coli] | Escherichia coli | 131 | 220 | 97% | 1.00E-33 | 46.15 | 265 | HAP0635211.1 |
| dihydrolipoyllysine-residue succinyltransferase [Escherichia coli] | Escherichia coli | 130 | 130 | 95% | 1.00E-33 | 46.37 | 228 | WP_229024141.1 |
| 2-oxoglutarate dehydrogenase complex dihydrolipoyllysine-residue succinyltransferase [Escherichia coli] | Escherichia coli | 131 | 131 | 97% | 1.00E-33 | 46.15 | 269 | WP_115969540.1 |
| dihydrolipoamide succinyltransferase component (E2) [Escherichia coli] | Escherichia coli | 131 | 131 | 97% | 1.00E-33 | 46.15 | 269 | STG69599.1 |
| 2-oxoglutarate dehydrogenase complex dihydrolipoyllysine-residue succinyltransferase [Escherichia coli] | Escherichia coli | 131 | 131 | 97% | 1.00E-33 | 46.15 | 269 | WP_249524430.1 |
| 2-oxoglutarate dehydrogenase complex dihydrolipoyllysine-residue succinyltransferase [Escherichia coli] | Escherichia coli | 134 | 134 | 97% | 1.00E-33 | 46.7 | 405 | WP_097415199.1 |
| 2-oxoglutarate dehydrogenase complex dihydrolipoyllysine-residue succinyltransferase [Escherichia coli] | Escherichia coli | 131 | 131 | 97% | 1.00E-33 | 46.15 | 270 | WP_250382326.1 |
| 2-oxoglutarate dehydrogenase complex dihydrolipoyllysine-residue succinyltransferase [Escherichia coli] | Escherichia coli | 131 | 131 | 97% | 1.00E-33 | 46.15 | 280 | WP_320759799.1 |
| 2-oxoglutarate dehydrogenase complex dihydrolipoyllysine-residue succinyltransferase [Escherichia coli] | Escherichia coli | 134 | 224 | 97% | 1.00E-33 | 46.7 | 405 | MCW7185905.1 |
| 2-oxoglutarate dehydrogenase complex dihydrolipoyllysine-residue succinyltransferase [Escherichia coli] | Escherichia coli | 131 | 131 | 97% | 1.00E-33 | 46.15 | 275 | WP_136750337.1 |
| 2-oxoglutarate dehydrogenase complex dihydrolipoyllysine-residue succinyltransferase [Escherichia coli] | Escherichia coli | 131 | 131 | 97% | 2.00E-33 | 46.15 | 274 | MDY8964183.1 |
| 2-oxoglutarate dehydrogenase complex dihydrolipoyllysine-residue succinyltransferase [Escherichia coli] | Escherichia coli | 131 | 131 | 97% | 2.00E-33 | 46.15 | 274 | RIC38964.1 |
| 2-oxoglutarate dehydrogenase complex dihydrolipoyllysine-residue succinyltransferase [Escherichia coli] | Escherichia coli | 131 | 131 | 97% | 2.00E-33 | 46.15 | 279 | WP_247166683.1 |
| TPA: 2-oxoglutarate dehydrogenase complex dihydrolipoyllysine-residue succinyltransferase [Shigella flexneri 2a] | Shigella flexneri 2a | 131 | 131 | 97% | 2.00E-33 | 46.15 | 273 | HFT4085970.1 |
| 2-oxoglutarate dehydrogenase complex dihydrolipoyllysine-residue succinyltransferase [Escherichia coli] | Escherichia coli | 131 | 131 | 97% | 2.00E-33 | 46.15 | 282 | MDY9040892.1 |
| 2-oxoglutarate dehydrogenase complex dihydrolipoyllysine-residue succinyltransferase [Escherichia coli] | Escherichia coli | 131 | 131 | 97% | 2.00E-33 | 46.15 | 286 | WP_047081876.1 |
| 2-oxoglutarate dehydrogenase complex dihydrolipoyllysine-residue succinyltransferase [Escherichia coli] | Escherichia coli | 131 | 131 | 97% | 2.00E-33 | 46.15 | 286 | WP_060643349.1 |
| 2-oxo acid dehydrogenase subunit E2 [Klebsiella pneumoniae] | Klebsiella pneumoniae | 127 | 127 | 66% | 2.00E-33 | 56 | 167 | WP_211540998.1 |
| 2-oxoglutarate dehydrogenase complex dihydrolipoyllysine-residue succinyltransferase [Escherichia coli] | Escherichia coli | 131 | 131 | 97% | 2.00E-33 | 46.15 | 295 | NAG00763.1 |
| dihydrolipoamide succinyltransferase [Shigella sonnei] | Shigella sonnei | 131 | 221 | 97% | 2.00E-33 | 46.15 | 291 | CSF30971.1 |
| 2-oxoglutarate dehydrogenase complex dihydrolipoyllysine-residue succinyltransferase [Escherichia coli] | Escherichia coli | 131 | 131 | 97% | 2.00E-33 | 46.15 | 292 | WP_194498297.1 |
| dihydrolipoyllysine-residue succinyltransferase [Escherichia coli] | Escherichia coli | 129 | 129 | 93% | 2.00E-33 | 46.29 | 226 | WP_074164196.1 |
| 2-oxoglutarate dehydrogenase complex dihydrolipoyllysine-residue succinyltransferase [Escherichia coli] | Escherichia coli | 131 | 131 | 97% | 2.00E-33 | 46.15 | 287 | EEZ2060122.1 |
| hypothetical protein EIMP300_69380 [Escherichia coli] | Escherichia coli | 128 | 128 | 93% | 2.00E-33 | 45.98 | 187 | BBU85538.1 |
| TPA: 2-oxoglutarate dehydrogenase complex dihydrolipoyllysine-residue succinyltransferase [Escherichia coli] | Escherichia coli | 132 | 132 | 97% | 3.00E-33 | 46.15 | 312 | HCO0761733.1 |
| TPA: 2-oxoglutarate dehydrogenase complex dihydrolipoyllysine-residue succinyltransferase [Escherichia coli] | Escherichia coli | 132 | 132 | 95% | 3.00E-33 | 46.07 | 318 | HCJ6193756.1 |
| 2-oxoglutarate dehydrogenase complex dihydrolipoyllysine-residue succinyltransferase [Escherichia coli] | Escherichia coli | 131 | 131 | 97% | 3.00E-33 | 46.15 | 303 | EEY6784672.1 |
| 2-oxoglutarate dehydrogenase complex dihydrolipoyllysine-residue succinyltransferase [Escherichia coli] | Escherichia coli | 131 | 131 | 97% | 3.00E-33 | 46.15 | 300 | MBA1840094.1 |
| 2-oxoglutarate dehydrogenase complex dihydrolipoyllysine-residue succinyltransferase [Escherichia coli] | Escherichia coli | 132 | 132 | 95% | 3.00E-33 | 46.37 | 335 | MCF2049886.1 |
| 2-oxoglutarate dehydrogenase complex dihydrolipoyllysine-residue succinyltransferase [Escherichia coli] | Escherichia coli | 131 | 131 | 95% | 3.00E-33 | 46.07 | 318 | MBW2856955.1 |
| 2-oxoglutarate dehydrogenase complex dihydrolipoyllysine-residue succinyltransferase [Escherichia coli] | Escherichia coli | 133 | 133 | 97% | 3.00E-33 | 46.15 | 405 | WP_192459758.1 |
| 2-oxoglutarate dehydrogenase complex dihydrolipoyllysine-residue succinyltransferase [Shigella sonnei] | Shigella sonnei | 132 | 132 | 95% | 3.00E-33 | 46.37 | 334 | WP_077134249.1 |
| TPA: 2-oxoglutarate dehydrogenase complex dihydrolipoyllysine-residue succinyltransferase [Escherichia coli] | Escherichia coli | 132 | 132 | 95% | 3.00E-33 | 46.37 | 336 | HDI5831311.1 |
| 2-oxoglutarate dehydrogenase complex dihydrolipoyllysine-residue succinyltransferase [Escherichia coli] | Escherichia coli | 131 | 221 | 97% | 3.00E-33 | 46.15 | 322 | WP_194154148.1 |
| 2-oxoglutarate dehydrogenase complex dihydrolipoyllysine-residue succinyltransferase [Escherichia coli] | Escherichia coli | 131 | 221 | 97% | 3.00E-33 | 46.15 | 322 | MCU8601182.1 |
| 2-oxoglutarate dehydrogenase complex dihydrolipoyllysine-residue succinyltransferase [Escherichia coli] | Escherichia coli | 131 | 221 | 97% | 3.00E-33 | 46.15 | 321 | EEY6693670.1 |
| 2-oxoglutarate dehydrogenase complex dihydrolipoyllysine-residue succinyltransferase [Vibrio sp. FNV 38] | Vibrio sp. FNV 38 | 132 | 132 | 95% | 3.00E-33 | 46.37 | 327 | MDR9828261.1 |
| TPA: 2-oxoglutarate dehydrogenase complex dihydrolipoyllysine-residue succinyltransferase [Escherichia coli] | Escherichia coli | 132 | 132 | 95% | 3.00E-33 | 46.37 | 327 | HDH9429852.1 |
| 2-oxoglutarate dehydrogenase complex dihydrolipoyllysine-residue succinyltransferase [Escherichia coli] | Escherichia coli | 131 | 131 | 97% | 4.00E-33 | 46.15 | 324 | EER8331471.1 |
| 2-oxoglutarate dehydrogenase complex dihydrolipoyllysine-residue succinyltransferase [Escherichia coli] | Escherichia coli | 131 | 131 | 97% | 4.00E-33 | 46.15 | 324 | MDO4358214.1 |
| 2-oxoglutarate dehydrogenase complex dihydrolipoyllysine-residue succinyltransferase [Escherichia coli] | Escherichia coli | 132 | 222 | 97% | 4.00E-33 | 46.15 | 331 | WP_042068848.1 |
| TPA: 2-oxoglutarate dehydrogenase complex dihydrolipoyllysine-residue succinyltransferase [Escherichia coli] | Escherichia coli | 131 | 131 | 95% | 4.00E-33 | 46.37 | 328 | HDH2834759.1 |
| dihydrolipoamide succinyltransferase component (E2) [Escherichia coli] | Escherichia coli | 132 | 222 | 97% | 4.00E-33 | 46.15 | 332 | STM59242.1 |
| 2-oxoglutarate dehydrogenase complex dihydrolipoyllysine-residue succinyltransferase [Escherichia coli] | Escherichia coli | 133 | 133 | 97% | 4.00E-33 | 46.7 | 405 | EEZ9068234.1 |
| 2-oxoglutarate dehydrogenase complex dihydrolipoyllysine-residue succinyltransferase [Escherichia coli] | Escherichia coli | 131 | 221 | 97% | 4.00E-33 | 46.15 | 318 | MCX0506393.1 |
| dihydrolipoamide succinyltransferase component (E2) [Salmonella enterica subsp. enterica] | Salmonella enterica subsp. enterica | 128 | 128 | 96% | 4.00E-33 | 45.56 | 212 | VEA37457.1 |

##

## Supplemental Table 12. Protein BLAST results based on consensus sequence of automated model building on a 4.75 Å low-pass filtered volume map

| **Description** | **Scientific Name** | **Max Score** | **Total Score** | **Query Cover** | **E value** | **Per. ident** | **Acc. Len** | **Accession** |
| --- | --- | --- | --- | --- | --- | --- | --- | --- |
| dihydrolipoyllysine-residue succinyltransferase [Escherichia coli] | Escherichia coli | 134 | 134 | 94% | 5.00E-35 | 39.57 | 226 | WP_074164196.1 |
| dihydrolipoyllysine-residue succinyltransferase [Escherichia coli O157:H7] | Escherichia coli O157:H7 | 132 | 132 | 96% | 3.00E-34 | 38.72 | 231 | EEW1174705.1 |
| dihydrolipoyllysine-residue succinyltransferase [Escherichia] | Escherichia | 132 | 132 | 96% | 3.00E-34 | 38.72 | 236 | WP_000629458.1 |
| dihydrolipoyllysine-residue succinyltransferase [Escherichia coli] | Escherichia coli | 132 | 132 | 96% | 3.00E-34 | 38.72 | 233 | WP_136823828.1 |
| dihydrolipoyllysine-residue succinyltransferase [Escherichia coli] | Escherichia coli | 132 | 132 | 96% | 3.00E-34 | 38.72 | 232 | ENT2450297.1 |
| dihydrolipoyllysine-residue succinyltransferase [Escherichia coli] | Escherichia coli | 132 | 132 | 96% | 3.00E-34 | 38.72 | 234 | WP_149465242.1 |
| dihydrolipoyllysine-residue succinyltransferase [Escherichia coli] | Escherichia coli | 132 | 132 | 96% | 3.00E-34 | 38.72 | 235 | WP_122792276.1 |
| dihydrolipoyllysine-residue succinyltransferase [Escherichia coli] | Escherichia coli | 132 | 132 | 96% | 4.00E-34 | 38.72 | 237 | WP_252389303.1 |
| dihydrolipoyllysine-residue succinyltransferase [Escherichia coli] | Escherichia coli | 132 | 132 | 96% | 4.00E-34 | 38.72 | 238 | EFO3983716.1 |
| hypothetical protein ECZU22_19390 [Escherichia coli] | Escherichia coli | 131 | 131 | 94% | 4.00E-34 | 39.13 | 227 | GHL08040.1 |
| dihydrolipoyllysine-residue succinyltransferase [Escherichia coli] | Escherichia coli | 132 | 132 | 96% | 4.00E-34 | 38.72 | 235 | WP_250378311.1 |
| dihydrolipoyllysine-residue succinyltransferase [Escherichia coli] | Escherichia coli | 132 | 132 | 96% | 4.00E-34 | 38.72 | 242 | WP_149518815.1 |
| dihydrolipoyllysine-residue succinyltransferase [Escherichia coli] | Escherichia coli | 132 | 132 | 96% | 4.00E-34 | 38.72 | 243 | EEX5691720.1 |
| dihydrolipoyllysine-residue succinyltransferase [Escherichia coli] | Escherichia coli | 132 | 132 | 96% | 4.00E-34 | 38.72 | 239 | EEZ3479170.1 |
| TPA: dihydrolipoyllysine-residue succinyltransferase [Escherichia coli] | Escherichia coli | 132 | 132 | 96% | 4.00E-34 | 38.72 | 240 | HDY2775515.1 |
| dihydrolipoyllysine-residue succinyltransferase [Escherichia coli] | Escherichia coli | 131 | 131 | 94% | 4.00E-34 | 39.13 | 227 | EIH2436553.1 |
| dihydrolipoyllysine-residue succinyltransferase [Escherichia coli O157:H7] | Escherichia coli O157:H7 | 132 | 132 | 96% | 4.00E-34 | 38.72 | 240 | EFC3643281.1 |
| dihydrolipoyllysine-residue succinyltransferase [Escherichia coli] | Escherichia coli | 131 | 131 | 94% | 4.00E-34 | 39.13 | 226 | EER2248380.1 |
| TPA: dihydrolipoyllysine-residue succinyltransferase [Escherichia coli] | Escherichia coli | 132 | 132 | 96% | 4.00E-34 | 38.72 | 244 | HBB1186217.1 |
| dihydrolipoyllysine-residue succinyltransferase [Escherichia coli] | Escherichia coli | 132 | 132 | 96% | 4.00E-34 | 38.72 | 245 | WP_136775487.1 |
| dihydrolipoyllysine-residue succinyltransferase [Escherichia coli O145:H28] | Escherichia coli O145:H28 | 132 | 132 | 96% | 4.00E-34 | 38.72 | 246 | EJH5192981.1 |
| dihydrolipoyllysine-residue succinyltransferase [Escherichia coli] | Escherichia coli | 132 | 132 | 96% | 4.00E-34 | 38.72 | 247 | WP_185168965.1 |
| dihydrolipoyllysine-residue succinyltransferase [Escherichia coli] | Escherichia coli | 131 | 131 | 94% | 5.00E-34 | 39.13 | 228 | WP_229024141.1 |
| dihydrolipoyllysine-residue succinyltransferase [Escherichia coli] | Escherichia coli | 132 | 132 | 96% | 5.00E-34 | 38.72 | 248 | KAA0647049.1 |
| dihydrolipoyllysine-residue succinyltransferase [Escherichia coli] | Escherichia coli | 132 | 132 | 96% | 5.00E-34 | 38.72 | 243 | TJB60735.1 |
| dihydrolipoyllysine-residue succinyltransferase [Escherichia coli] | Escherichia coli | 132 | 132 | 96% | 5.00E-34 | 38.72 | 248 | TJB30469.1 |
| dihydrolipoyllysine-residue succinyltransferase [Escherichia coli] | Escherichia coli | 131 | 131 | 96% | 5.00E-34 | 38.72 | 241 | EIH9592508.1 |
| TPA: dihydrolipoyllysine-residue succinyltransferase [Escherichia coli] | Escherichia coli | 132 | 132 | 96% | 5.00E-34 | 38.72 | 250 | HBI3672065.1 |
| dihydrolipoyllysine-residue succinyltransferase [Escherichia coli O177] | Escherichia coli O177 | 131 | 131 | 94% | 5.00E-34 | 39.13 | 229 | EFA8854754.1 |
| dihydrolipoyllysine-residue succinyltransferase [Salmonella enterica subsp. enterica] | Salmonella enterica subsp. enterica | 132 | 132 | 96% | 5.00E-34 | 38.72 | 249 | EHK8207480.1 |
| dihydrolipoyllysine-residue succinyltransferase [Escherichia coli] | Escherichia coli | 132 | 132 | 96% | 5.00E-34 | 38.72 | 255 | WP_042066964.1 |
| dihydrolipoyllysine-residue succinyltransferase [Escherichia coli] | Escherichia coli | 132 | 132 | 96% | 5.00E-34 | 38.72 | 252 | MDY9032137.1 |
| TPA: dihydrolipoyllysine-residue succinyltransferase [Escherichia coli] | Escherichia coli | 132 | 132 | 96% | 5.00E-34 | 38.72 | 252 | HBD1696035.1 |
| dihydrolipoyllysine-residue succinyltransferase [Escherichia coli] | Escherichia coli | 131 | 131 | 96% | 5.00E-34 | 38.89 | 230 | EGE7843444.1 |
| hypothetical protein ECZU26_29390 [Escherichia coli] | Escherichia coli | 132 | 132 | 96% | 5.00E-34 | 38.72 | 260 | GHL32114.1 |
| dihydrolipoyllysine-residue succinyltransferase [Escherichia coli] | Escherichia coli | 132 | 132 | 96% | 5.00E-34 | 38.72 | 253 | TJT18798.1 |
| dihydrolipoyllysine-residue succinyltransferase [Escherichia coli] | Escherichia coli | 132 | 132 | 96% | 5.00E-34 | 38.72 | 253 | WP_136748022.1 |
| dihydrolipoyllysine-residue succinyltransferase [Escherichia coli] | Escherichia coli | 132 | 132 | 96% | 5.00E-34 | 38.72 | 253 | EFH6296858.1 |
| dihydrolipoyllysine-residue succinyltransferase [Escherichia coli] | Escherichia coli | 132 | 132 | 96% | 5.00E-34 | 38.72 | 257 | MCU7719730.1 |
| dihydrolipoyllysine-residue succinyltransferase [Escherichia coli] | Escherichia coli | 132 | 132 | 96% | 5.00E-34 | 38.72 | 253 | WP_347079825.1 |
| TPA: dihydrolipoyllysine-residue succinyltransferase [Escherichia coli] | Escherichia coli | 132 | 132 | 96% | 5.00E-34 | 38.72 | 261 | HAM4331889.1 |
| dihydrolipoyllysine-residue succinyltransferase [Escherichia coli] | Escherichia coli | 132 | 132 | 96% | 6.00E-34 | 38.72 | 259 | EGE6907964.1 |
| dihydrolipoyllysine-residue succinyltransferase [Escherichia coli] | Escherichia coli | 132 | 132 | 96% | 6.00E-34 | 38.72 | 259 | WP_119682542.1 |
| dihydrolipoyllysine-residue succinyltransferase [Escherichia coli] | Escherichia coli | 132 | 132 | 96% | 6.00E-34 | 38.72 | 250 | WP_323653019.1 |
| dihydrolipoyllysine-residue succinyltransferase [Escherichia coli] | Escherichia coli | 132 | 132 | 96% | 6.00E-34 | 38.72 | 256 | MCJ8642909.1 |
| TPA: dihydrolipoyllysine-residue succinyltransferase [Escherichia coli] | Escherichia coli | 132 | 132 | 96% | 6.00E-34 | 38.72 | 254 | HDC0239447.1 |
| dihydrolipoyllysine-residue succinyltransferase [Escherichia coli] | Escherichia coli | 132 | 132 | 96% | 6.00E-34 | 38.72 | 260 | WP_252391012.1 |
| dihydrolipoyllysine-residue succinyltransferase [Escherichia coli O145:H28] | Escherichia coli O145:H28 | 132 | 132 | 96% | 6.00E-34 | 38.72 | 264 | EJH5270977.1 |
| dihydrolipoyllysine-residue succinyltransferase [Escherichia coli] | Escherichia coli | 131 | 131 | 94% | 6.00E-34 | 39.13 | 227 | ENU3117923.1 |
| TPA: dihydrolipoyllysine-residue succinyltransferase [Escherichia coli] | Escherichia coli | 132 | 132 | 96% | 7.00E-34 | 38.72 | 265 | HAP0635211.1 |
| dihydrolipoyllysine-residue succinyltransferase [Escherichia coli] | Escherichia coli | 131 | 131 | 96% | 7.00E-34 | 38.72 | 253 | WP_136801371.1 |
| TPA: dihydrolipoyllysine-residue succinyltransferase [Escherichia coli] | Escherichia coli | 132 | 132 | 96% | 7.00E-34 | 38.72 | 264 | HAN7662089.1 |
| dihydrolipoyllysine-residue succinyltransferase [Escherichia coli] | Escherichia coli | 131 | 131 | 96% | 7.00E-34 | 38.72 | 259 | EIC1734575.1 |
| 2-oxoglutarate dehydrogenase complex dihydrolipoyllysine-residue succinyltransferase [Escherichia coli] | Escherichia coli | 132 | 132 | 96% | 7.00E-34 | 38.72 | 280 | WP_320759799.1 |
| dihydrolipoamide succinyltransferase component (E2) [Escherichia coli] | Escherichia coli | 132 | 132 | 96% | 8.00E-34 | 38.72 | 269 | STG69599.1 |
| 2-oxoglutarate dehydrogenase complex dihydrolipoyllysine-residue succinyltransferase [Escherichia coli] | Escherichia coli | 132 | 132 | 96% | 8.00E-34 | 38.72 | 285 | MSH88871.1 |
| dihydrolipoamide succinyltransferase component (E2) [Escherichia coli] | Escherichia coli | 132 | 132 | 96% | 8.00E-34 | 38.72 | 269 | VFT70387.1 |
| 2-oxoglutarate dehydrogenase complex dihydrolipoyllysine-residue succinyltransferase [Escherichia coli] | Escherichia coli | 132 | 132 | 96% | 8.00E-34 | 38.72 | 279 | WP_247166683.1 |
| dihydrolipoyllysine-residue succinyltransferase [Escherichia coli] | Escherichia coli | 131 | 131 | 96% | 8.00E-34 | 38.72 | 263 | WP_047654718.1 |
| dihydrolipoamide succinyltransferase [Escherichia coli] | Escherichia coli | 131 | 131 | 96% | 8.00E-34 | 38.72 | 263 | OWF26202.1 |
| 2-oxoglutarate dehydrogenase complex dihydrolipoyllysine-residue succinyltransferase [Escherichia coli] | Escherichia coli | 132 | 132 | 96% | 8.00E-34 | 38.72 | 270 | WP_250382326.1 |
| 2-oxoglutarate dehydrogenase complex dihydrolipoyllysine-residue succinyltransferase [Escherichia coli] | Escherichia coli | 132 | 132 | 96% | 8.00E-34 | 38.72 | 269 | WP_115969540.1 |
| 2-oxoglutarate dehydrogenase complex dihydrolipoyllysine-residue succinyltransferase [Escherichia coli] | Escherichia coli | 132 | 132 | 96% | 8.00E-34 | 38.72 | 269 | WP_249524430.1 |
| 2-oxoglutarate dehydrogenase complex dihydrolipoyllysine-residue succinyltransferase [Escherichia coli] | Escherichia coli | 132 | 132 | 96% | 8.00E-34 | 38.72 | 274 | RIC38964.1 |
| 2-oxoglutarate dehydrogenase complex dihydrolipoyllysine-residue succinyltransferase [Escherichia coli] | Escherichia coli | 132 | 132 | 96% | 8.00E-34 | 38.72 | 282 | MDY9040892.1 |
| 2-oxoglutarate dehydrogenase complex dihydrolipoyllysine-residue succinyltransferase [Escherichia coli] | Escherichia coli | 132 | 132 | 96% | 9.00E-34 | 38.72 | 275 | WP_136750337.1 |
| 2-oxoglutarate dehydrogenase complex dihydrolipoyllysine-residue succinyltransferase [Escherichia coli] | Escherichia coli | 132 | 132 | 96% | 9.00E-34 | 38.72 | 286 | WP_060643349.1 |
| 2-oxoglutarate dehydrogenase complex dihydrolipoyllysine-residue succinyltransferase [Escherichia coli] | Escherichia coli | 132 | 132 | 96% | 9.00E-34 | 38.72 | 286 | WP_047081876.1 |
| 2-oxoglutarate dehydrogenase complex dihydrolipoyllysine-residue succinyltransferase [Escherichia coli] | Escherichia coli | 132 | 132 | 96% | 1.00E-33 | 38.72 | 287 | EEZ2060122.1 |
| 2-oxoglutarate dehydrogenase complex dihydrolipoyllysine-residue succinyltransferase [Escherichia coli] | Escherichia coli | 132 | 132 | 96% | 1.00E-33 | 38.72 | 274 | MDY8964183.1 |
| dihydrolipoamide succinyltransferase [Shigella sonnei] | Shigella sonnei | 132 | 132 | 96% | 1.00E-33 | 38.72 | 291 | CSF30971.1 |
| 2-oxoglutarate dehydrogenase complex dihydrolipoyllysine-residue succinyltransferase [Escherichia coli] | Escherichia coli | 134 | 134 | 96% | 1.00E-33 | 39.15 | 405 | MCW7185905.1 |
| 2-oxoglutarate dehydrogenase complex dihydrolipoyllysine-residue succinyltransferase [Escherichia coli] | Escherichia coli | 132 | 132 | 96% | 1.00E-33 | 38.72 | 292 | WP_194498297.1 |
| dihydrolipoyllysine-residue succinyltransferase [Salmonella enterica] | Salmonella enterica | 130 | 130 | 97% | 1.00E-33 | 38.98 | 232 | WP_080094185.1 |
| dihydrolipoyllysine-residue succinyltransferase [Salmonella enterica] | Salmonella enterica | 130 | 130 | 97% | 1.00E-33 | 38.98 | 231 | WP_128277741.1 |
| dihydrolipoyllysine-residue succinyltransferase [Salmonella enterica subsp. enterica serovar Typhimurium] | Salmonella enterica subsp. enterica serovar Typhimurium | 130 | 130 | 97% | 1.00E-33 | 38.98 | 233 | ECY4747990.1 |
| dihydrolipoyllysine-residue succinyltransferase [Salmonella enterica] | Salmonella enterica | 130 | 130 | 97% | 1.00E-33 | 38.98 | 241 | EEN7692697.1 |
| 2-oxoglutarate dehydrogenase complex dihydrolipoyllysine-residue succinyltransferase [Escherichia coli] | Escherichia coli | 132 | 132 | 96% | 1.00E-33 | 38.72 | 295 | NAG00763.1 |
| 2-oxoglutarate dehydrogenase complex dihydrolipoyllysine-residue succinyltransferase [Escherichia coli] | Escherichia coli | 132 | 132 | 96% | 2.00E-33 | 38.72 | 303 | EEY6784672.1 |
| dihydrolipoyllysine-residue succinyltransferase [Salmonella enterica subsp. enterica serovar Virginia] | Salmonella enterica subsp. enterica serovar Virginia | 130 | 130 | 97% | 2.00E-33 | 38.98 | 237 | MEA7606579.1 |
| 2-oxoglutarate dehydrogenase complex dihydrolipoyllysine-residue succinyltransferase [Escherichia coli] | Escherichia coli | 132 | 132 | 96% | 2.00E-33 | 38.72 | 300 | MBA1840094.1 |
| dihydrolipoyllysine-residue succinyltransferase [Salmonella enterica subsp. enterica serovar Enteritidis] | Salmonella enterica subsp. enterica serovar Enteritidis | 130 | 130 | 97% | 2.00E-33 | 38.98 | 233 | EIF4604241.1 |
| dihydrolipoyllysine-residue succinyltransferase [Salmonella enterica subsp. enterica serovar Reading] | Salmonella enterica subsp. enterica serovar Reading | 130 | 130 | 97% | 2.00E-33 | 38.98 | 241 | EDV1620825.1 |
| dihydrolipoyllysine-residue succinyltransferase [Escherichia coli] | Escherichia coli | 129 | 129 | 91% | 2.00E-33 | 39.56 | 221 | EFA1784611.1 |
| dihydrolipoyllysine-residue succinyltransferase [Salmonella enterica subsp. enterica serovar Anatum] | Salmonella enterica subsp. enterica serovar Anatum | 129 | 129 | 94% | 2.00E-33 | 38.7 | 226 | EDV9283131.1 |
| 2-oxoglutarate dehydrogenase complex dihydrolipoyllysine-residue succinyltransferase [Escherichia coli] | Escherichia coli | 134 | 134 | 96% | 2.00E-33 | 39.15 | 405 | ELW7327477.1 |
| TPA: 2-oxoglutarate dehydrogenase complex dihydrolipoyllysine-residue succinyltransferase [Escherichia coli] | Escherichia coli | 132 | 132 | 96% | 2.00E-33 | 38.72 | 312 | HCO0761733.1 |
| 2-oxoglutarate dehydrogenase complex dihydrolipoyllysine-residue succinyltransferase [Escherichia coli] | Escherichia coli | 132 | 132 | 96% | 2.00E-33 | 38.72 | 318 | MCX0506393.1 |
| 2-oxoglutarate dehydrogenase complex dihydrolipoyllysine-residue succinyltransferase [Escherichia coli] | Escherichia coli | 134 | 134 | 96% | 2.00E-33 | 38.72 | 405 | WP_192459758.1 |
| TPA: 2-oxoglutarate dehydrogenase complex dihydrolipoyllysine-residue succinyltransferase [Escherichia coli] | Escherichia coli | 134 | 134 | 96% | 2.00E-33 | 39.15 | 405 | HAY0435431.1 |
| 2-oxoglutarate dehydrogenase complex dihydrolipoyllysine-residue succinyltransferase [Escherichia coli] | Escherichia coli | 134 | 134 | 96% | 2.00E-33 | 39.15 | 405 | WP_286615287.1 |
| 2-oxoglutarate dehydrogenase complex dihydrolipoyllysine-residue succinyltransferase [Escherichia coli] | Escherichia coli | 134 | 134 | 96% | 2.00E-33 | 39.15 | 405 | WP_220405777.1 |
| 2-oxoglutarate dehydrogenase complex dihydrolipoyllysine-residue succinyltransferase [Escherichia coli] | Escherichia coli | 132 | 132 | 96% | 3.00E-33 | 38.72 | 322 | WP_194154148.1 |
| 2-oxoglutarate dehydrogenase complex dihydrolipoyllysine-residue succinyltransferase [Escherichia coli] | Escherichia coli | 132 | 132 | 96% | 3.00E-33 | 38.72 | 322 | MCU8601182.1 |
| 2-oxoglutarate dehydrogenase complex dihydrolipoyllysine-residue succinyltransferase [Escherichia coli] | Escherichia coli | 132 | 132 | 96% | 3.00E-33 | 38.72 | 321 | EEY6693670.1 |
| 2-oxoglutarate dehydrogenase complex dihydrolipoyllysine-residue succinyltransferase [Escherichia coli] | Escherichia coli | 132 | 132 | 96% | 3.00E-33 | 38.72 | 324 | EER8331471.1 |
| 2-oxoglutarate dehydrogenase complex dihydrolipoyllysine-residue succinyltransferase [Escherichia coli] | Escherichia coli | 132 | 132 | 96% | 3.00E-33 | 38.72 | 324 | MDO4358214.1 |
| 2-oxoglutarate dehydrogenase complex dihydrolipoyllysine-residue succinyltransferase [Citrobacter koseri] | Citrobacter koseri | 133 | 133 | 96% | 3.00E-33 | 38.72 | 406 | WP_200077452.1 |
| dihydrolipoyllysine-residue succinyltransferase [Salmonella enterica subsp. enterica serovar Wilhelmsburg] | Salmonella enterica subsp. enterica serovar Wilhelmsburg | 130 | 130 | 97% | 3.00E-33 | 38.98 | 257 | TGC90782.1 |
